# (Aza)Pentacenes Clipped into a Ring: Stabilization of Large (Aza)Acenes

**DOI:** 10.1002/anie.202015348

**Published:** 2021-02-03

**Authors:** Lukas Ahrens, Olena Tverskoy, Svenja Weigold, Michael Ganschow, Frank Rominger, Jan Freudenberg, Uwe H. F. Bunz

**Affiliations:** ^1^ Organisch-Chemisches Institut Ruprecht-Karls-Universität Heidelberg Im Neuenheimer Feld 270 69120 Heidelberg Germany; ^2^ Centre for Advanced Materials Im Neuenheimer Feld 225 69120 Heidelberg Germany

**Keywords:** acenes, organic materials, semiconductors, stabilization, X-ray diffraction

## Abstract

A doubly alkylene bridged 6,13‐diphenylpentacene and analogously bridged azapentacenes were prepared; they are persistent. The doubly bridged azapentacenes display superior photochemical, oxidative and thermal stabilities compared to azapentacenes protected by bis(TIPS‐ethynyl)‐substituents—clipping an azaacene into a large ring is a viable complement in stabilization.

## Graphical abstract

Doubly alkylene bridged tetraazahexacene and (aza)pentacenes were synthesized, introducing an alternative and complementary method of stabilization of large (hetero)acenes to trialkylsilylalkynylation. The examined materials display superior photochemical and thermal stabilities compared to their TIPS‐ethynyl substituted congeners.

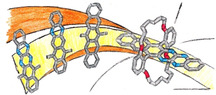

Stabilization and solubilization of larger acenes and heteroacenes, that is, ≥5 rings, is challenging, yet important to fully unlock their properties.^[1]^ Aryl substituents attached at strategic positions of a large acene fulfills this need to a degree. Yet, the use of bis(trialkylsilylethynyl)‐groups in 2001 changed the situation.^[2]^ The silylethynyl substituents enjoy a near monopoly in the stabilization of larger (hetero)acenes—heptacene can be stabilized and solubilized with just two of them.^[3]^ Additional auxiliary arene substituents are necessary for nonacenes to survive.^[4]^ However, there should be alternative stabilization modes of similar efficiency. Kobayashi et al. demonstrated doubly alkylene bridged anthracenes as photoemitters with improved photostability when compared to 5,9‐diphenylanthracene.^[5]^ Yet, these systems were not compared to their analogous 5,9‐bis(tri*iso*propylsilylethyny)anthracenes to gauge relative stability. Double bridging was also employed to twist anthracene,^[6]^ to ring perylenebisimides^[7]^ and to shield polythiophene derivatives.^[8]^

Kobayashi's encapsulation should be applicable to larger acenes. Herein, we present stable and soluble, modularly synthesized doubly bridged (aza)pentacenes **5**–**9** (Scheme 1). Starting from **1**, double Suzuki coupling furnishes **2**. Removal of the methyl groups with BBr_3_ is followed by transformation of the resorcinic intermediate with 1,7‐dibromoheptane in DMF (K_2_CO_3_ as base) to furnish jacketed pentacene **5**. The double cyclization was performed at concentrations of 5 mmol L^−1^. For the azaacenes **6** and **7**, the bridged diaminonaphthalene **4** was obtained from naphthothiadiazoloquinone. Addition of lithiated 1,3‐dimethoxybenzene and reduction with sodium hypophosphite, followed by the opening of the thiadiazole ring by SmI_2_ gives **4**. Diamino‐naphthalene **4** couples under established Pd‐catalyzed conditions[^9^, ^10^, ^11a^, ^11b^, ^12^] with 2,3‐dihaloarenes to give the azapentacenes **6** and **7**. If the doubly bridged diaminoanthracene is employed, **8** results, while **9** is obtained by coupling of *ortho*‐phenylenediamine with the encapsulated 2,3‐dibromoanthracene. **6**–**9** form as the *N*,*N′*‐dihydro‐compounds—these are oxidized by MnO_2_ into the azaacenes.

**Scheme 1:**
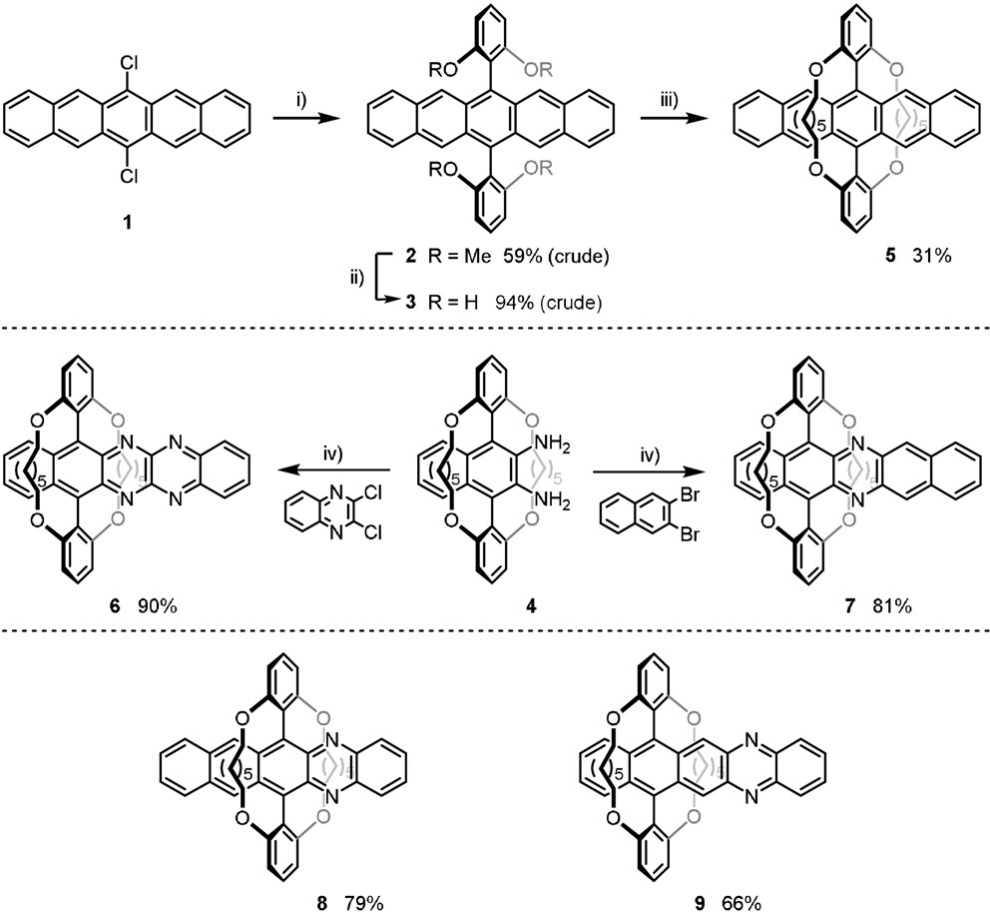
Synthesis of doubly alkylene bridged (aza)pentacenes **5**–**9**. Conditions: i) ArB(OH)_2_, K_3_PO_4_, Pd_2_(dba)_3_, XPhos, 1,4‐dioxane, H_2_O, 100 °C., 7 d; ii) BBr_3_, DCM, −78 °C→r.t., 2 d→7 d; iii) K_2_CO_3_, Br(CH_2_)_7_Br, DMF, 40 °C→80 °C, 3 d; iv) 1.) Cs_2_CO_3_, RuPhos Pd G1 (10 mol %), toluene, 110 °C, 15 h; 2.) MnO_2_, DCM, r.t., 30 min.

The consanguine TIPS‐ethynyl(aza)acenes are literature known and were prepared as reference substances.[^2^, ^11^, ^12^] Figure 1 displays a photograph of dilute solutions of **5**–**9** and of **5_TIPS_**‐**9_TIPS_**.The visual colors are similar—their slight variations (cf. **5** and **5_TIPS_**) are due to the TIPS‐ethynyl groups enlarging the conjugated π‐system. The doubly alkylene bridged (aza)acenes display in general broader and blue‐shifted features in the Uv‐vis spectra (Figure 2). This is not the case for **9**—*λ*_max_ of **9_TIPS_** is blue shifted, possibly due to an increased donor‐acceptor character of **9** in comparison to **9_TIPS_**. The doubly bridged azaacenes appear non‐emissive, similar to their TIPS‐congeners, only **5** fluoresces notably.

**Figure 1:**
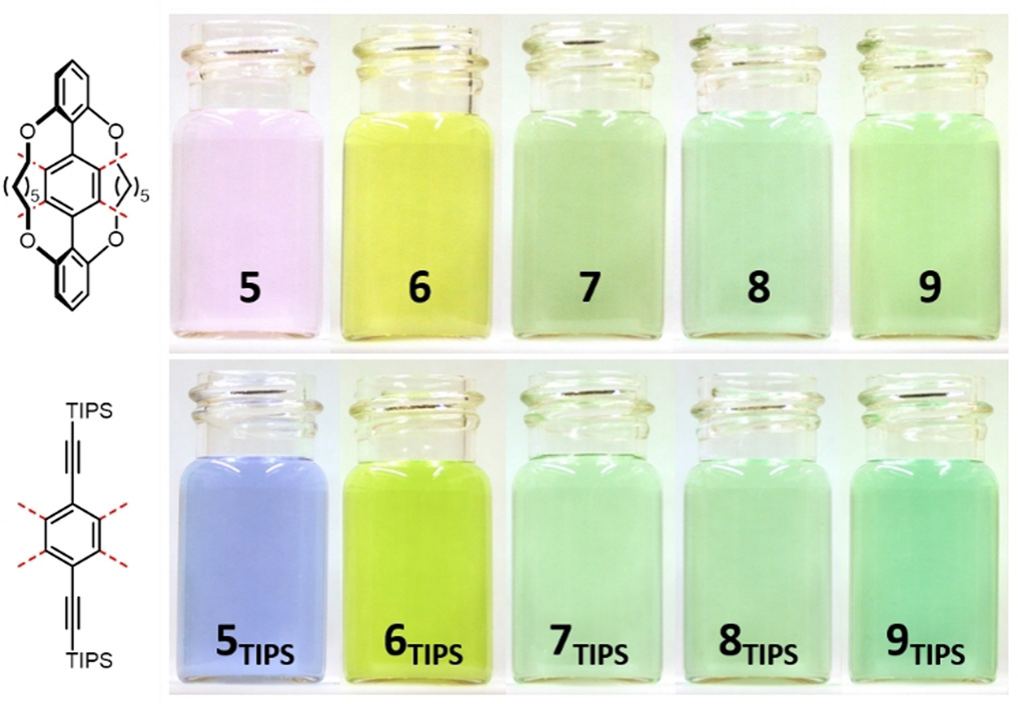
Photographs of doubly alkylene bridged (aza)pentacenes **5**–**9** (top, from left to right) and their respective TIPS‐ethynyl‐substituted analogues **5_TIPS_**‐**9_TIPS_** (bottom, from left to right) under daylight in *n*‐hexane.

**Figure 2:**
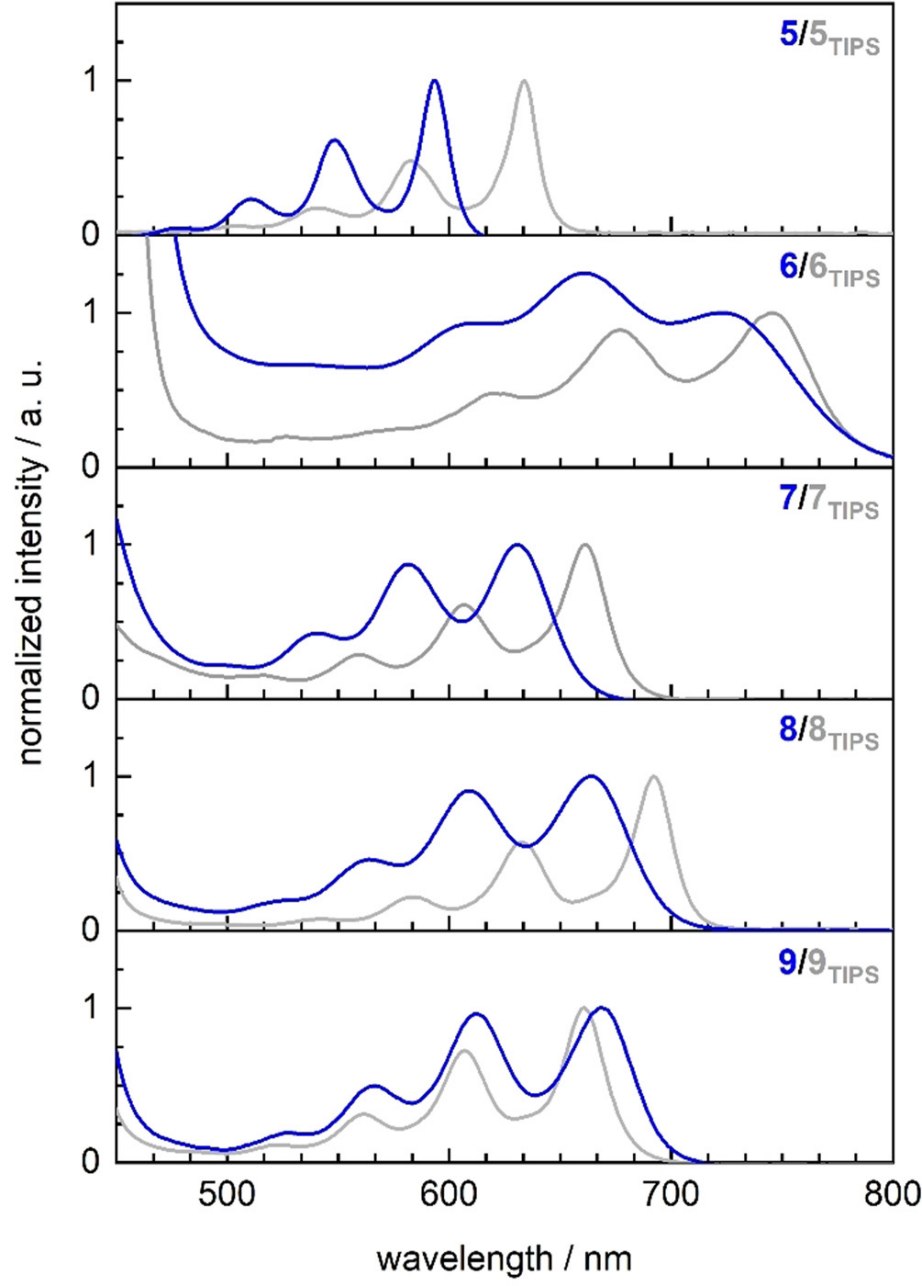
Normalized absorption spectra of doubly alkylene bridged (aza)pentacenes **5**–**9** and their consanguine silylated counterparts **5_TIPS_‐9_TIPS_** in dilute *n*‐hexane solution. See SI for full spectra.

Table 1 displays the electronic properties of the targets and their first reduction potentials. As expected, tetraaza‐derivative **6** is most easily reduced, while the other azaacenes display fairly similar reduction potentials (−1.4 to −1.5 V, vs. Fc/Fc^+^) and electron affinities. This trend is echoed in the silylethynylated (aza)acenes, which are more readily reduced due to the electronegative ethynyl substituents with reduction potentials ranging between −1.0 to −1.2 V for the diaza‐derivatives.

**Table 1:** Experimental and calculated (gas phase) properties of doubly alkylene bridged pentacenes **5**–**9** in solution (UV‐vis: *n*‐hexane; CV: DCM). For electrochemical and optical data of their consanguine TIPS‐counterparts **5_TIPS_**‐**9_TIPS_** see SI.

Compd	*λ*_max, abs_ [nm]	*λ*_max, em_ [nm]	*E* ^(0/−)^ [V]^[a]^	Ionization Potential/ HOMO [eV] ^[c]^meas./^[d]^calcd	Electron Affinity/ LUMO [eV] ^[b]^meas./^[d]^calcd
**5**	593	599	−1.83	−5.30/−4.74	−3.27/−2.63
**6**	721	–	−0.91	−5.79/−5.53	−4.19/−3.48
**7**	631	652	−1.40	−5.57/−5.28	−3.70/−3.17
**8**	663	687	−1.51	−5.33/−4.94	−3.59/−2.89
**9**	668	685	−1.43	−5.44/−5.02	−3.67/−2.97

[a] First reduction potentials from cyclic voltammetry (CV) in DCM at room temperature with Bu_4_NPF_6_ as the electrolyte against Fc/Fc^+^ as an internal standard (−5.10 eV) at 0.2 V s^−1^;^[13]^ [b] electron affinity_meas._=−e×(5.1 V+*E* ^(o/−)^); [c] ionization potential_meas._=electron affinity_meas_−gap_meas_; [d] obtained from DFT calculations (Gaussian16 B3LYP/ def2‐SVP// Gaussian16 B3LYP/ def2‐TZVP; TMS groups were used instead of TIPS).^[14]^

Single crystalline specimen of **5**, **6** and **9** were obtained by slow diffusion of methanol into a chloroform solution of the (aza)acene (Figure 3). Bond lengths and angles of the aromatic cores are in agreement with calculated values. Both **6** and **9** contain chloroform in the crystal lattice. The packing is dominated by van der Waals contacts of the bridging rings with each other. π‐π‐contacts are not observed for the (aza)pentacenes (packing diagram see SI), as the double bridges dominate the supramolecular structure.

**Figure 3:**
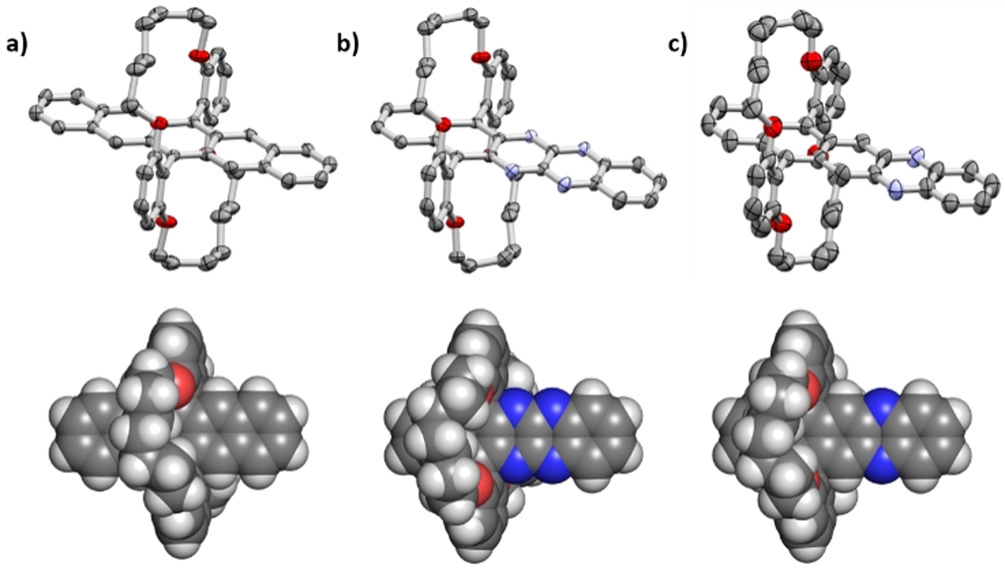
Solid state structures of (aza)pentacenes **5** (a), **6** (b) and **9** (c). Top: Thermal ellipsoids set to 50 % probability level. Bottom: Top‐view of space‐filling models.

Important is the relative stability **5**–**9** under irradiation (Figure 4), performed under air and under argon (10^−5^ mol L^−1^, DCM, ambient temperature).

**Figure 4:**
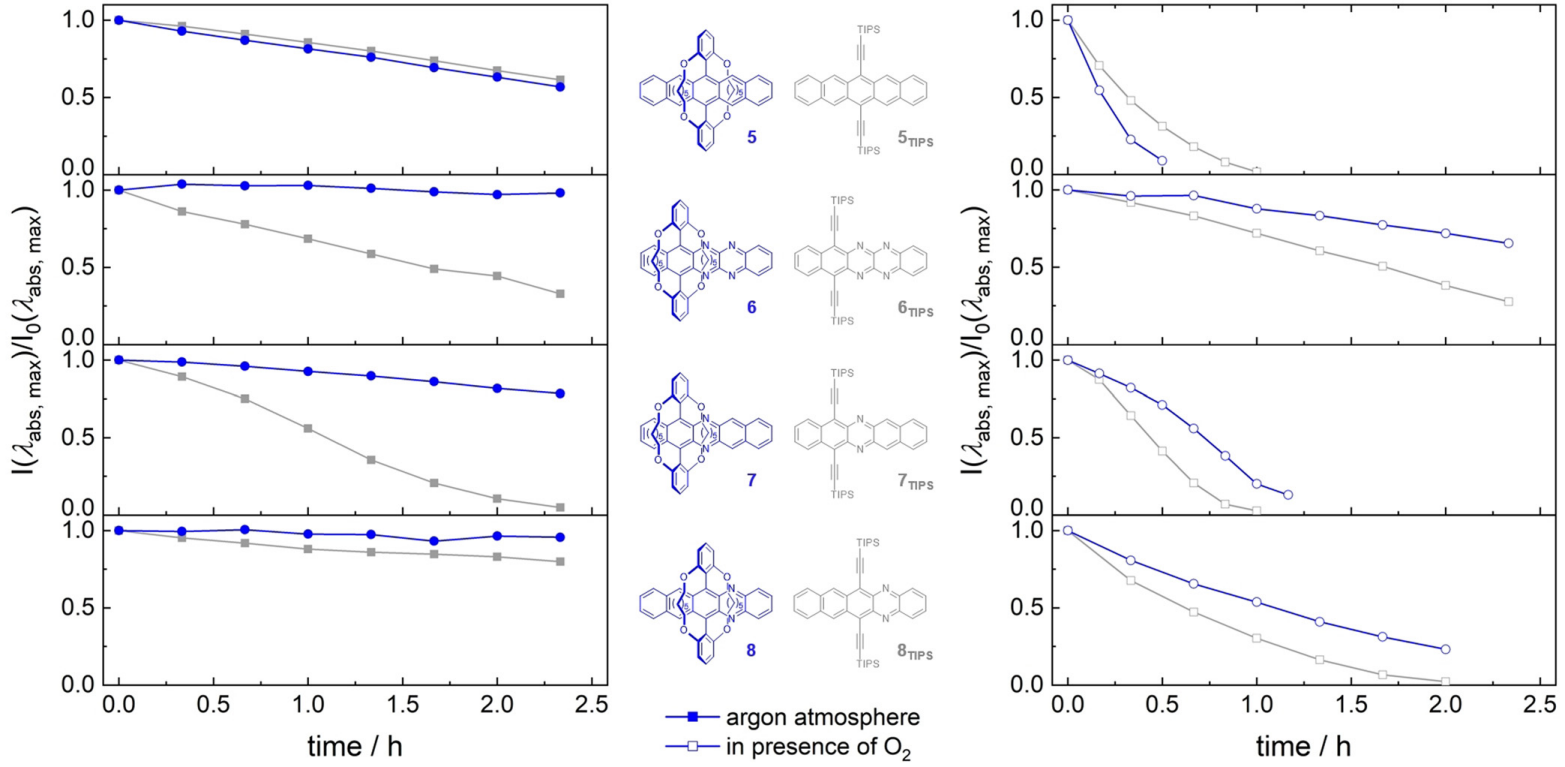
Time‐dependent evolution of UV/vis absorption intensities at *λ*_abs, max_ for **5**–**8** and **5_TIPS_‐8_TIPS_** (10^−5^ mol L^−1^) after irradiation with a handheld UV lamp (*λ*_1_=365 nm and *λ*_2_=254 nm) in *n*‐hexane at room temperature under argon atmosphere (left) and under ambient conditions (right). Distance to lamp was 5 cm for measurements under argon atmosphere and 20 cm under ambient conditions. **8** and **8_TIPS_** show almost identical decomposition rates compared to **9** and **9_TIPS_** (see SI, Figure S54), hence position of the sterically demanding group has less impact on stability compared to the position of nitrogen substitution.

Under argon, **5** is of comparable stability to **5_TIPS_**, but in air, **5** is more easily oxidized than **5_TIPS_** (Figure 4). We isolated **10**, a rearrangement product of the *endo*‐peroxide of **5**, identified under mass spectrometric conditions and by its single crystal structure (Figure 5 a). This rearrangement was previously described by Rigaudy et al. in the photolytic decomposition of anthracene.^[15]^ We propose predominant formation of an 5,14‐*endo*‐peroxide for **5** due to steric shielding, whereas for **5_TIPS_** the main product is the 6,13‐*endo*‐peroxide (98:2; 6,13‐ vs. 5,14‐*endo*‐peroxide).^[16]^

**Figure 5:**
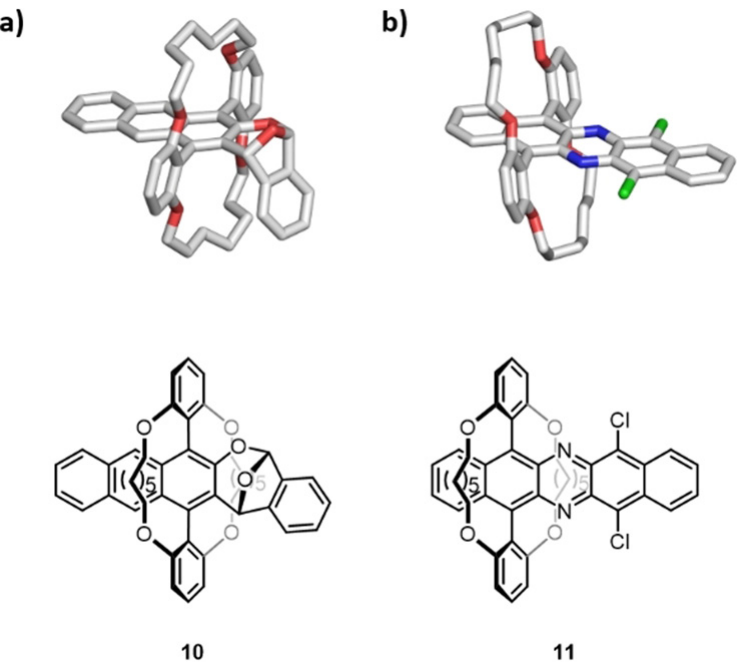
Identified products of photolytic decomposition reactions: Solid state structures of a) **10** produced in photolysis of **5** under air and b) decomposition product **11** formed from **7**.

**6**–**9** are consistently more stable than their TIPS‐ethynyl‐congeners, both under argon but also under air. We note that the position of the pyrazine unit and to a lesser effect the position of the substituents influence the reactivity for the TIPS‐ethynyl substituted azaacenes. The bridged azaacenes **6**, **8** and **9** were still intact after 18 h irradiation under argon atmosphere. Irradiation in DCM under ambient conditions chlorinates the azaacenes, as verified by mass spectrometry. **7** furnishes **11** as one of the photoproducts (Figure 5 b) we could isolate. Generally, the mixtures formed during the photolysis of the azaacenes are difficult to separate and to characterize.

To expand the clipping‐and‐jacketing concept, we reacted **12** (Pd‐catalyzed) with 2,3‐dichloroquinoxaline, treated the coupling‐product with PbO_2_ and obtained the tetraazahexacene **13** in 53 % yield (Scheme 2, *λ*_max abs_=946 nm).^[12]^ An X‐ray analysis proves the topology; **13** crystallizes without solvent and displays π‐π‐overlap involving the electron rich and electron poor parts of the hexacene, respectively (Figure 6).^[18]^ **13** is stable and can be handled without any problem, demonstrating the use of jacketing.

**Figure 6:**
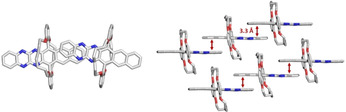
Packing of molecules of **13** in the single crystal.^[18]^

**Scheme 2:**
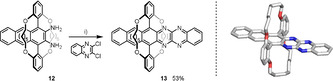
Synthesis of doubly alkylene bridged azahexacene **13**. Conditions: i) 1.) Cs_2_CO_3_, RuPhos Pd G1 (10 mol %), toluene, 110 °C, 15 h; 2.) PbO_2_, DCM, 0 °C, 30 min.

In large azaacenes, Kobayashi's double alkylene bridging, termed “clipping‐and‐jacketing”, is superior to TIPS‐alkyne substitutens with respect to stabilization. Tetraazahexacene **13** packs in the single crystalline state with π‐ π‐stacking; it has not escaped our attention that molecules like **13** might be useful as n‐channel semiconductors in thin‐film transistors. Jacketing could emerge as powerful alternative to trialkylsilylalkynylation, particularly as nature and steric demand of the alkylene bridges—the jackets—are easily varied.

## Conflict of interest

The authors declare no conflict of interest.

## Supporting information

As a service to our authors and readers, this journal provides supporting information supplied by the authors. Such materials are peer reviewed and may be re‐organized for online delivery, but are not copy‐edited or typeset. Technical support issues arising from supporting information (other than missing files) should be addressed to the authors.

Supplementary
