# Supplementary material for: (Aza)Pentacenes Clipped into a Ring: Stabilization of Large (Aza)Acenes
Source: Angew Chem Int Ed Engl. 2021 Feb 3;60(17):9270–3. doi: 10.1002/anie.202015348 (PMC8247972; doi:10.1002/anie.202015348)
Supplement: Supplementary file 1 — Supplementary [file ANIE-60-9270-s001.pdf]

Angewandte Chemie
Eine Zeitschrift der Gesellschaft Deutscher Chemiker

Supporting Information

(Aza)Pentacenes Clipped into a Ring: Stabilization of Large (Aza)Acenes

Lukas Ahrens, Olena Tverskoy, Svenja Weigold, Michael Ganschow, Frank Rominger, Jan Freudenberg, and Uwe H. F. Bunz**

anie_202015348_sm_miscellaneous_information.pdf

Table of Contents

1 Experimental Procedures 2
1.1 Materials and Methods 2
1.2 Synthesis 2
1.2.1 General Procedures 2
1.2.2 Precursor Synthesis 3
1.2.3 Synthesis of (Aza-)Pentacenes and Tetraazahexacene 14
1.2.4 Degradation Experiments 19
2 Results and Discussion 21
2.1 Calculations 21
2.2 NMR Spectroscopy 22
2.3 UV/vis Stability Studies 40
2.4 Cyclic Voltammetry 47
2.5 Crystallographic Data 51
References 65
Author Contributions 65

1 Experimental Procedures

1.1 Materials and Methods

Column chromatography was performed using silica gel from MACHEREY, NAGEL & CO. (particle size: 0.032−0.062 mm). NMR spectra were recorded at room temperature on BRUKER AVANCE Spectrometers using the specified frequency. Chemical shifts (δ) are given in parts per million (ppm) relative to internal solvent signals.[S1] The following abbreviations describe the signal multiplicities: s = singlet, bs = broad singlet, d = doublet, t = triplet, m = multiplet. IR spectra were recorded from neat oil or powder of the respective analyte on a JASCO FT/IR-4100 spectrometer. High-resolution mass spectra (HRMS) were obtained by (matrix-assisted) laser desorption/ionization (LDI/MALDI) using *trans*-2-[3-(4-*tert*-butylphenyl)-2-methyl-2-propenylidene]malononitrile (DCTB) as matrix, electrospray ionisation (ESI) or direct analysis in real time (DART) experiments on a BRUKER ApexQe hybrid 9.4 T FT-ICR spectrometer or BRUKER Autoflex Speed TOF spectrometer. CV measurements were performed on a Autolab PGSTAT101 potentiostat/galvanostat by METROHM AG. UV-vis spectra were recorded on a JASCO V670 (absorption) and a JASCO FP-6500 (fluorescence). Computational studies were carried out using DFT calculations on Gaussian 16. TMS groups were used instead of TIPS groups to simplify calculations. First, the gas-phase ground-state equilibrium geometry of the molecules was optimized at the B3LYP/def2-SVP level of theory. Afterwards, the recieved geometries were refined using the B3LYP/def2-TZVP level of theory. FMO calculations were performed starting from the optimized geometries on the B3LYP/def2-TZVP level of theory.[S2] 6,13-Dichloropentacene (**1**),[S3] naphtho[2,3-*c*][1,2,5]thiadiazole-4,9-dione (**S1**),[S4] anthra[2,3-*c*][1,2,5]thiadiazole-4,11-dione (**S2**),[S5] 2,3-dibromoanthracene-9,10-dione (**S9**),[S6] $\mathbf{5_{TIPS}}$-$\mathbf{9_{TIPS}}$[S7] and $\mathbf{13_{TIPS}}$[S8] were synthesized according to literature procedures.

1.2 Synthesis

1.2.1 General Procedures

GP1: Addition of arylmagnesium bromides to para-quinones

In a heatgun dried Schlenk tube under an atmosphere of argon was dissolved 1,3-dimethoxybenzene (5.05 equiv., 500 mmol L^{-1}) in anhydrous tetrahydrofuran. *n*-Butyllithium (2.5 mol L^{-1} in hexanes, 5.00 equiv.) was added dropwise at -78 °C, the reaction mixture was stirred at room temperature for 3 h. In a second heatgun dried Schlenk tube under an atmosphere of argon were suspended freshly ground magnesium turnings (7.60 equiv., 500 mmol L^{-1}) in anhydrous tetrahydrofuran. 1,2-Dibromoethane (7.60 equiv.) was added slowly and the reaction mixture was stirred at 60 °C for 1 h until the magnesium had disappeared. Then the prepared (2,6-dimethoxyphenyl)lithium solution was slowly added at -78 °C, followed by the respective quinone compound (1.00 equiv) at 0 °C. The resulting reaction mixture was stirred at room temperature for 16 h. It was then diluted with water (20 mL) and thereafter with aqueous hydrochloric acid (1 mol L^{-1}, 40 mL) at 0 °C. If not noted otherwise, the precipitate was separated by filtration, washed with water (100 mL) and ice-cold methanol (50 mL) and the addition product used without further purification.

GP2: Reductive aromatization with sodium hypophosphite

In a heatgun dried Schlenk tube under an atmosphere of argon were added the diol (1.00 equiv. 30 mmol L^{-1}), sodium iodide (7.00 equiv.) and sodium hypophosphite hydrate (10.0 equiv.). Thereafter acetic acid was added and the reaction mixture stirred at 120 °C for 4 - 6 h. The precipitate was separated by filtration, washed with water (50 mL) and cold methanol (30 mL) to yield the product.

GP3: Deprotection of methyl ethers with boron tribromide

In a heatgun dried Schlenk tube under an atmosphere of argon was dissolved the methyl ether (1.00 equiv., 10 mmol L^{-1}) in anhydrous dichloromethane. Then a solution of boron tribromide (8.00 equiv.) in anhydrous dichloromethane was added dropwise at -78 °C and

the reaction mixture stirred at room temperature for 48 h up to several days. The mixture was poured into ice-cold water and neutralized with sodium bicarbonate solution. Most of the dichloromethane was removed *in vacuo* before the mixture was extracted with ethyl acetate (3×30 mL). The combined organic phases were washed with water (10 mL) and brine (10 mL), dried over magnesium sulfate and filtrated. The solvent was removed under reduced pressure and after reprecipitation from THF-hexane the product was isolated.

GP4: Introduction of double alkylene-tethers in substitution reaction

In a heatgun dried Schlenk tube under an atmosphere of argon was suspended the tetraol (1.00 equiv., 5 mmol L^{-1}) in anhydrous dimethylformamide. Potassium carbonate (5.00 equiv.) and 1,7-dibromoheptane (2.10 equiv.) were added and the reaction mixture stirred at 40 °C for 24 h and then at 80 °C for 48 h. The solvent was removed under reduced pressure and the residue redissolved in 50 mL dichloromethane and 50 mL water. The mixture was extracted with dichloromethane (3×30 mL). The combined organic phases were washed with water (10 mL) and brine (10 mL), dried over magnesium sulfate and filtrated. The solvent was removed under reduced pressure and the crude product was absorbed on Celite®. After flash column chromatography the product was isolated.

GP5: Buchwald-Hartwig amination in toluene

In a heatgun dried Schlenk tube under an atmosphere of argon was added the aryl halide (1.00 equiv., 50 mmol L^{-1}), the *ortho*-diamine (1.00 – 1.50 equiv.), caesium carbonate (3.00 equiv.) and RuPhos Pd G1 (10 mol%). Then anhydrous, degassed toluene was added, and the reaction mixture stirred at 110 °C for 24 h. The mixture was cooled to room temperature and diluted with water (10 mL). The phases were separated, and the aqueous layer was extracted with dichloromethane (3×10 mL). The combined organic phases were washed with brine (10 mL), dried over magnesium sulfate and filtrated. The solvent was removed under reduced pressure and the crude product was absorbed on Celite®. After flash column chromatography and gel permeation chromatography (toluene) the coupling product was isolated.

1.2.2 Precursor Synthesis

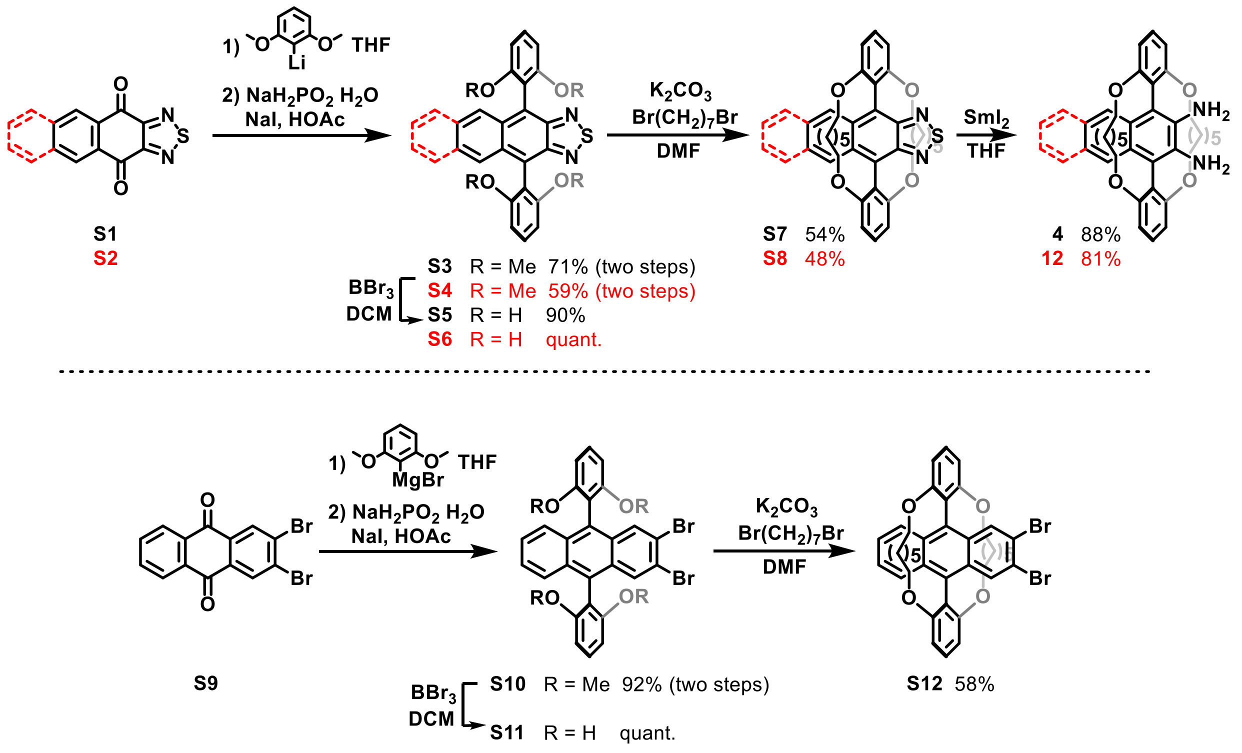

Scheme S1. Synthesis of of precursor molecules **4, 12** and **S12**.

4,9-Bis(2,6-dimethoxyphenyl)-4,9-dihydronaphtho[2,3-*c*][1,2,5]thiadiazole-4,9-diol (**S13**)

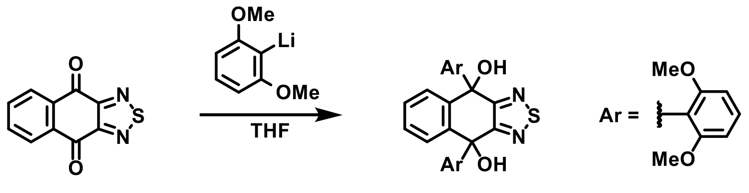

In a heatgun dried Schlenk tube under an atmosphere of argon was dissolved 1,3-dimethoxybenzene (6.06 mL, 6.39 g, 46.3 mmol, 5.00 equiv.) in 80.0 mL anhydrous tetrahydrofuran. *n*-Butyllithium (2.5 mol L^{-1} in hexanes, 18.5 mL, 46.3 mmol, 5.00 equiv.) was added dropwise at -78 °C, the reaction mixture was stirred at -78 °C for 10 min and thereafter at room temperature for 4 h. In a second heatgun dried Schlenk tube under an atmosphere of argon was suspended **S1** (2.00 g, 9.25 mmol, 1.00 equiv.) in 40.0 mL anhydrous tetrahydrofuran and then the previously made (2,6-dimethoxyphenyl)lithium solution was slowly added at -78 °C. The resulting reaction mixture was stirred at room temperature for 15 h and then diluted with water (20 mL) and thereafter with aqueous hydrochloric acid (1 mol L^{-1}, 40 mL) at 0 °C. The precipitate was separated by filtration, washed with water (100 mL) and ice-cold methanol (50 mL) and used without further purification. The product **S13** was isolated as colorless solid (4.16 g, 8.44 mmol, 91%).

HRMS (DART$^+$) m/z: $[M-H]^{\cdot+}$: calcd. for $[C_{26}H_{23}N_2O_6S]^{\cdot+}$: 491.1271; found 491.1278; correct isotope distribution.

4,9-Bis(2,6-dimethoxyphenyl)naphtho[2,3-*c*][1,2,5]thiadiazole (**S3**)

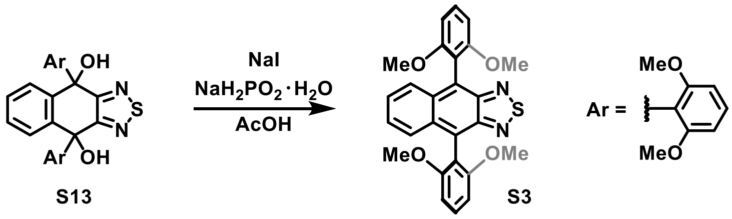

GP2 was applied to **S13** (1.00 g, 2.03 mmol, 1.00 equiv.), sodium iodide (2.13 g, 14.2 mmol, 7.00 equiv.), and sodium hypophosphite hydrate (2.15 g, 20.3 mmol, 10.0 equiv.) in 30.0 mL acetic acid. The reaction mixture stirred at 120 °C for 5 h. The product **S3** was isolated as an orange solid (724 mg, 1.58 mmol, 78%).

R*f* = 0.31 (SiO_2; petroleum ether/ethyl acetate 2:1, v/v).

Mp: 283 - 285 °C.

^{1}H NMR ($CDCl_3$, 400 MHz, rt): δ = 7.63 - 7.70 (m, 2H), 7.51 (t, *J* = 8.34 Hz, 2H), 7.21 - 7.26 (m, 2H), 6.83 (d, *J* = 8.34 Hz, 4H), 3.63 (s, 12H) ppm.

^{13}C {^{1}H} NMR ($CDCl_3$, 101 MHz, rt): δ = 158.9, 152.1, 133.1, 130.4, 127.5, 125.7, 123.9, 114.2, 104.7, 56.2 ppm.

IR (ATR): $\tilde{\nu}$ = 2934, 2835, 1586, 1469, 1431, 1248, 1099, 893, 774, 724, 616 cm^{-1}.

UV-Vis (*n*-hexane, rt): $\lambda_{max,\ abs}$ = 485 nm, $\lambda_{max,\ em}$ = 527 nm, Φ = 0.45.

HRMS (MALDI$^+$) m/z: $[M]^{\cdot+}$: calcd. for $[C_{26}H_{22}N_2O_4S]^{\cdot+}$: 458.1295; found 458.1293; correct isotope distribution.

Crystal data

Single crystalline specimen were obtained by slow diffusion of methanol into a chloroform solution of **S3**:

Orange crystal (plank), dimensions 0.090 x 0.040 x 0.028 mm^3, crystal system monoclinic, space group P2$_1$/c, Z = 6, a = 20.5671(9) Å, b = 7.5774(2) Å, c = 20.8300(9) Å, α = 90 °, β = 92.846(4) °, γ = 90 °, V = 3242.2(2) Å^3, ρ = 1.409 g/cm^3, T = 200(2) K, Θ_{max} = 72.034 °, 21067 reflections measured, 6130 unique (R_{int} = 0.0647), 3433 observed ($I > 2\sigma(I)$), μ = 1.64 mm^{-1}, T_{min} = 0.69, T_{max} = 1.48, 551 parameters refined, hydrogen atoms were treated using appropriate riding models, goodness of fit 1.04 for observed reflections, final residual values R1(F) = 0.060, wR(F^2) = 0.103 for observed reflections, residual electron density -0.33 to 0.29 eÅ^{-3}.

2,2'-(Naphtho[2,3-*c*][1,2,5]thiadiazole-4,9-diyl)di(benzene-1,3-diol) (**S5**)

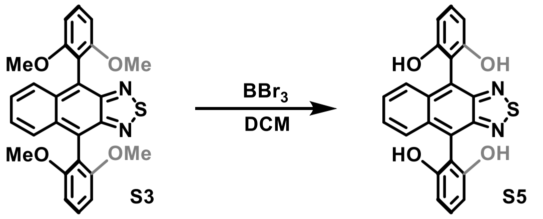

GP3 was applied to **S3** (680 mg, 1.48 mmol, 1.00 equiv.) and boron tribromide (1.13 mL, 2.97 g, 11.9 mmol, 8.00 equiv.) in 100 mL anhydrous dichloromethane and the reaction mixture stirred at 40 °C for 48 h. Reprecipitation (THF-hexane) yielded **S5** as an orange solid (537 mg, 1.33 mmol, 90%).

R$_f$ = 0.20 (SiO_2; petroleum ether/ethyl acetate 1:1, v/v).

Mp: 207 - 210 °C.

^{1}H NMR (THF-d$_8$, 400 MHz, rt): δ = 7.77 – 7.82 (m, 2H), 7.40 (s, 4H), 7.21 - 7.26 (m, 2H), 7.13 (t, J = 8.16 Hz, 2H), 6.48 (d, J = 8.16 Hz, 4H) ppm.

^{13}C {^{1}H} NMR (THF-d$_8$, 101 MHz, rt): δ = 157.7, 153.8, 134.7, 130.2, 128.4, 126.3, 124.9, 112.1, 107.8 ppm.

IR (ATR): $\tilde{\nu}$ = 3312, 2928, 1616, 1459, 1259, 1173, 1146, 1005, 897, 782, 760, 729, 402 cm^{-1}.

HRMS (ESI$^-$) m/z: [M-H]$^{\cdot-}$: calcd. for $[C_{22}H_{13}N_2O_4S]^{\cdot-}$: 401.0602; found 401.0597; correct isotope distribution.

Doubly alkylene-bridged naphtho[2,3-*c*][1,2,5]thiadiazole **S7**

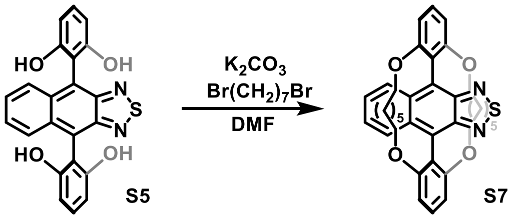

GP4 was applied to **S5** (250 mg, 621 µmol, 1.00 equiv.), potassium carbonate (429 mg, 3.11 mmol, 5.00 equiv.) and 1,7-dibromoheptane (223 µL, 337 mg, 1.30 mmol, 2.10 equiv.) in 120 mL anhydrous dimethylformamide. Flash column chromatography (SiO_2; petroleum ether/dichloromethane 5:1 v/v -> 2:1) yielded **S7** as an orange solid (199 mg, 334 µmol, 54%).

R*f* = 0.56 (SiO_2; petroleum ether/ethyl acetate 4:1, v/v).

Mp: ≥350 °C.

^{1}H NMR ($CDCl_3$, 600 MHz, rt): δ = 7.69 - 7.72 (m, 2H), 7.46 (t, J = 8.32 Hz, 2H), 7.24 - 7.27 (m, 2H), 6.83 (d, J = 8.32 Hz, 4H), 3.75 - 3.86 (m, 8H), 1.08 - 1.21 (m, 8H), 0.61 - 0.69 (m, 4H), 0.36 - 0.49 (m, 8H) ppm.

^{13}C {^{1}H} NMR ($CDCl_3$, 151 MHz, rt): δ = 158.7, 151.9, 133.0, 130.3, 127.6, 125.7, 124.3, 117.1, 107.9, 69.7, 29.3, 28.0, 25.2 ppm.

IR (ATR): $\tilde{\nu}$ = 2926, 2857, 1583, 1454, 1381, 1240, 1109, 1089, 892, 850, 755, 724, 677, 615 cm^{-1}.

UV-Vis (*n*-hexane, rt): $\lambda_{max,\ abs}$ = 488 nm, $\lambda_{max,\ em}$ = 529 nm, Φ = 0.48.

HRMS (ESI^+) m/z: $[M+H]^{\cdot+}$: calcd. for $[C_{36}H_{39}N_2O_4S]^{\cdot+}$: 595.2625; found 595.2629; correct isotope distribution.

Crystal data

Single crystalline specimen were obtained by slow diffusion of methanol into a chloroform solution of **S7**:

Orange crystal (plank), dimensions 0.095 x 0.076 x 0.022 mm^3, crystal system monoclinic, space group $P2_1$, Z = 2, a = 8.3208(3) Å, b = 18.4339(10) Å, c = 9.9957(4) Å, α = 90 °, β = 99.993(3) °, γ = 90 °, V = 1509.93(12) $Å^3$, ρ = 1.308 g/cm^3, T = 200(2) K, Θ_{max} = 67.167 °, 9474 reflections measured, 4356 unique (R_{int} = 0.0471), 2827 observed ($I > 2\sigma(I)$), μ = 1.30 mm^{-1}, T_{min} = 0.69, T_{max} = 1.49, 453 parameters refined, hydrogen atoms were treated using appropriate riding models, Flack absolute structure parameter 0.41(5), goodness of fit 0.99 for observed reflections, final residual values R1(F) = 0.049, wR(F^2) = 0.094 for observed reflections, residual electron density -0.19 to 0.31 $eÅ^{-3}$.

Doubly alkylene-bridged naphthalene-2,3-diamine **4**

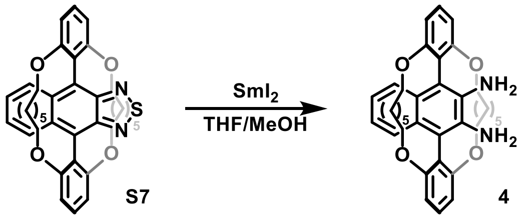

In a Schlenk tube under an atmosphere of argon was dissolved **S7** (100 mg, 168 µmol, 1.00 equiv.) in 8.20 mL anhydrous tetrahydrofuran and 200 µL anhydrous methanol. Samarium(II) iodide (100 mmol L^{-1} in tetrahydrofuran, 20.2 mL, 2.02 mmol, 12.0 equiv.) was added dropwise at -10 °C and the reaction mixture was stirred at room temperature for 2 h. The suspension was diluted with water (20 mL) and extracted with dichloromethane (3×20 mL). The combined organic phases were washed with aqueous sodium thiosulfate (10 mL), brine (10 mL), dried over magnesium sulfate and filtrated. The solvent was removed under reduced pressure and the crude product was absorbed on Celite®. After flash column chromatography (petroleum ether/ethyl acetate 5:1 v/v) the product **4** was isolated as a colorless solid (84.1 mg, 148 µmol, 88%).

R$_f$ = 0.47 (SiO_2; petroleum ether/ethyl acetate 2:1, v/v).

Mp: 277 - 281 °C.

^{1}H NMR ($CDCl_3$, 400 MHz, rt): δ = 7.37 (t, J = 8.19 Hz, 2H), 7.09 - 7.15 (m, 2H), 6.99 - 7.05 (m, 2H), 6.79 (d, J = 8.19 Hz, 4H), 3.81 - 3.90 (m, 4H), 3.68 - 3.76 (m, 4H), 3.41 (bs, 4H), 1.19 - 1.43 (m, 8H), 0.78 - 0.91 (m, 12H) ppm.

^{13}C{^{1}H} NMR ($CDCl_3$, 101 MHz, rt): δ = 159.2, 133.9, 129.6, 128.9, 124.5, 122.1, 117.8, 115.3, 109.0, 70.4, 29.2, 28.1, 25.1 ppm.

IR (ATR): $\tilde{\nu}$ = 2929, 2860, 1575, 1452, 1428, 1238, 1088, 754, 723, 630 cm^{-1}.

HRMS (ESI^+) m/z: $[M+H]^{\cdot+}$: calcd. for $[C_{36}H_{43}N_2O_4]^{\cdot+}$: 567.3217; found 567.3228; correct isotope distribution.

4,11-Bis(2,6-dimethoxyphenyl)-4,11-dihydroanthra[2,3-*c*][1,2,5]thiadiazole-4,11-diol (**S14**)

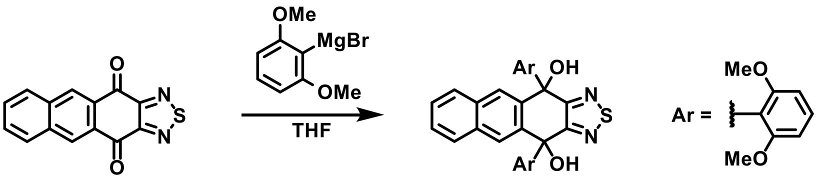

GP1 was applied to (2,6-dimethoxyphenyl)magnesium bromide, prepared from 1,3-dimethoxybenzene (4.99 mL, 5.24 g, 37.9 mmol, 5.05 equiv.), *n*-butyllithium (2.5 mol L^{-1} in hexanes, 15.0 mL, 2.41 g, 37.6 mmol, 5.00 equiv.) in 100 mL anhydrous tetrahydrofuran and magnesium (1.39 g, 57.1 mmol, 7.60 equiv.), 1,2-dibromoethane (4.92 mL, 10.7 g, 57.1 mmol, 7.60 equiv.) in 100 mL anhydrous tetrahydrofuran, and quinone **S2** (2.00 g, 7.51 mmol, 1.00 equiv.). The product **S14** was isolated as colorless solid (2.93 g, 5.39 mmol, 72%) and used without further purification.

HRMS (ESI^+) m/z: $[M]^{\cdot+}$: calcd. for $[C_{30}H_{26}N_2NaO_6S]^{\cdot+}$: 565.1404; found 565.1412; correct isotope distribution.

4,11-Bis(2,6-dimethoxyphenyl)anthra[2,3-*c*][1,2,5]thiadiazole (**S4**)

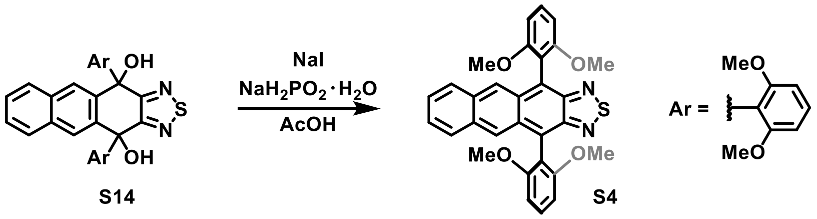

GP2 was applied to **S14** (2.90 g, 5.34mmol, 1.00 equiv.), sodium iodide (5.61 g, 37.4 mmol, 7.00 equiv.), and sodium hypophosphite hydrate (5.66 g, 53.5 mmol, 10.0 equiv.) in 150 mL acetic acid and the reaction mixture stirred at 120 °C for 4 h. **S4** was isolated as a purple solid (2.45 g, 4.82 mmol, 90%).

R$_f$ = 0.23 (SiO_2; petroleum ether/ethyl acetate 2:1, v/v).

Mp: 343 - 346 °C.

^{1}H NMR ($CDCl_3$, 600 MHz, rt): δ = 8.30 (s, 2H), 7.68 - 7.72 (m, 2H), 7.58 (t, J = 8.48 Hz, 2H), 7.16 - 7.20 (m, 2H), 6.89 (d, J = 8.48 Hz, 4H), 3.62 (s, 12H) ppm.

^{13}C {^{1}H} NMR ($CDCl_3$, 151 MHz, rt): δ = 159.1, 151.2, 132.1, 131.5, 130.5, 128.7, 126.1, 125.4, 123.7, 114.5, 104.7, 56.2 ppm.

IR (ATR): $\tilde{\nu}$ = 2956, 2924, 2889, 2829, 1594, 1582, 1430, 1079, 1025, 939, 872, 779, 760, 726, 717, 635, 624, 459 cm^{-1}.

UV-Vis (*n*-hexane, rt): $\lambda_{max, abs}$ = 612 nm, $\lambda_{max, em}$ = 631 nm, Φ = 0.19.

HRMS (DART$^-$) m/z: [M]$^{\cdot-}$: calcd. for $[C_{30}H_{24}N_2O_4S]^{\cdot-}$: 508.1462; found 508.1461; correct isotope distribution.

Crystal data

Single crystalline specimen were obtained by slow diffusion of methanol into a chloroform solution of **S4**:

Violet crystal (needle), dimensions 0.178 x 0.015 x 0.014 mm^3, crystal system orthorhombic, space group Pbcn, Z = 4, a = 19.055(2) Å, b = 16.1660(12) Å, c = 7.7352(6) Å, α = 90 °, β = 90 °, γ = 90 °, V = 2382.8(4) Å^3, ρ = 1.418 g/cm^3, T = 200(2) K, Θ_{max} = 69.176 °, 8919 reflections measured, 2191 unique (R_{int} = 0.1461), 984 observed ($I > 2\sigma(I)$), μ = 1.55 mm^{-1}, T_{min} = 0.39, T_{max} = 0.98, 170 parameters refined, hydrogen atoms were treated using appropriate riding models, goodness of fit 0.90 for observed reflections, final residual values R1(F) = 0.048, wR(F^2) = 0.073 for observed reflections, residual electron density -0.28 to 0.27 eÅ^{-3}.

2,2'-(Anthra[2,3-*c*][1,2,5]thiadiazole-4,11-diyl)di(benzene-1,3-diol) (**S6**)

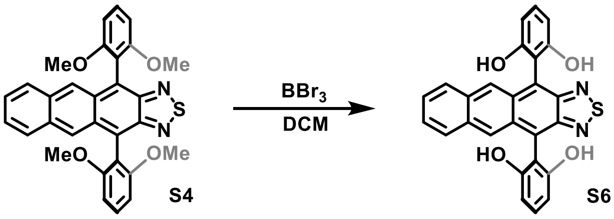

GP3 was applied to **S4** (450 mg, 885 µmol, 1.00 equiv.) and boron tribromide (672 µL, 1.77 g, 7.08 mmol, 8.00 equiv.) in 80 mL anhydrous dichloromethane. The reaction mixture was stirred at room temperature for 3 d. Reprecipitation (THF-hexane) yielded **S6** as a blue solid (398 mg, 878 µmol, 99%).

R$_f$ = 0.06 (SiO_2; petroleum ether/ethyl acetate 2:1, v/v).

Mp: 273 - 278 °C.

^{1}H NMR (THF-d$_8$, 600 MHz, rt): δ = 8.48 (s, 2H), 7.73 - 7.76 (m, 2H), 7.48 (s, 2H), 7.19 (t, J = 8.20 Hz, 2H), 7.16 - 7.19 (m, 2H), 6.54 (d, J = 8.20 Hz, 4H) ppm.

^{13}C {^{1}H} NMR (THF-d$_8$, 151 MHz, rt): δ = 156.9, 152.0, 132.0, 131.9, 129.3, 128.4, 126.0, 125.1, 123.7, 111.6, 106.9 ppm.

IR (ATR): $\tilde{\nu}$ = 3352, 2957, 2875, 1616, 1579, 1460, 1173, 1146, 1008, 876, 784, 741, 730 cm^{-1}.

HRMS (MALDI$^+$) m/z: [M]$^{\cdot+}$: calcd. for $[C_{26}H_{16}N_2O_4S]^{\cdot+}$: 452.0825; found 452.0833; correct isotope distribution.

Doubly alkylene-bridged anthra[2,3-*c*][1,2,5]thiadiazole **S8**

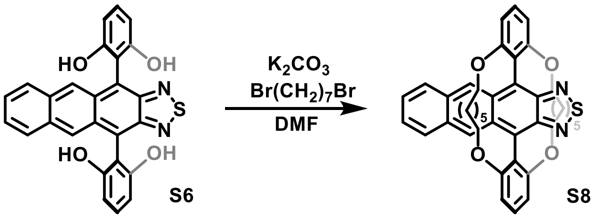

GP4 was applied to **S6** (200 mg, 442 µmol, 1.00 equiv.), potassium carbonate (305 mg, 2.21 mmol, 5.00 equiv.) and 1,7-dibromoheptane (159 µL, 239 mg, 928 µmol, 2.10 equiv.) in 120 mL anhydrous dimethylformamide. Flash column chromatography (SiO_2; petroleum ether/dichloromethane 10:1 v/v -> 2:1) and gel permeation chromatography (toluene) yielded **S8** as a dark blue solid (135 mg, 210 µmol, 48%).

R_f = 0.43 (SiO_2; petroleum ether/ethyl acetate 5:1, v/v).

Mp: 270 - 274 °C.

^{1}H NMR ($CDCl_3$, 600 MHz, rt): δ = 8.36 (s, 2H), 7.69 - 7.74 (m, 2H), 7.54 (t, *J* = 8.23 Hz, 2H), 7.17 - 7.21 (m, 2H), 6.89 (d, *J* = 8.23 Hz, 4H), 3.76 - 3.86 (m, 8H), 1.01 - 1.11 (m, 8H), 0.43 - 0.60 (m, 4H), 0.18 - 0.35 (m, 8H) ppm.

^{13}C {^{1}H} NMR ($CDCl_3$, 151 MHz, rt): δ = 158.9, 151.0, 132.0, 131.5, 130.4, 128.8, 126.3, 125.3, 124.2, 117.4, 108.0, 69.7, 29.2, 27.8, 25.1 ppm.

IR (ATR): $\tilde{\nu}$ = 2928, 2856, 1699, 1587, 1453, 1375, 1254, 1239, 1181, 1109, 1090, 766, 729, 665, 457 cm^{-1}.

UV-Vis (*n*-hexane, rt): $\lambda_{max, abs}$ = 619 nm, $\lambda_{max, em}$ = 638 nm, Φ = 0.33.

HRMS ($MALDI^+$) m/z: $[M]^{\cdot+}$: calcd. for $[C_{40}H_{40}N_2O_4S]^{\cdot+}$: 644.2703; found 644.2708; correct isotope distribution.

Crystal data

Single crystalline specimen were obtained by slow diffusion of methanol into a chloroform solution of **S8**:

Blue crystal (needle), dimensions 0.120 x 0.018 x 0.015 mm^3, crystal system tetragonal, space group $I\bar{4}$, Z = 8, a = 28.5703(19) Å, b = 28.5703(19) Å, c = 8.0905(10) Å, α = 90 °, β = 90 °, γ = 90 °, V = 6604.0(12) $Å^3$, ρ = 1.297 g/cm^3, T = 200(2) K, Θ_{max} = 51.869 °, 14708 reflections measured, 3584 unique (R_{int} = 0.1777), 1845 observed ($I > 2\sigma(I)$), μ = 1.23 mm^{-1}, T_{min} = 0.75, T_{max} = 1.40, 425 parameters refined, hydrogen atoms were treated using appropriate riding models, Flack absolute structure parameter 0.15(9), goodness of fit 1.01 for observed reflections, final residual values R1(F) = 0.077, $wR(F^2)$ = 0.150 for observed reflections, residual electron density -0.23 to 0.34 $eÅ^{-3}$.

Doubly alkylene-bridged anthracene-2,3-diamine **12**

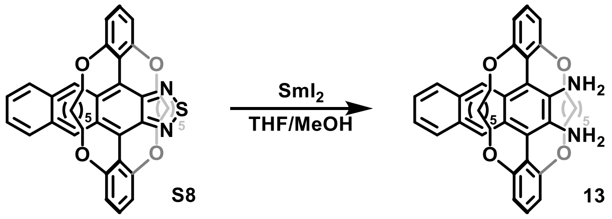

S8 13

In a Schlenk tube under an atmosphere of argon was dissolved **S8** (30.0 mg, 46.5 µmol, 1.00 equiv.) in 2.25 mL anhydrous tetrahydrofuran and 60.0 µL anhydrous methanol. Samarium(II) iodide (100 mmol L^{-1} in tetrahydrofuran, 5.58 mL, 226 mg, 558 µmol, 12.0 equiv.) was added dropwise at -10 °C and the reaction mixture was stirred at room temperature for 2 h. The suspension was diluted with water (10 mL) and extracted with dichloromethane (3×10 mL). The combined organic phases were washed with sodium thiosulfate solution (5 mL), brine (5 mL), dried over magnesium sulfate and filtrated. The solvent was removed under reduced pressure and the crude product was absorbed on Celite®. After flash column chromatography (petroleum ether/ethyl acetate 40:1 -> 20:1 -> 5:1 v/v) the product **12** was isolated as a pale-yellow solid (23.1 mg, 37.5 µmol, 81%).

R*f* = 0.45 (SiO_2; petroleum ether/ethyl acetate 2:1, v/v).

Mp: >298 °C (decomposition).

^{1}H NMR ($CDCl_3$, 400 MHz, rt): *δ* = 7.66 - 7.74 (m, 2H), 7.63 (s, 2H), 7.43 (t, *J* = 8.18 Hz, 2H), 7.13 - 7.21 (m, 2H), 6.85 (d, *J* = 8.18 Hz, 4H), 3.81 - 3.92 (m, 4H), 3.67 - 3.78 (m, 4H), 3.68 (bs, 4H), 1.41 - 1.07 (m, 8H), 0.61 - 0.92 (m, 12H) ppm.

^{13}C {^{1}H} NMR ($CDCl_3$, 101 MHz, rt): *δ* = 159.4, 134.8, 130.0, 129.7, 129.2, 128.0, 123.0, 121.8, 117.8, 113.6, 109.1, 70.4, 29.3, 28.0, 25.2 ppm.

IR (ATR): $\tilde{\nu}$ = 3341, 2926, 2855, 1573, 1452, 1436, 1239, 1085, 722, 594, 469 cm^{-1}.

HRMS ($MALDI^+$) m/z: $[M]^{\cdot+}$: calcd. for $[C_{40}H_{44}N_2O_4]^{\cdot+}$: 616.3296; found 616.3298; correct isotope distribution.

2,3-Dibromo-9,10-bis(2,6-dimethoxyphenyl)-9,10-dihydroanthracene-9,10-diol (**S15**)

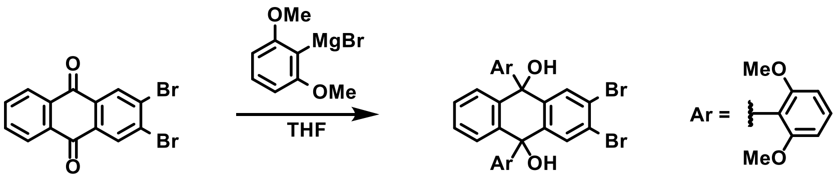

S9 S15

GP1 was applied to (2,6-dimethoxyphenyl)magnesium bromide, prepared from 1,3-dimethoxybenzene (5.45 mL, 5.72 g, 41.4 mmol, 5.05 equiv.), *n*-butyllithium (2.5 mol L^{-1} in hexanes, 16.4 mL, 2.63 g, 41.0 mmol, 5.00 equiv.) in 80 mL anhydrous tetrahydrofuran and magnesium (1.51 g, 62.3 mmol, 7.60 equiv.), 1,2-dibromoethane (5.37 mL, 11.7 g, 62.3 mmol, 7.60 equiv.) in 80 mL anhydrous tetrahydrofuran, as well as quinone **S9** (3.00 g, 8.20 mmol, 1.00 equiv.). **S15** was isolated as a colorless solid (4.47 g, 6.96 mmol, 85%) and used without further purification.

HRMS ($MALDI^+$) m/z: $[M\text{-}OH]^{\cdot+}$: calcd. for $[C_{30}H_{25}O5^{79}Br_2]^{\cdot+}$: 623.0063; found 623.0065; correct isotope distribution.

2,3-Dibromo-9,10-bis(2,6-dimethoxyphenyl)anthracene (**S10**)

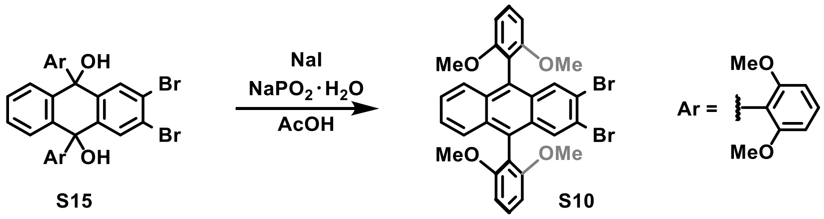

GP2 was applied to **S15** (4.30 g, 6.67 mmol, 1.00 equiv.), sodium iodide (7.00 g, 46.7 mmol, 7.00 equiv.), and sodium hypophosphite hydrate (7.07 g, 66.7 mmol, 10.0 equiv.) in 120 mL acetic acid. The reaction mixture stirred at 120 °C for 6 h. The product **S10** was isolated as a pale-yellow solid (3.73 g, 6.12 mmol, 92%).

R*f* = 0.69 (SiO_2; petroleum ether/ethyl acetate 2:1, v/v).

Mp: 314 - 315 °C.

^{1}H NMR ($CDCl_3$, 600 MHz, rt): δ = 7.90 (s, 2H), 7.55 - 7.58 (m, 2H), 7.52 (t, J = 8.36 Hz, 2H), 7.28 - 7.31 (m, 2H), 6.82 (d, J = 8.36 Hz, 4H), 3.60 (s, 12H) ppm.

^{13}C{^{1}H} NMR ($CDCl_3$, 151 MHz, rt): δ = 159.1, 131.4, 131.3, 130.2, 130.1, 129.9, 127.0, 125.6, 121.2, 114.8, 104.4, 56.1 ppm.

IR (ATR): $\tilde{\nu}$ = 2927, 2828, 1584, 1470, 1430, 1251, 1104, 1029, 944, 776, 763, 727, 679 cm^{-1}.

UV-Vis (*n*-hexane, rt): $\lambda_{max,\,abs}$ = 404 nm, $\lambda_{max,\,em}$ = 414 nm, Φ = 0.11.

HRMS ($MALDI^+$) m/z: $[M]^{\cdot+}$: calcd. for $[C_{30}H_{24}O_4{}^{79}Br_2]^{\cdot+}$: 606.0036; found 606.0045; correct isotope distribution.

Crystal data

Single crystalline specimen were obtained by slow diffusion of methanol into a chloroform solution of 2,3-dibromo-9,10-bis(2,6-dimethoxyphenyl)anthracene (**S10**):

Colorless crystal (plank), dimensions 0.290 x 0.067 x 0.029 mm^3, crystal system monoclinic, space group $P2_1/c$, Z = 4, a = 16.3942(10) Å, b = 7.8249(5) Å, c = 20.2080(12) Å, α = 90 °, β = 92.6999 (10) °, γ = 90 °, V = 2589.5(3) Å^3, ρ = 1.560 g/cm^3, T = 200(2) K, Θ_{max} = 30.098 °, 56361 reflections measured, 7600 unique (R_{int} = 0.0473), 5937 observed ($I > 2\sigma(I)$), μ = 3.16 mm^{-1}, T_{min} = 0.82, T_{max} = 0.93, 338 parameters refined, hydrogen atoms were treated using appropriate riding models, goodness of fit 1.05 for observed reflections, final residual values R1(F) = 0.037, wR(F^2) = 0.080 for observed reflections, residual electron density -0.76 to 0.74 eÅ^{-3}.

2,2'-(2,3-Dibromoanthracene-9,10-diyl)di(benzene-1,3-diol) (**S11**)

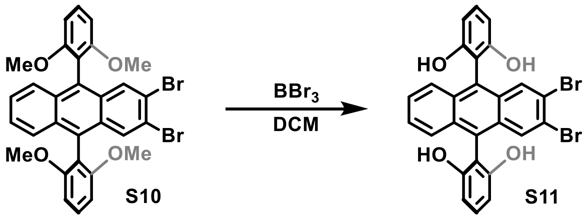

GP3 was applied to **S10** (2.00 g, 3.29 mmol, 1.00 equiv.) and boron tribromide (2.50 mL, 6.59 g, 26.3 mmol, 8.00 equiv.) in 100 mL anhydrous dichloromethane. The reaction mixture was stirred at room temperature for 5 days. Reprecipitation (THF-hexane) yielded **S11** as a pale-yellow solid (978 mg, 1.77 mmol, 54%).

R*f* = 0.29 (SiO_2; petroleum ether/ethyl acetate 2:1, v/v).

Mp: >339 °C (decomposition).

^{1}H NMR ($CDCl_3$, 600 MHz, rt): *δ* = 8.42 (s, 2H), 8.06 - 8.09 (m, 2H), 7.86 (bs, 4H), 7.64 - 7.68 (m, 2H), 7.54 (d, *J* = 8.11 Hz, 2H), 6.87 (d, *J* = 8.11 Hz, 4H) ppm.

^{13}C {^{1}H} NMR ($CDCl_3$, 151 MHz, rt): *δ* = 157.8, 133.2, 132.4, 131.8, 130.7, 130.6, 128.0, 126.6, 121.7, 112.2, 107.9 ppm.

IR (ATR): $\tilde{\nu}$ = 3412, 3158, 2978, 2872, 1614, 1457, 1357, 1250, 999, 946, 883, 762, 724 cm^{-1}.

HRMS (MALDI$^+$) m/z: $[M]^{\cdot+}$: calcd. for $[C_{26}H_{16}O_4{}^{79}Br^{81}Br]^{\cdot+}$: 551.9389; found 551.9396; correct isotope distribution.

Doubly alkylene-bridged 2,3-dibromoanthracene **S12**

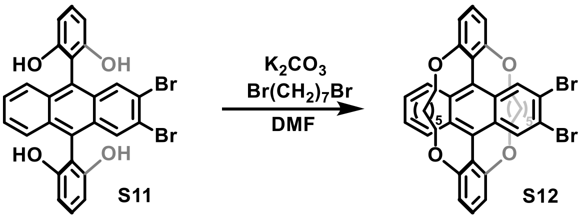

GP4 was applied to **S11** (300 mg, 543 µmol, 1.00 equiv.), potassium carbonate (375 mg, 2.72 mmol, 5.00 equiv.) and 1,7-dibromoheptane (195 µL, 294 mg, 1.14 mmol, 2.10 equiv.) in 120 mL anhydrous dimethylformamide. Flash column chromatography (SiO_2; petroleum ether/dichloromethane 10:1 v/v -> 3:1) yielded **S12** as a pale-yellow solid (233 mg, 313 µmol, 58%).

R*f* = 0.21 (SiO_2; petroleum ether/dichloromethane 2:1, v/v).

Mp: 332 - 335 °C.

^{1}H NMR ($CDCl_3$, 600 MHz, rt): *δ* = 7.94 (s, 2H), 7.57 - 7.60 (m, 2H), 7.46 (t, *J* = 8.33 Hz, 2H), 7.27 - 7.31 (m, 2H), 6.82 (d, *J* = 8.33 Hz, 4H), 3.73 - 3.80 (m, 8H), 1.10 - 1.17 (m, 8H), 0.60 - 0.68 (m, 4H), 0.40 - 0.47 (m, 8H) ppm.

^{13}C {^{1}H} NMR ($CDCl_3$, 151 MHz, rt): *δ* = 158.9, 131.6, 131.2, 130.2, 130.0, 129.9, 127.1, 125.3, 120.5, 117.8, 107.7, 69.6, 29.3, 28.0, 25.2 ppm.

IR (ATR): $\tilde{\nu}$ = 2929, 2857, 1580, 1451, 1250, 1233, 1108, 1089, 758, 724 cm^{-1}.

UV-Vis (*n*-hexane, rt): $\lambda_{max, abs}$ = 405 nm, $\lambda_{max, em}$ = 417 nm, Φ = 0.16.

HRMS (MALDI$^+$) m/z: $[M]^{\cdot+}$: calcd. for $[C_{40}H_{40}O_4{}^{79}Br^{81}Br]^{\cdot+}$: 744.1267; found 744.1269; correct isotope distribution.

6,13-Bis(2,6-dimethoxyphenyl)pentacene (**2**)

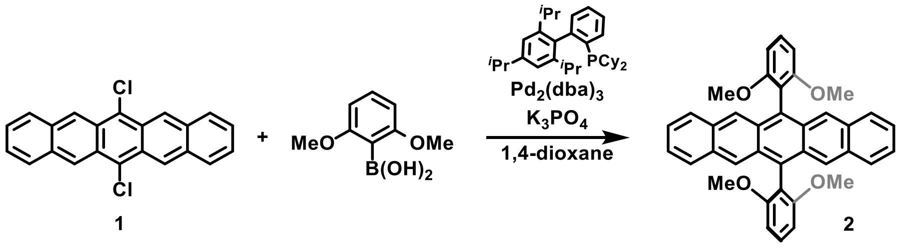

In a heatgun dried Schlenk tube under an atmosphere of argon was added **1** (500 mg, 1.44 mmol, 1.00 equiv.), (2,6-dimethoxyphenyl)boronic acid (1.31 g, 7.20 mmol, 5.00 equiv.), $Pd_2(dba)_3$ (132 mg, 144 µmol, 0.10 equiv) and XPhos (206 mg, 432 µmol, 0.30 equiv). Then, 50 mL freshly degassed 1,4-dioxane and potassium phosphate in distilled water (10 mL, 1.20 $molL^{-1}$) were added, and the reaction mixture stirred at 100 °C for 72 h. The mixture was cooled to room temperature, diluted with water (10 mL) and filtrated over Celite®. The phases were separated, and the aqueous layer was extracted with ethyl acetate (3×10 mL). The combined organic phases were washed with brine (10 mL), dried over magnesium sulfate and filtrated. The solvent was removed under reduced pressure. The crude product **2** was isolated as purple solid (469 mg, 852 µmol, 59%). Due to its low stability, it was used without further purification.

$\mathbf{R}_f$ = 0.21 (SiO_2; petroleum ether/dichloromethane 1:1, v/v).

Mp: >349 °C (decomposition).

^{1}H NMR ($CDCl_3$, 300 MHz, rt): δ = 8.24 (s, 4H), 7.69 - 7.76 (m, 4H), 7.63 (t, J = 8.43 Hz, 2H), 7.13 - 7.19 (m, 4H), 6.93 (d, J = 8.43 Hz, 4H), 3.55 (s, 12H) ppm.

IR (ATR): $\tilde{\nu}$ = 2932, 2826, 1580, 1469, 1428, 1247, 1106, 745, 463 cm^{-1}.

HRMS ($MALDI^+$) m/z: $[M]^{\cdot+}$: calcd. for $[C_{38}H_{30}O_4]^{\cdot+}$: 550.2139; found 550.2141; correct isotope distribution.

2,2'-(Pentacene-6,13-diyl)di(benzene-1,3-diol) (**3**)

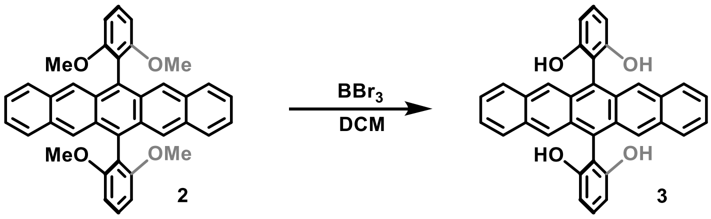

GP3 was applied to crude tetramethyl ether **2** (500 mg, 908 µmol, 1.00 equiv.) and boron tribromide (689 µL, 1.82 g, 7.26 mmol, 8.00 equiv.) in 100 mL freshly degassed, anhydrous dichloromethane. The reaction mixture was stirred at room temperature for 48 h. Reprecipitation (THF-hexane) yielded **3** as a purple solid (422 mg, 853 µmol, 94%). Due to its low stability, crude **3** was used without further purification.

$\mathbf{R}_f$ = 0.25 (SiO_2; petroleum ether/ethyl acetate 2:1, v/v).

^{1}H NMR ($CDCl_3$, 300 MHz, rt): δ = 8.40 (s, 4H), 7.70 - 7.81 (m, 4H), 7.42 (t, J = 8.10 Hz, 2H), 7.21 - 7.32 (m, 4H), 6.78 (d, J = 8.10 Hz, 4H), 5.06 (bs, 4H) ppm.

HRMS (MALDI$^+$) m/z: $[M]^{\cdot+}$: calcd. for $[C_{34}H_{22}O_4]^{\cdot+}$: 494.1539; found 494.1526; correct isotope distribution.

1.2.3 Synthesis of (Aza-)Pentacenes and Tetraazahexacene

Doubly alkylene-bridged pentacene **5**

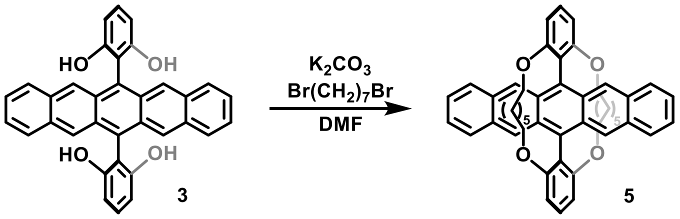

GP4 was applied to **3** (60.0 mg, 121 µmol, 1.00 equiv.), potassium carbonate (83.8 mg, 607 µmol, 5.00 equiv.) and 1,7-dibromoheptane (43.5 µL, 65.7 mg, 255 µmol, 2.10 equiv.) in 25.0 mL anhydrous dimethylformamide. Flash column chromatography (SiO_2; petroleum ether/ethyl acetate 100:1 v/v -> 50:1) yielded **5** as a dark purple solid (25.6 mg, 121 µmol, 31%).

R*f* = 0.55 (SiO_2; petroleum ether/ethyl acetate 4:1, v/v).

Mp: 339 - 342 °C.

^{1}H NMR ($CDCl_3$, 600 MHz, rt): *δ* = 8.28 (s, 4H), 7.73 - 7.78 (m, 4H), 7.58 (t, *J* = 8.44 Hz, 2H), 7.15 - 7.19 (m, 4H), 6.95 (d, *J* = 8.44 Hz, 4H), 3.75 (t, *J* = 5.38 Hz, 8H), 0.92 - 0.98 (m, 8H), 0.32 - 0.39 (m, 4H), 0.12 - 0.18 (m, 8H) ppm.

^{13}C {^{1}H} NMR ($CDCl_3$, 151 MHz, rt): *δ* = 159.5, 131.0, 130.8, 129.7, 129.3, 128.9, 125.4, 124.3, 120.0, 108.3, 70.0, 29.1, 27.7, 24.9 ppm.

IR (ATR): $\tilde{\nu}$ = 2926, 2858, 1575, 1453, 1239, 1088, 871, 724, 464 cm^{-1}.

UV-Vis (*n*-hexane, rt): $\lambda_{max,\ abs}$ = 593 nm, $\lambda_{max,\ em}$ = 599 nm.

HRMS (MALDI$^+$) m/z: $[M]^{\cdot+}$: calcd. for $[C_{48}H_{46}O_4]^{\cdot+}$: 686.3391; found 686.3396; correct isotope distribution.

Crystal data

Single crystalline specimen were obtained by slow diffusion of methanol into a chloroform solution of **5** in a glovebox:

Purple crystal (plate), dimensions 0.140 x 0.105 x 0.025 mm^3, crystal system triclinic, space group $P\bar{1}$, *Z* = 2, *a* = 8.9960(4) Å, *b* = 13.3013(5) Å, *c* = 15.4926(6) Å, *α* = 92.178(3) °, *β* = 91.838(3) °, *γ* = 94.562(3) °, *V* = 1845.47(13) Å^3, *ρ* = 1.236 g/cm^3, *T* = 200(2) K, Θ_{max} = 72.110 °, 19660 reflections measured, 6907 unique (R_{int} = 0.0459), 3971 observed (I > 2σ(I)), *μ* = 0.60 mm^{-1}, T_{min} = 0.54, T_{max} = 1.59, 524 parameters refined, hydrogen atoms were treated using appropriate riding models, goodness of fit 0.98 for observed reflections, final residual values R1(F) = 0.051, wR(F^2) = 0.124 for observed reflections, residual electron density -0.23 to 0.34 eÅ^{-3}.

Doubly alkylene-bridged 5,6,13,14-tetraazapentacene **6**

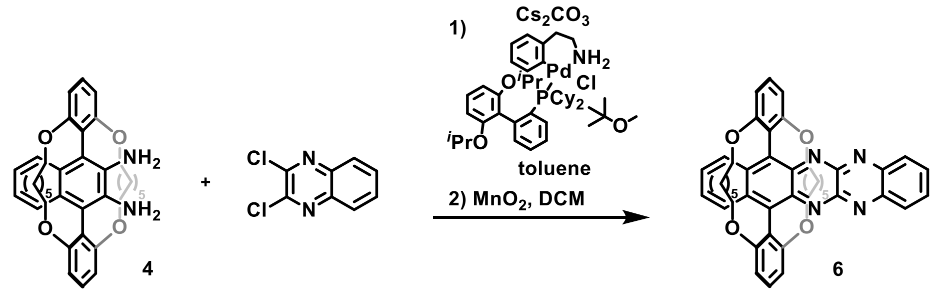

GP5 was applied to **4** (14.0 mg, 24.7 µmol, 1.00 equiv.), 2,3-dichloroquinoxaline (7.37 mg, 37.1 µmol, 1.50 equiv.), caesium carbonate (24.2 mg, 74.1 µmol, 3.00 equiv.) and RuPhos Pd G1 (2.02 mg, 2.47 µmol, 10.0 mol%) in 1.00 mL anhydrous, degassed toluene. Flash column chromatography (SiO_2; petroleum ether/ ethyl acetate 20:1 v/v) yielded **6-H$_2$** as a yellow solid.

R*f* = 0.25 (SiO_2; petroleum ether/ethyl acetate 5:1, v/v).

Crude **6-H$_2$** was dissolved in 10 mL dichloromethane, an excess of manganese dioxide (100 mg) was added and the resulting mixture stirred at room temperature for 15 min. The suspension was filtered through a pad of Celite® and the crude product eluted with dichloromethane. Flash column chromatography (SiO_2; petroleum ether/ethyl acetate 10:1 v/v) and gel permeation chromatography (toluene) yielded **6** as a green solid (15.4 mg, 22.3 µmol, 90% over two steps).

R*f* = 0.04 (SiO_2; petroleum ether/ethyl acetate 5:1, v/v).

Mp: 298°C (decomposition).

^{1}H NMR ($CDCl_3$, 600 MHz, rt): δ = 8.32 - 8.36 (m, 2H), 7.81 - 7.85 (m, 2H), 7.73 - 7.77 (m, 2H), 7.48 (t, J = 8.34 Hz, 2H), 7.31 - 7.34 (m, 2H), 6.86 (d, J = 8.34 Hz, 4H), 3.78 - 3.84 (m, 8H), 1.04 - 1.12 (m, 4H), 0.91 - 0.99 (m, 4H), 0.51 - 0.59 (m, 2H), 0.39 - 0.46 (m, 2H), 0.30 - 0.38 (m, 4H), 0.19 - 0.27 (m, 4H) ppm.

^{13}C {^{1}H} NMR ($CDCl_3$, 151 MHz, rt): δ = 158.9, 148.0, 143.3, 143.1, 135.6, 133.9, 132.5, 130.6, 130.1, 127.9, 127.2, 117.6, 108.1, 69.6, 29.2, 27.9, 25.3 ppm.

IR (ATR): $\tilde{\nu}$ = 2929, 2855, 1582, 1524, 1454, 1374, 1238, 1085, 957, 881, 758, 723, 681, 664, 499 cm^{-1}.

UV-Vis (*n*-hexane, rt): $\lambda_{max,\ abs}$ = 721 nm.

HRMS ($MALDI^+$) m/z: $[M]^{\cdot+}$: calcd. for $[C_{44}H_{44}N_4O_4]^{\cdot+}$: 692.3357; found 692.3365; correct isotope distribution.

Crystal data

Single crystalline specimen were obtained by slow diffusion of methanol into a chloroform solution of **6**:

Green crystal (plank), dimensions 0.147 x 0.035 x 0.015 mm^3, crystal system monoclinic, space group $P2_1/c$, Z = 4, a = 8.6079(4) Å, b = 10.8329(5) Å, c = 42.478(2) Å, α = 90 °, β = 92.120(4) °, γ = 90 °, V = 3958.3(3) Å^3, ρ = 1.360 g/cm^3, T = 200(2) K, Θ_{max} = 67.091 °, 16810 reflections measured, 6374 unique (R_{int} = 0.0351), 3862 observed ($I > 2\sigma(I)$), μ = 2.50 mm^{-1}, T_{min} = 0.72, T_{max} = 1.28, 505 parameters refined, hydrogen atoms were treated using appropriate riding models, goodness of fit 1.02 for observed reflections, final residual values R1(F) = 0.057, wR(F^2) = 0.125 for observed reflections, residual electron density -0.51 to 0.37 eÅ^{-3}.

Doubly alkylene-bridged 6,13-diazapentacene **7**

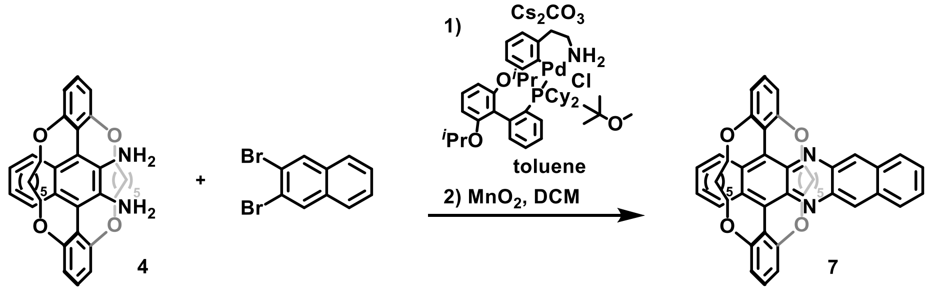

GP5 was applied to **4** (30.0 mg, 52.9 µmol, 1.00 equiv.), 2,3-dibromonaphthalene (22.7 mg, 79.4 µmol, 1.50 equiv.), caesium carbonate (51.7 mg, 159µmol, 3.00 equiv.) and RuPhos Pd G1 (4.32 mg, 5.29 µmol, 10.0 mol%) in 1.00 mL anhydrous, degassed toluene. Intermediately obtained **13-H₂** was dissolved in 10 mL dichloromethane, an excess of manganese dioxide (100 mg) was added and the resulting mixture stirred at room temperature for 15 min. The suspension was filtered through a pad of Celite® and the crude product eluted with dichloromethane. Flash column chromatography (SiO_2; petroleum ether/ethyl acetate 10:1 v/v) and gel permeation chromatography (toluene) yielded **7** as a green solid (24.3 mg, 35.2 µmol, 66% over two steps).

R*f* = 0.32 (SiO_2; petroleum ether/ethyl acetate 5:1, v/v).

Mp: 332 - 337 °C.

^{1}H NMR ($CDCl_3$, 400 MHz, rt): δ = 8.76 (s, 2H), 7.92 - 7.98 (m, 2H), 7.73 - 7.79 (m, 2H), 7.52 (t, *J* = 8.02 Hz, 2H), 7.33 - 7.39 (m, 2H), 7.23 - 7.28 (m, 2H), 6.90 (d, *J* = 8.02 Hz, 4H), 3.75 - 3.88 (m, 8H), 1.01 - 1.13 (m, 4H), 0.87 - 0.99 (m, 4H), 0.47 - 0.60 (m, 2H), 0.16 - 0.46 (m, 10H) ppm.

^{13}C {^{1}H} NMR ($CDCl_3$, 101 MHz, rt): δ = 159.2, 140.7, 140.5, 134.3, 133.8, 132.7, 129.5, 128.8, 128.3, 127.9, 126.1, 125.9, 118.8, 108.0, 69.8, 29.2, 27.9, 25.1 ppm.

IR (ATR): $\tilde{\nu}$ = 2926, 2857, 1586, 1453, 1238, 1106, 1087, 870, 761, 737, 724, 485 cm^{-1}.

UV-Vis (*n*-hexane, rt): $\lambda_{max, abs}$ = 631 nm, $\lambda_{max, em}$ = 652 nm.

HRMS ($MALDI^+$) m/z: $[M+H]^{\cdot+}$: calcd. for $[C_{46}H_{45}N_2O_4]^{\cdot+}$: 689.3374; found 689.3375; correct isotope distribution.

Doubly alkylene-bridged 5,14-diazapentacene **8**

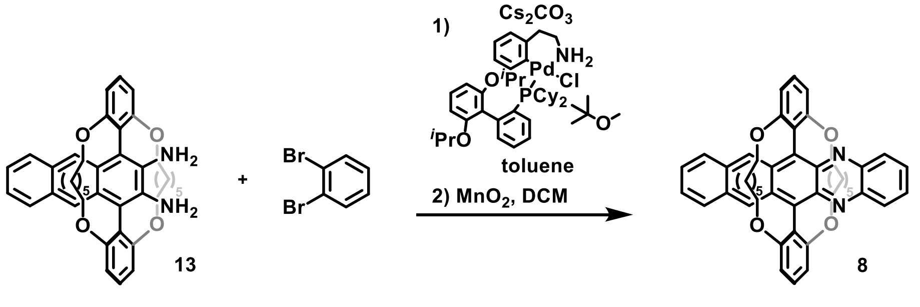

GP5 was applied to **13** (40.0 mg, 64.9 µmol, 1.00 equiv.), 1,2-dibromobenzene (23.0 mg, 97.3 µmol, 1.50 equiv.), caesium carbonate (63.4 mg, 195µmol, 3.00 equiv.) and RuPhos Pd G1 (5.30 mg, 6.49 µmol, 10.0 mol%) in 2.00 mL anhydrous, degassed toluene. Flash column chromatography (SiO_2; petroleum ether/ethyl acetate 20:1 v/v) yielded **8-H₂** as a yellow solid. Crude **8-H₂** was dissolved in 10 mL dichloromethane, an excess of manganese dioxide (100 mg) was added and the resulting mixture stirred at room temperature for 15 min. The suspension was filtered through a pad of Celite® and the crude product was eluted with dichloromethane. Flash column chromatography (SiO_2; petroleum ether/ethyl acetate 10:1 v/v -> 5:1 -> 2:1) and gel permeation chromatography (toluene) yielded **8** as a green solid (35.4 mg, 51.4 µmol, 79% over two steps).

R_f = 0.05 (SiO_2; petroleum ether/ethyl acetate 10:1, v/v).

Mp: 344 - 349 °C.

^{1}H NMR ($CDCl_3$, 400 MHz, rt): δ = 8.47 (s, 2H), 7.94 - 8.02 (m, 2H), 7.75 - 7.82 (m, 2H), 7.55 (t, J = 8.27 Hz, 2H), 7.53 - 7.57 (m, 2H), 7.21 - 7.25 (m, 2H), 6.93 (d, J = 8.27 Hz, 4H), 3.75 - 3.86 (m, 8H), 0.84 - 1.13 (m, 8H), 0.33 - 0.43 (m, 4H), 0.07 - 0.17 (m, 8H) ppm.

^{13}C {^{1}H} NMR ($CDCl_3$, 101 MHz, rt): δ = 159.3, 144.1, 138.0, 133.1, 131.9, 131.7, 130.8, 129.6, 129.5, 128.9, 126.3, 125.3, 119.1, 108.0, 69.7, 29.2, 27.8, 25.0 ppm.

IR (ATR): $\tilde{\nu}$ = 2920, 2854, 1577, 1453, 1235, 1085, 738, 401 cm^{-1}.

UV-Vis (*n*-hexane, rt): $\lambda_{max,\ abs}$ = 663 nm, $\lambda_{max,\ em}$ = 687 nm.

HRMS (MALDI$^+$) m/z: [M]$^{\cdot+}$: calcd. for $[C_{46}H_{44}N_2O_4]^{\cdot+}$: 688.3296; found 688.3302; correct isotope distribution.

Doubly alkylene-bridged 5,14-diazapentacene **9**

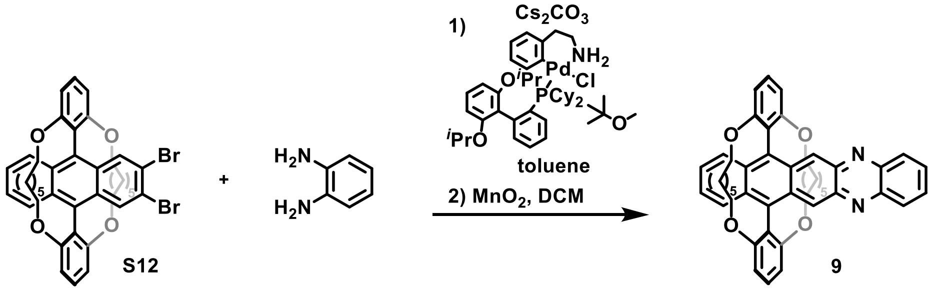

GP5 was applied to **S12** (40.0 mg, 53.7 µmol, 1.00 equiv.), *o*-phenylenediamine (11.6 mg, 107 µmol, 2.00 equiv.), caesium carbonate (52.5 mg, 161 µmol, 3.00 equiv.) and RuPhos Pd G1 (4.39 mg, 5.37 µmol, 10.0 mol%) in 1.50 mL anhydrous, degassed toluene. Intermediately obtained **9-H₂** was dissolved in 10 mL dichloromethane, an excess of manganese dioxide (100 mg) was added and the resulting mixture stirred at room temperature for 15 min. The suspension was filtered through a pad of Celite® and the crude product eluted with dichloromethane. Flash column chromatography (SiO_2; petroleum ether/ethyl acetate 50:1 v/v -> 25:1) and gel permeation chromatography (toluene) yielded a green solid **9** (30.0 mg, 43.6 µmol, 81% over two steps).

R_f = 0.28 (SiO_2; petroleum ether/ethyl acetate 4:1, v/v).

Mp: 298 - 301 °C.

^{1}H NMR ($CDCl_3$, 600 MHz, rt): δ = 8.97 (s, 2H), 8.06 - 8.14 (m, 2H), 7.67 - 7.71 (m, 2H), 7.62 - 7.66 (m, 2H), 7.50 (t, J = 8.35 Hz, 2H), 7.22 - 7.25 (m, 2H), 6.88 (d, J = 8.35 Hz, 4H), 3.73 - 3.81 (m, 8H), 1.04 - 1.12 (m, 4H), 0.94 - 1.02 (m, 4H), 0.47 - 0.56 (m, 2H), 0.38 - 0.46 (m, 2H), 0.22 - 0.37 (m, 8H) ppm.

^{13}C {^{1}H} NMR ($CDCl_3$, 151 MHz, rt): δ = 159.2, 144.6, 138.8, 132.6, 131.7, 131.4, 130.7, 130.1, 129.9, 127.4, 126.9, 125.5, 118.6, 108.2, 69.8, 29.2, 27.9, 25.2 ppm.

IR (ATR): $\tilde{\nu}$ = 2926, 2855, 1579, 1452, 1248, 1087, 1012, 796, 750, 728, 676 cm^{-1}.

UV-Vis (*n*-hexane, rt): $\lambda_{max,\ abs}$ = 668 nm, $\lambda_{max,\ em}$ = 685 nm.

HRMS ($MALDI^+$) m/z: $[M]^{\cdot+}$: calcd. for $[C_{46}H_{44}N_2O_4]^{\cdot+}$: 688.3296; found 688.3308; correct isotope distribution.

Crystal data

Single crystalline specimen were obtained by slow diffusion of methanol into a chloroform solution of **9**:

Green crystal (plate), dimensions 0.200 x 0.050 x 0.015 mm^3, crystal system monoclinic, space group $P2_1/n$, Z = 4, a = 8.3936(8) Å, b = 44.727(3) Å, c = 10.8552(8) Å, α = 90 °, β = 102.143(6) °, γ = 90 °, V = 3984.0(6) $Å^3$, ρ = 1.347 g/cm^3, T = 200(2) K, Θ_{max} = 46.089 °, 11792 reflections measured, 3320 unique (R_{int} = 0.1092), 1571 observed ($I > 2\sigma(I)$), μ = 2.46 mm^{-1}, T_{min} = 0.41, T_{max} = 2.92, 505 parameters refined, hydrogen atoms were treated using appropriate riding models, goodness of fit 0.95 for observed reflections, final residual values R1(F) = 0.091, wR(F^2) = 0.215 for observed reflections, residual electron density -0.33 to 0.63 $eÅ^{-3}$.

Doubly alkylene-bridged 5,6,15,16-tetraazahexacene **13**

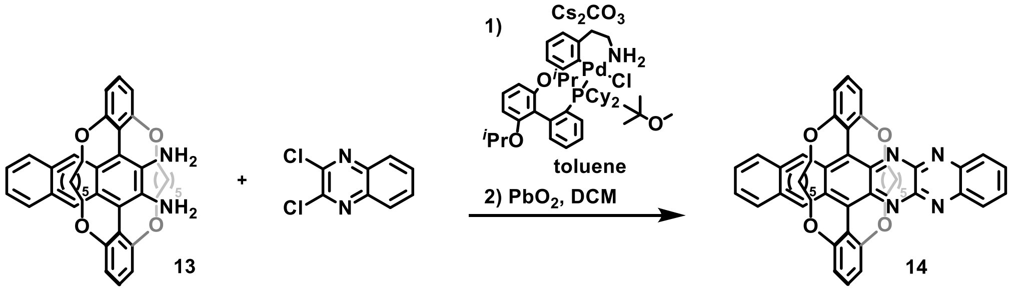

GP5 was applied to **12** (40.0 mg, 64.9 µmol, 1.00 equiv.), 2,3-dichloroquinoxaline (19.4 mg, 97.3 µmol, 1.50 equiv.), caesium carbonate (63.4 mg, 195 µmol, 3.00 equiv.) and RuPhos Pd G1 (5.30 mg, 6.49 µmol, 10.0 mol%) in 1.00 mL anhydrous, degassed toluene. Flash column chromatography (SiO_2; petroleum ether/ ethyl acetate 20:1 v/v -> 10:1) yielded **13-H₂** as a yellow solid.

R_f = 0.54 (SiO_2; petroleum ether/ethyl acetate 2:1, v/v).

Crude **13-H$_2$** was dissolved in 10 mL dichloromethane, an excess of lead dioxide (100 mg) was added and the resulting mixture stirred at 0 °C for 15 min. The suspension was filtered through a pad of Celite® and the crude product eluted with dichloromethane. Flash column chromatography (SiO_2; petroleum ether/ethyl acetate 10:1 v/v -> 5:1 -> 2:1) yielded a brown solid **13** (25.4 mg, 34.3 µmol, 53% over two steps).

R_f = 0.20 (SiO_2; petroleum ether/ethyl acetate 2:1, v/v).

Mp: ≥350 °C.

^{1}H NMR (CD_2Cl_2, 300 MHz, rt): *δ* = 8.45 (s, 2H), 8.18 - 8.26 (m, 2H), 7.80 - 7.87 (m, 2H), 7.73 - 7.80 (m, 2H), 7.63 (t, *J* = 8.31 Hz, 2H), 7.23 - 7.31 (m, 2H), 6.98 (d, *J* = 8.31 Hz, 4H), 3.79 - 3.85 (t, *J* = 5.36 Hz, 8H), 0.86 - 1.02 (m, 8H), 0.30 - 0.44 (m, 4H), 0.06 - 0.19 (m, 8H) ppm.

^{13}C {^{1}H} NMR (CD_2Cl_2, 151 MHz, rt): *δ* = 159.4, 149.0, 143.7, 141.6, 134.7, 133.5, 133.3, 133.2, 130.9, 130.5, 129.1, 126.8, 126.7, 118.3, 108.0, 69.9, 29.4, 28.0, 25.5 ppm.

IR (ATR): $\tilde{\nu}$ = 2931, 2863, 1591, 1456, 1378, 1241, 1091, 728 cm^{-1}.

UV-Vis (*n*-hexane, rt): $\lambda_{max,\ abs}$ = 948 nm.

HRMS (MALDI$^+$) m/z: $[M+2H]^{\cdot+}$: calcd. for $[C_{48}H_{46}N_4O_4]^{\cdot+}$: 742.3514; found 742.3514; correct isotope distribution.

Crystal data

Single crystalline specimen were obtained by slow diffusion of methanol into a chloroform solution of **13**:

Green/brown crystal (plate), dimensions 0.200 x 0.062 x 0.033 mm^3, crystal system monoclinic, space group $P2_1/n$, *Z* = 4, *a* = 11.0098(7) Å, *b* = 21.2505(11) Å, *c* = 19.8994(14) Å, *α* = 90 °, *β* = 98.296(6) °, *γ* = 90 °, *V* = 4607.0(5) $Å^3$, *ρ* = 1.240 g/cm^3, *T* = 200(2) K, Θ_{max} = 45.541 °, 16226 reflections measured, 3808 unique (R_{int} = 0.0854), 2017 observed (I > 2σ(I)), *μ* = 2.18 mm^{-1}, T_{min} = 0.46, T_{max} = 1.84, 541 parameters refined, hydrogen atoms were treated using appropriate riding models, goodness of fit 0.99 for observed reflections, final residual values R1(F) = 0.093, wR(F^2) = 0.245 for observed reflections, residual electron density -0.23 to 0.43 e$Å^{-3}$.

1.2.4 Degradation Experiments

Irradiation of doubly alkylene-bridged pentacene **5** to yield decomposition product **10**

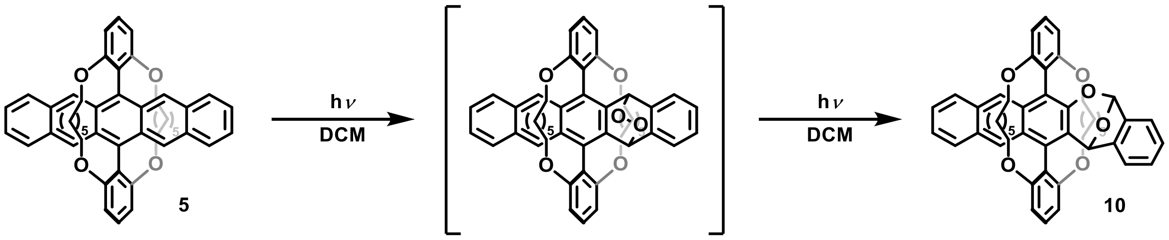

5 (10.0 mg, 14.6 µmol, 1.00 eq.) was dissolved in 5.00 mL dichloromethane in a quartz flask. The reaction mixture was stirred at room temperature for 5 h under irradiation with a handheld UV lamp (λ_1 = 365 nm and λ_2 = 254 nm) whilst air was bubbled through the solution. The solvent was removed under reduced pressure to yield crude **10** as well as several other unidentified products.

HRMS (MALDI$^+$) m/z: $[M+H]^{\cdot+}$: calcd. for $[C_{48}H_{46}O_6]^{\cdot+}$: 718.3289; found 718.3296; correct isotope distribution.

Crystal data

Single crystalline specimen were obtained by slow diffusion of methanol into a dichloromethane solution of crude **10**:

Violet crystal (brick), dimensions 0.145 x 0.105 x 0.075 mm^3, crystal system orthorhombic, space group Pbca, Z = 8, a = 13.1467(7) Å, b = 15.9964(7) Å, c = 36.0630(13) Å, α = 90 °, β = 90 °, γ = 90 °, V = 7584.0(6) $Å^3$, ρ = 1.259 g/cm^3, T = 200 (2) K, Θ_{max} = 52.629 °, 20516 reflections measured, 4335 unique (R_{int} = 0.0418), 2684 observed (I > 2σ(I)), μ = 0.65 mm^{-1}, T_{min} = 0.53, T_{max} = 1.40, 487 parameters refined, hydrogen atoms were treated using appropriate riding models, goodness of fit 1.05 for observed reflections, final residual values R1(F) = 0.102, wR(F^2) = 0.295 for observed reflections, residual electron density -0.33 to 0.61 e$Å^{-3}$.

Irradiation of doubly alkylene-bridged pentacene **7** to yield decomposition product **11**

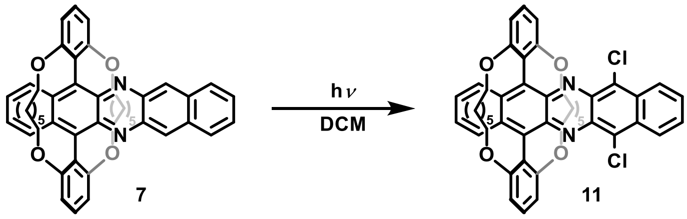

7 (10.0 mg, 14.5 µmol, 1.00 eq.) was dissolved in 5.00 mL dichloromethane in a quartz flask. The reaction mixture was stirred at room temperature for 48 h under irradiation with a handheld UV lamp (λ_1 = 365 nm and λ_2 = 254 nm) whilst air was bubbled through the solution. The solvent was removed under reduced pressure to yield crude **11** among other unidentified decomposition products.

MS ($MALDI^+$) m/z: $[M+2H]^{\cdot+}$: found. for $[C_{46}H_{44}Cl_2N_2O_4]^{\cdot+}$: 758.421.

Crystal data

Single crystalline specimen were obtained by slow diffusion of methanol into a dichloromethane solution of crude **11**:

Brown crystal (plate), dimensions 0.110 x 0.080 x 0.018 mm^3, crystal system triclinic, space group P$\bar{1}$, Z = 2, a = 10.5083(5) Å, b = 11.8968(6) Å, c = 18.9390(10) Å, α = 78.795(4) °, β = 76.419(4) °, γ = 64.321(4) °, V = 2062.5(2) $Å^3$, ρ = 1.412 g/cm^3, T = 200 (2) K, Θ_{max} = 52.626 °, 14869 reflections measured, 4702 unique (R_{int} = 0.0466), 2523 observed (I > 2σ(I)), μ = 3.59 mm^{-1}, T_{min} = 0.70, T_{max} = 1.48, 523 parameters refined, hydrogen atoms were treated using appropriate riding models, goodness of fit 1.14 for observed reflections, final residual values R1(F) = 0.102, wR(F^2) = 0.288 for observed reflections, residual electron density -0.68 to 0.47 e$Å^{-3}$.

2 Results and Discussion

2.1 Calculations

All calculations were performed using Gaussian16. TMS groups were used instead of TIPS groups to simplify calculations. First, the gas-phase ground-state equilibrium geometry of the molecules was optimized at the B3LYP/def2-SVP level of theory. Afterwards, the recieved geometries were refined using the B3LYP/def2-TZVP level of theory. FMO calculations were performed starting from the optimized geometries on the B3LYP/def2-TZVP level of theory.

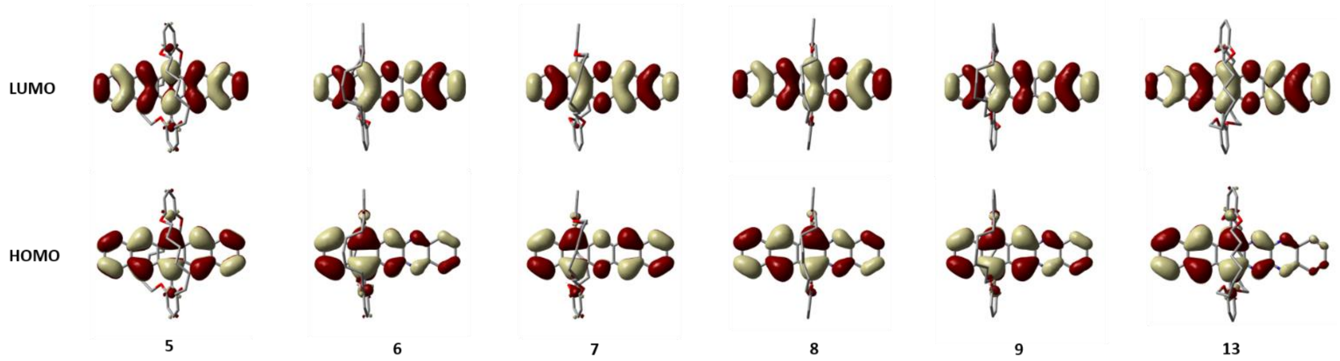

Figure S2. FMO distribution of **5**-**9** and **13**.

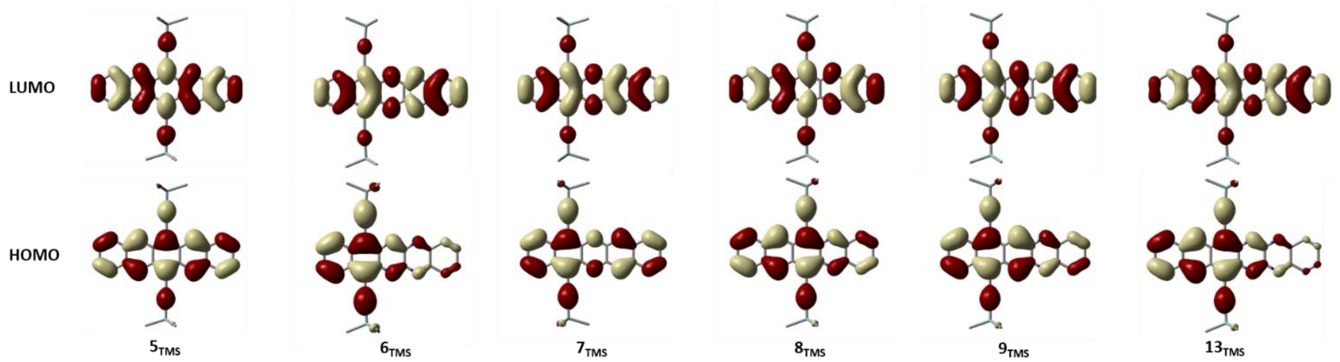

Figure S3. FMO distribution of **5_{TMS}**-**9_{TMS}** and **13_{TMS}**.

2.2 NMR Spectroscopy

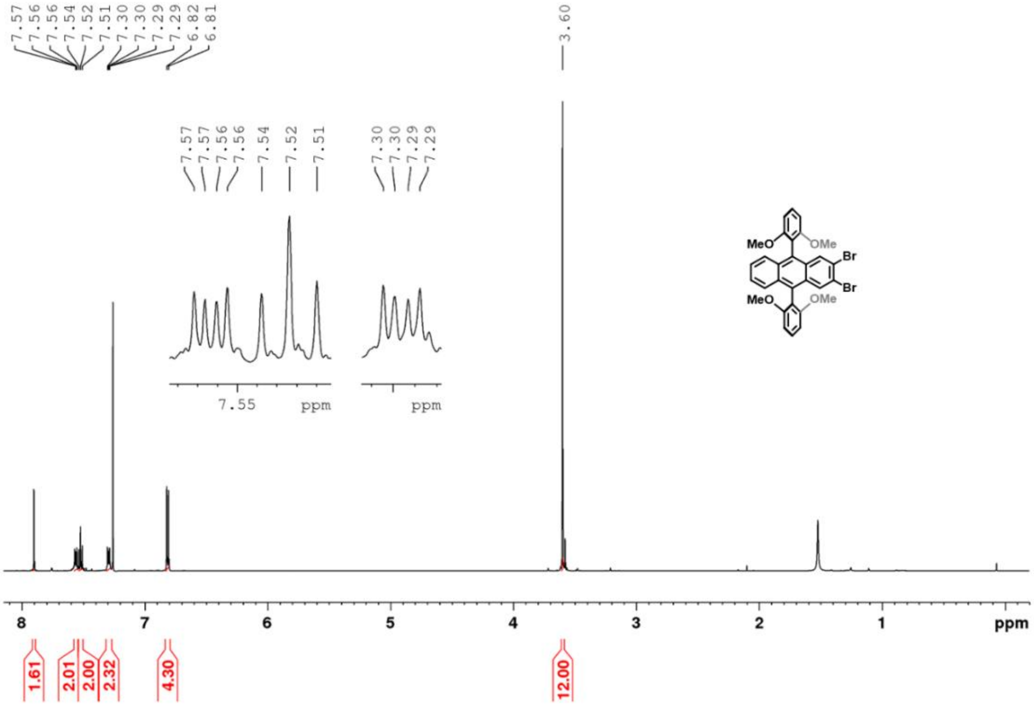

Figure S4: ^{1}H NMR spectrum (600 MHz) of **S10** in $CDCl_3$.

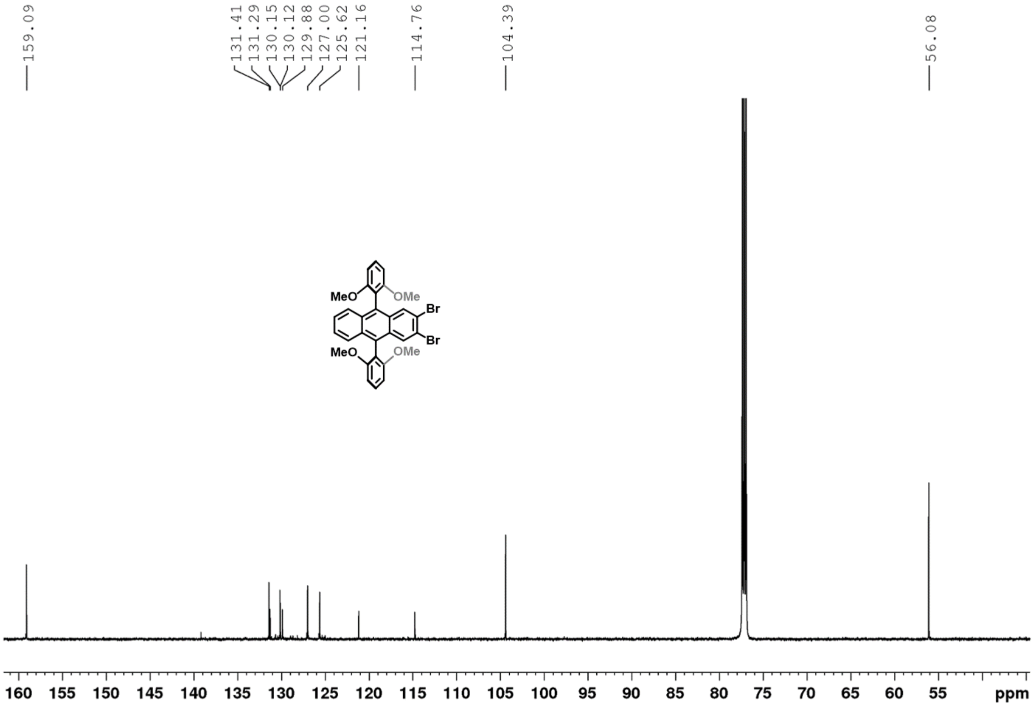

Figure S5: $^{13}C\{^{1}H\}$ NMR spectrum (151 MHz) of **S10** in $CDCl_3$.

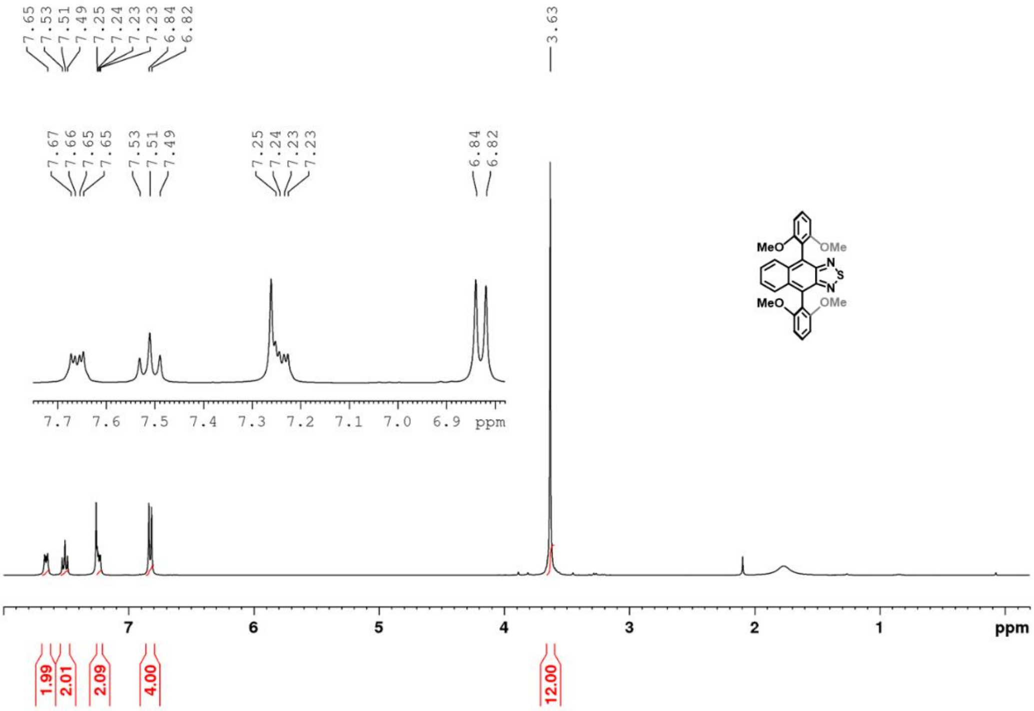

Figure S6: ^{1}H NMR spectrum (400 MHz) of **S3** in $CDCl_3$.

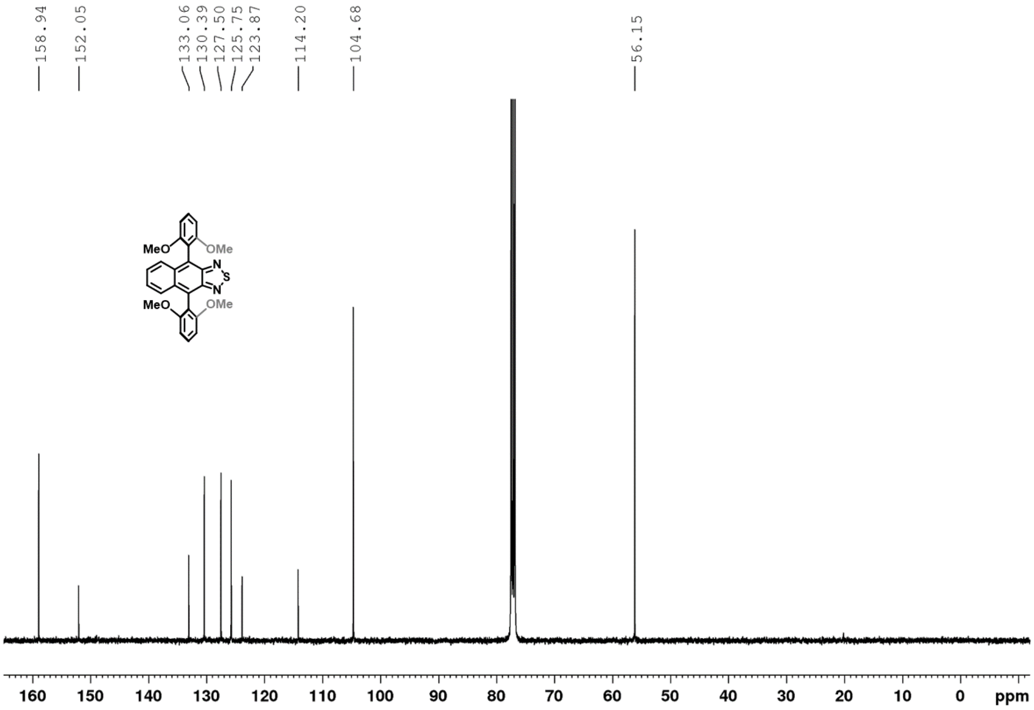

Figure S7: ^{13}C{^{1}H} NMR spectrum (101 MHz) of **S3** in $CDCl_3$.

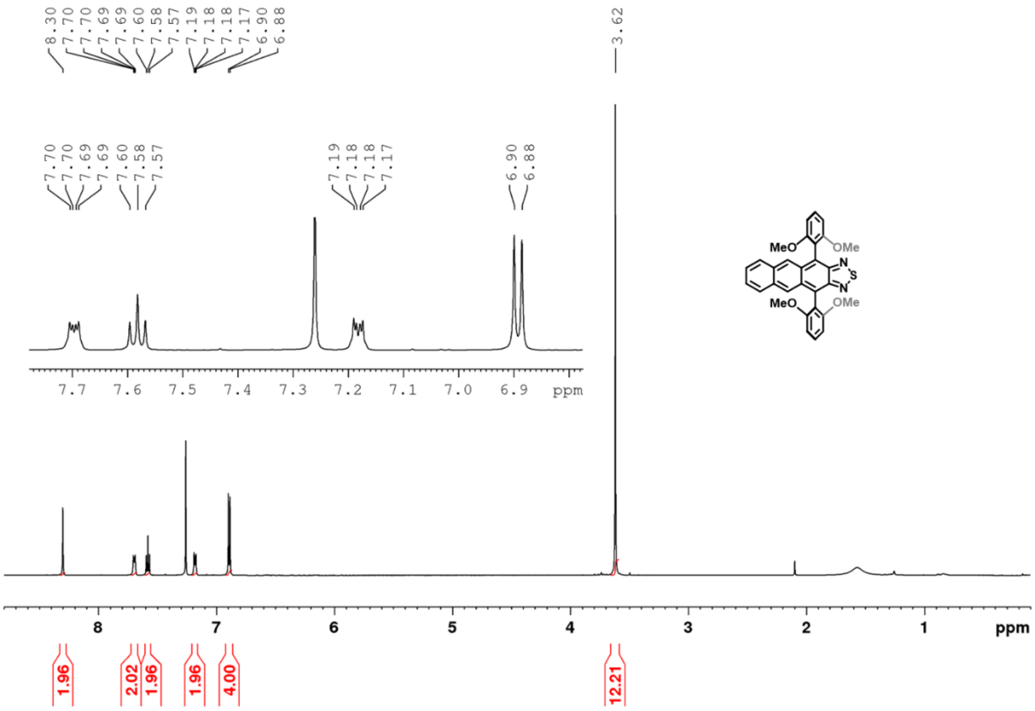

Figure S8: ^{1}H NMR spectrum (600 MHz) of **S4** in $CDCl_3$.

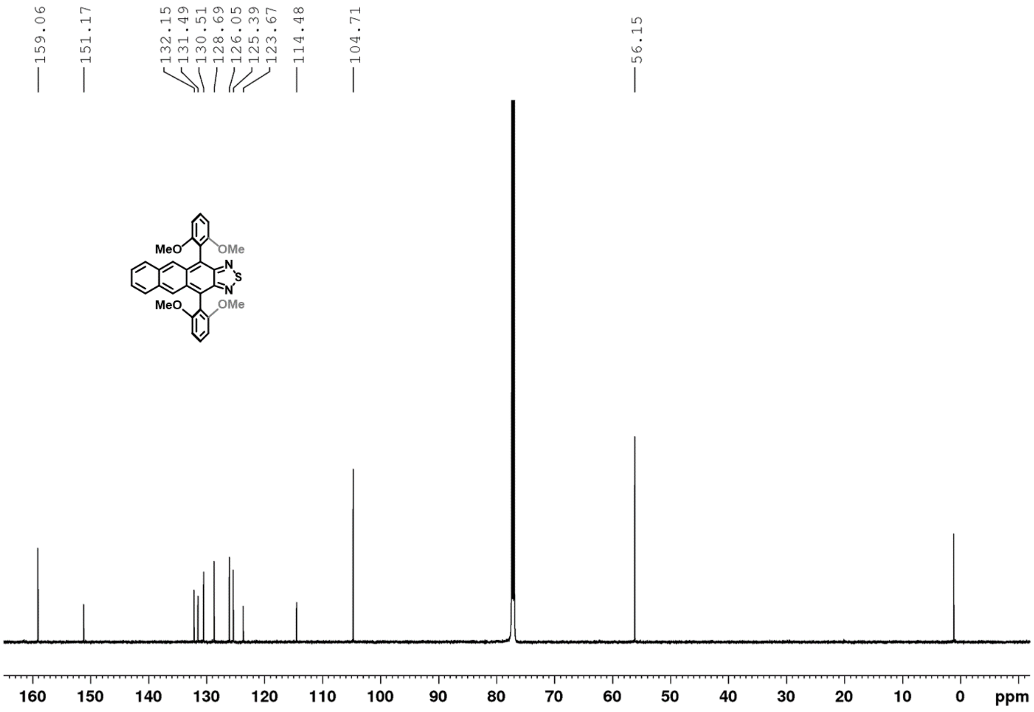

Figure S9: ^{13}C{^{1}H} NMR spectrum (151 MHz) of **S4** in $CDCl_3$.

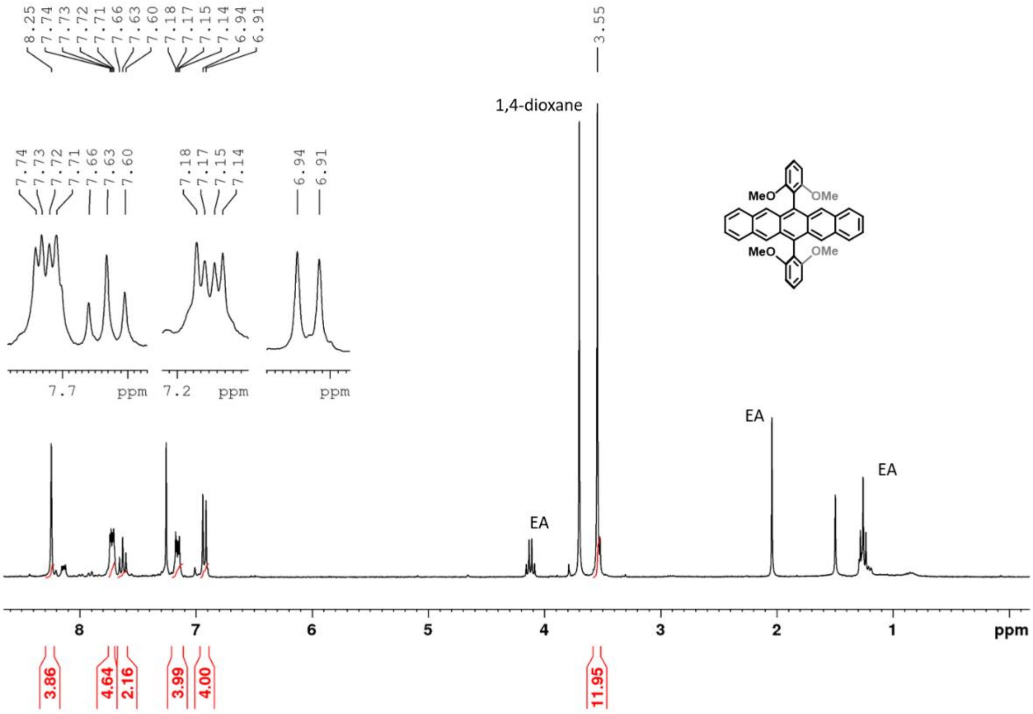

Figure S10: ^{1}H NMR spectrum (300 MHz) of crude **2** in $CDCl_3$. **2** was used in the proceeding synthetic step without further purification.

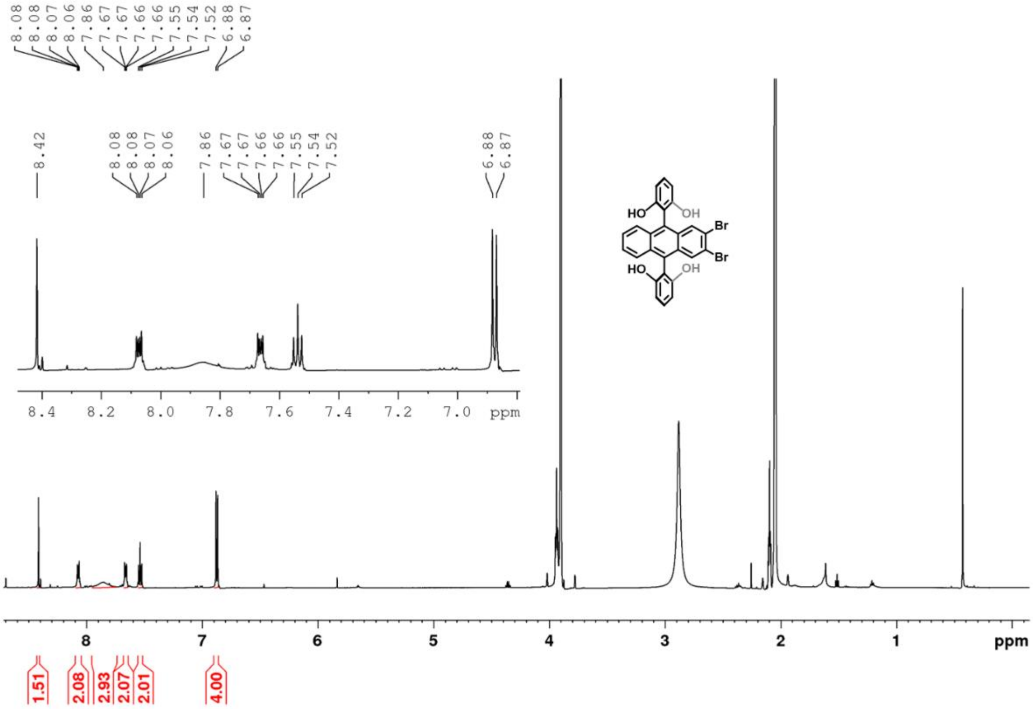

Figure S11: ^{1}H NMR spectrum (600 MHz) of **S11** in THF-d_8.

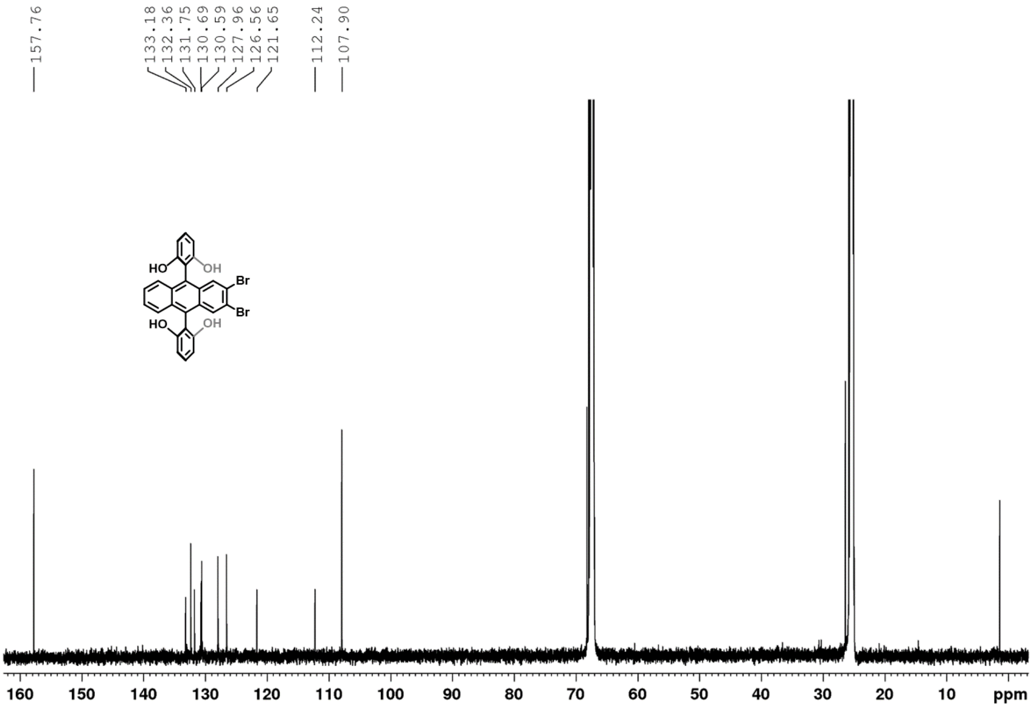

Figure S12: $^{13}C\{^{1}H\}$ NMR spectrum (151 MHz) of **S11** in THF-d_8.

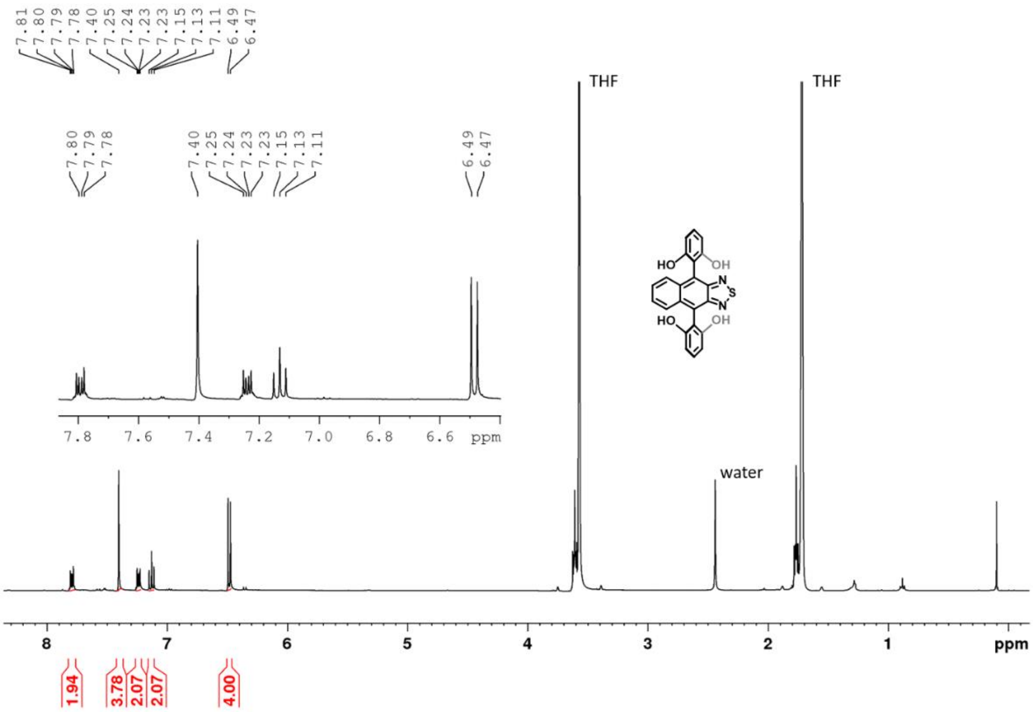

Figure S13: ^{1}H NMR spectrum (400 MHz) of **S5** in THF-d_8.

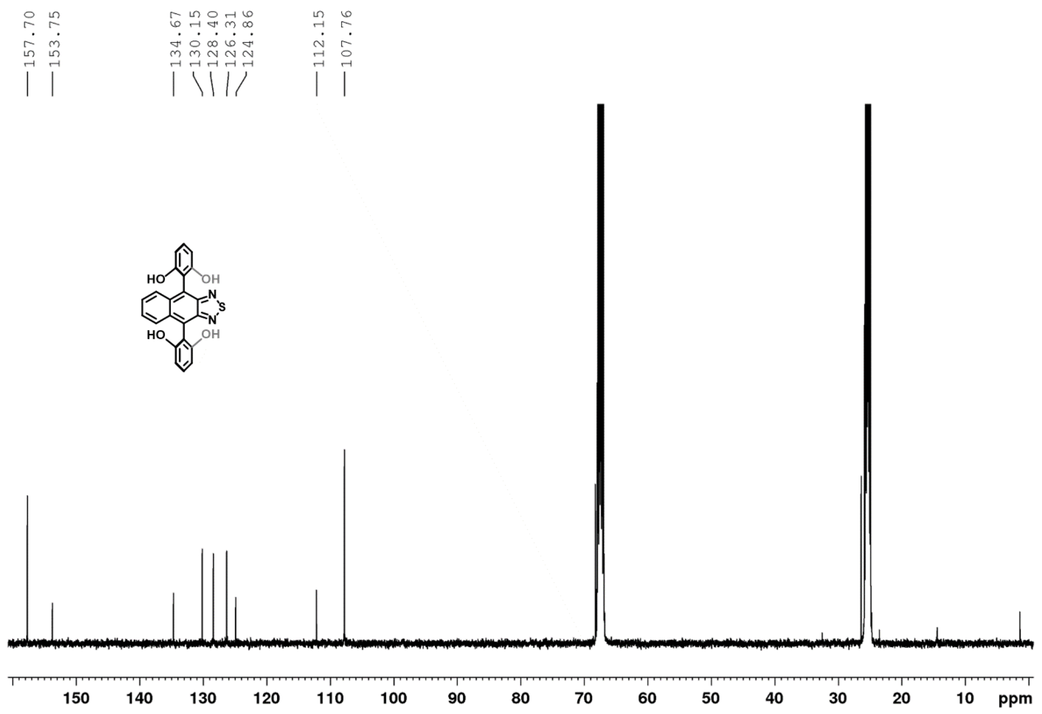

Figure S14: $^{13}C\{^{1}H\}$ NMR spectrum (101 MHz) of **S5** in THF-d_8.

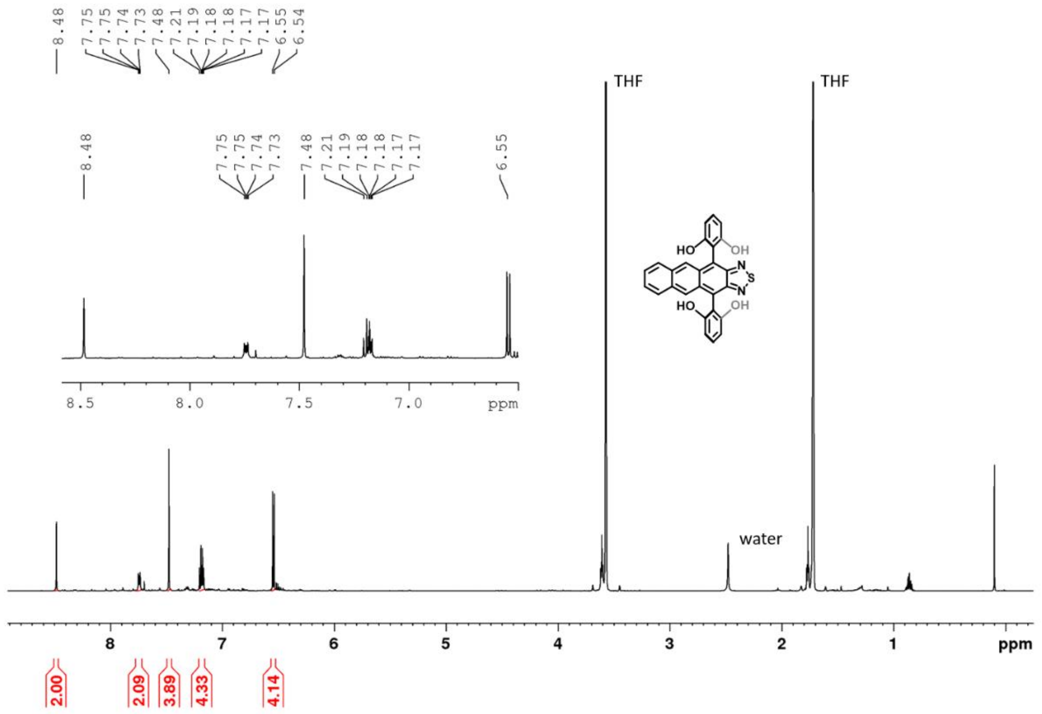

Figure S15: ^{1}H NMR spectrum (400 MHz) of **S6** in THF-d_8.

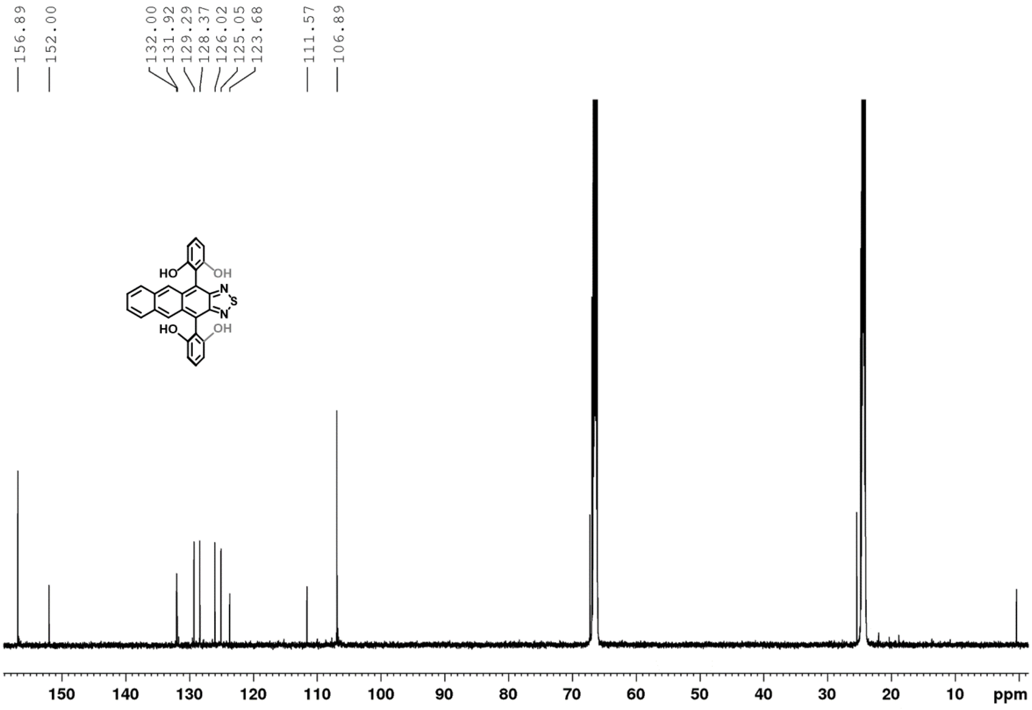

Figure S16: $^{13}C\{^{1}H\}$ NMR spectrum (151 MHz) of **S6** in THF-d_8.

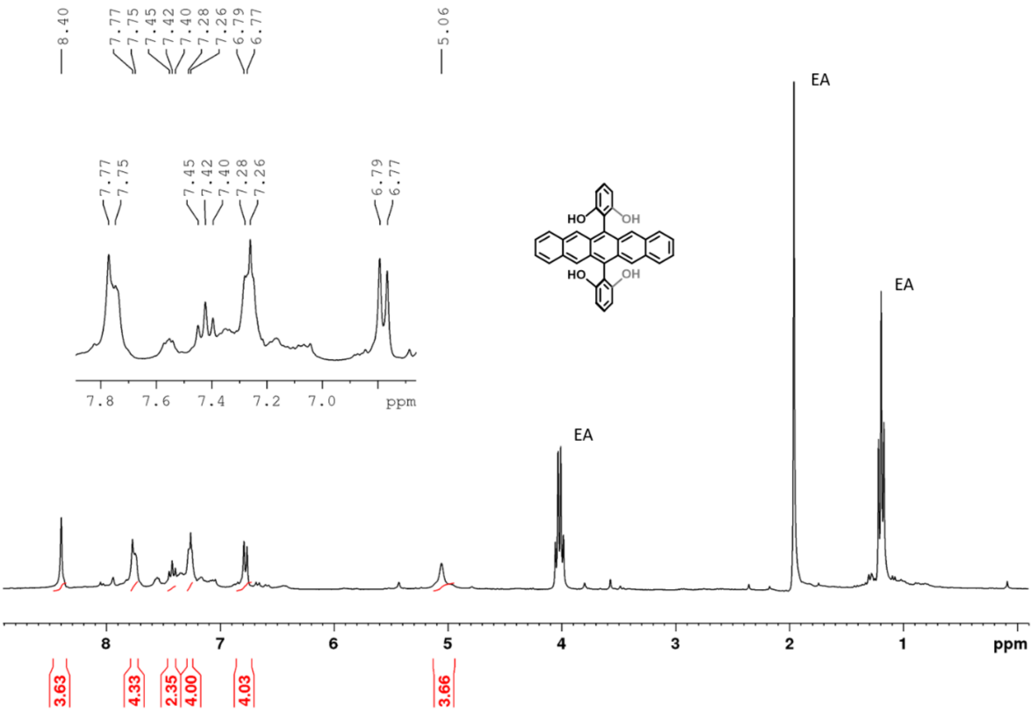

Figure S17: ^{1}H NMR spectrum (300 MHz) of crude **3** in $CDCl_3$. **3** was used in the proceeding synthetic step without further purification.

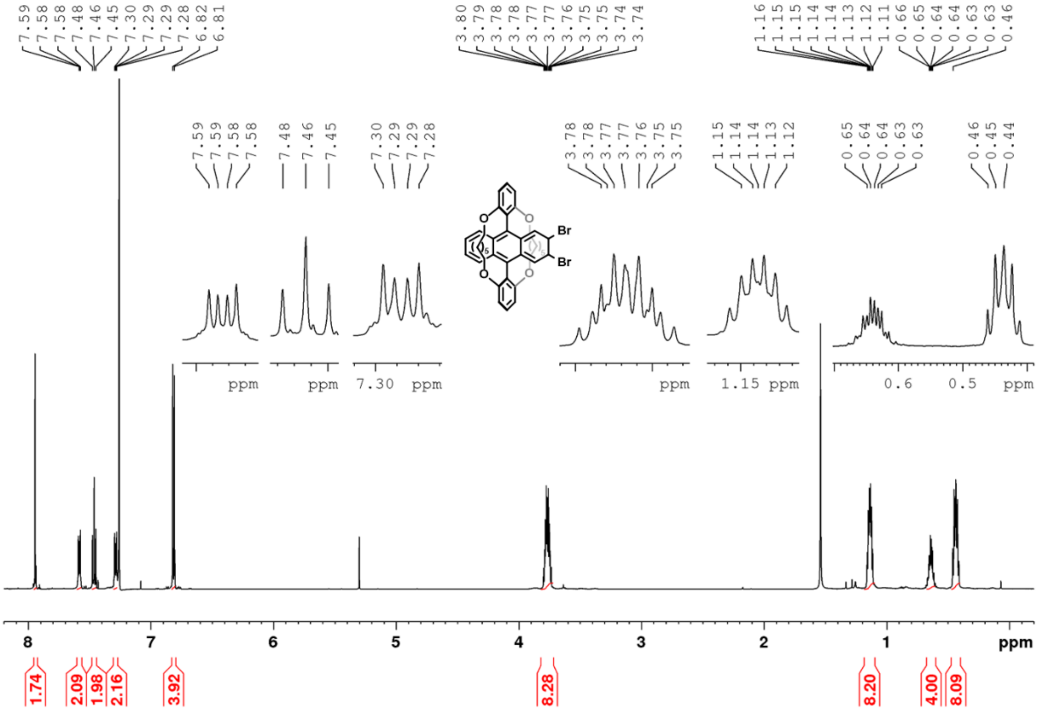

Figure S18: ^{1}H NMR spectrum (600 MHz) of **S12** in $CDCl_3$.

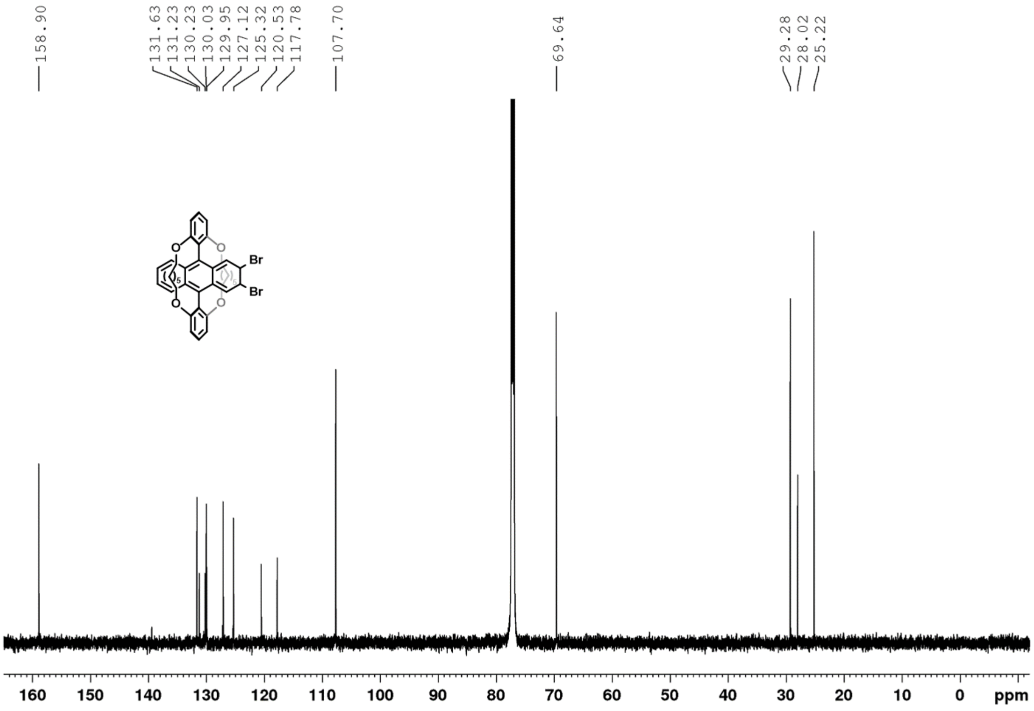

Figure S19: $^{13}C\{^{1}H\}$ NMR spectrum (151 MHz) of **S12** in $CDCl_3$.

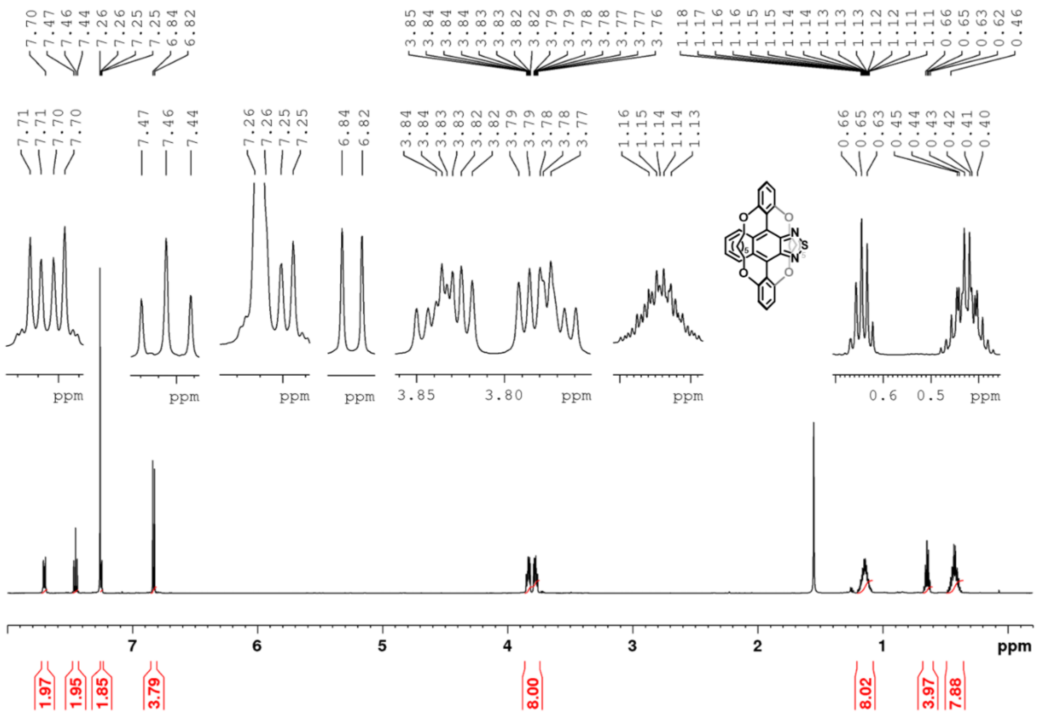

Figure S20: ^{1}H NMR spectrum (600 MHz) of **S7** in $CDCl_3$.

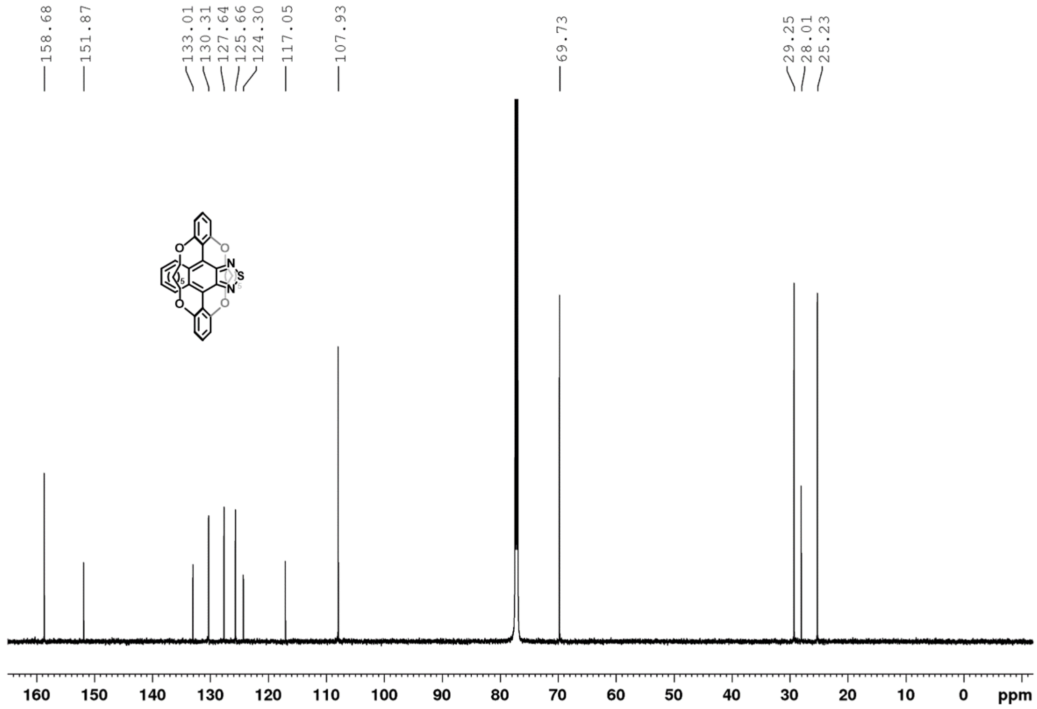

Figure S21: $^{13}C\{^{1}H\}$ NMR spectrum (151 MHz) of **S7** in $CDCl_3$.

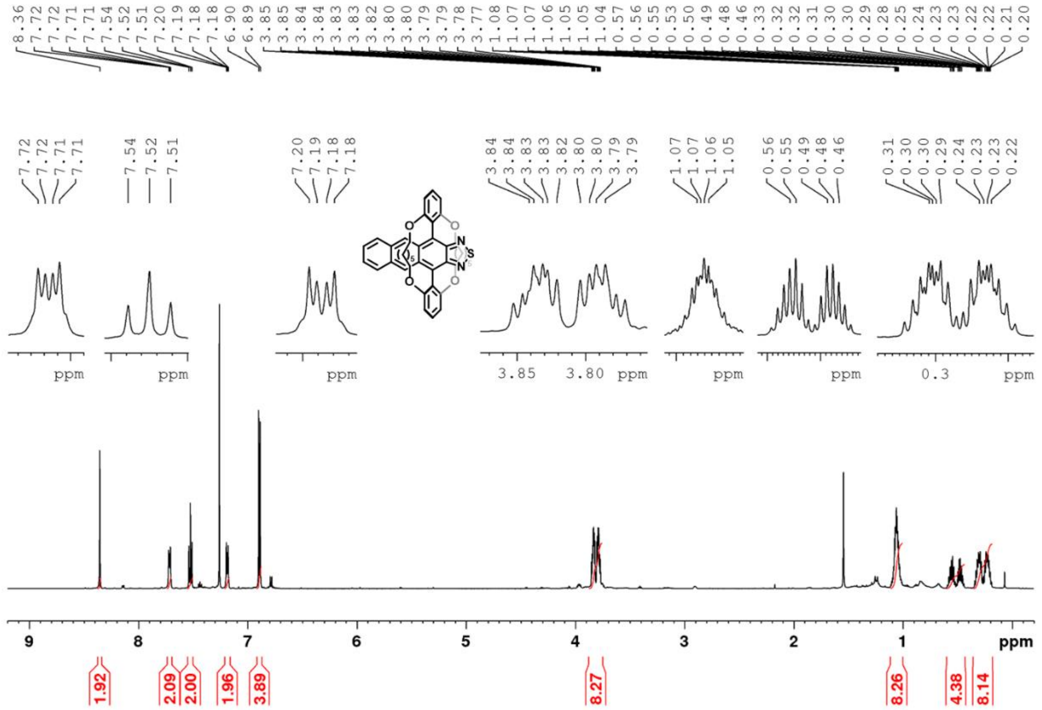

Figure S22: ^{1}H NMR spectrum (600 MHz) of **S8** in $CDCl_3$.

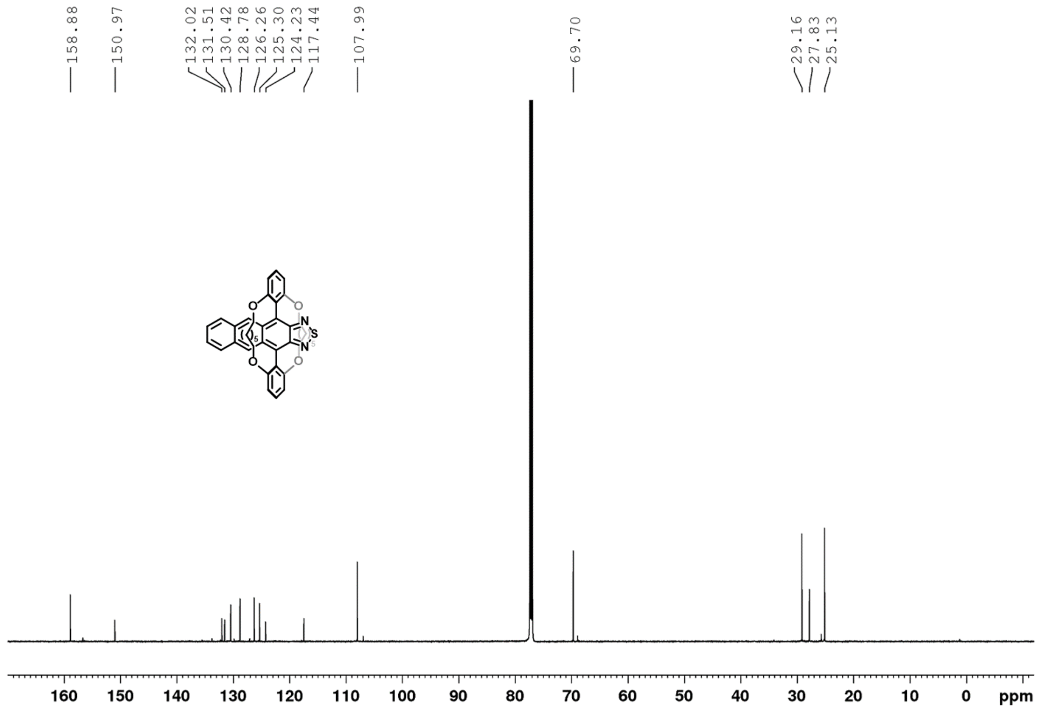

Figure S23: $^{13}C\{^{1}H\}$ NMR spectrum (151 MHz) of **S8** in $CDCl_3$.

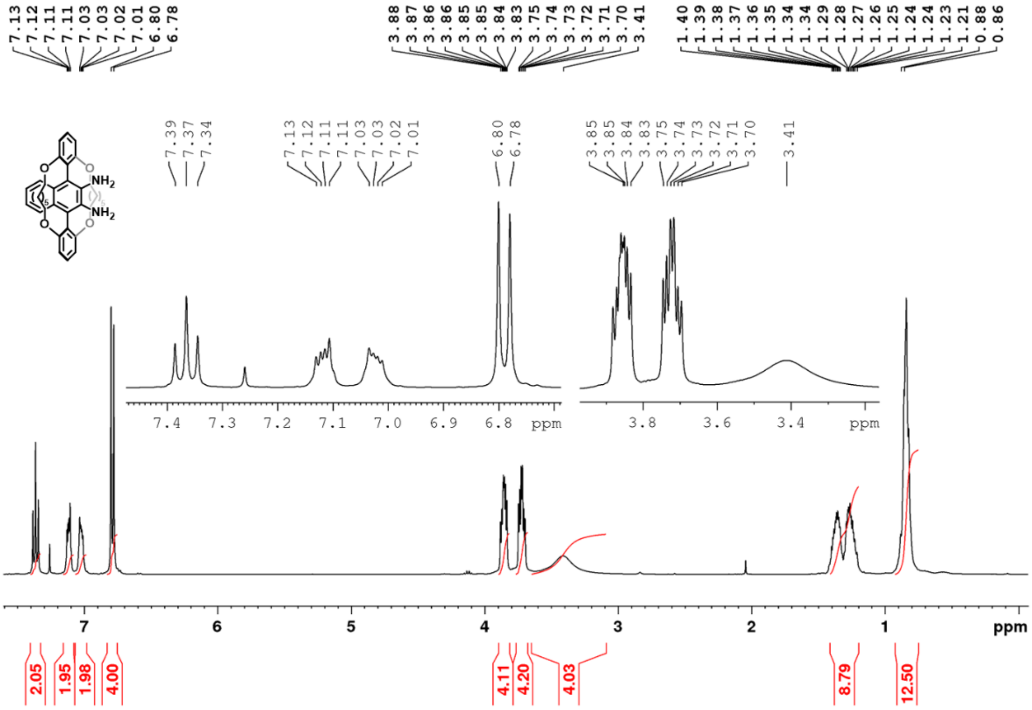

Figure S24: ^{1}H NMR spectrum (400 MHz) of **4** in $CDCl_3$.

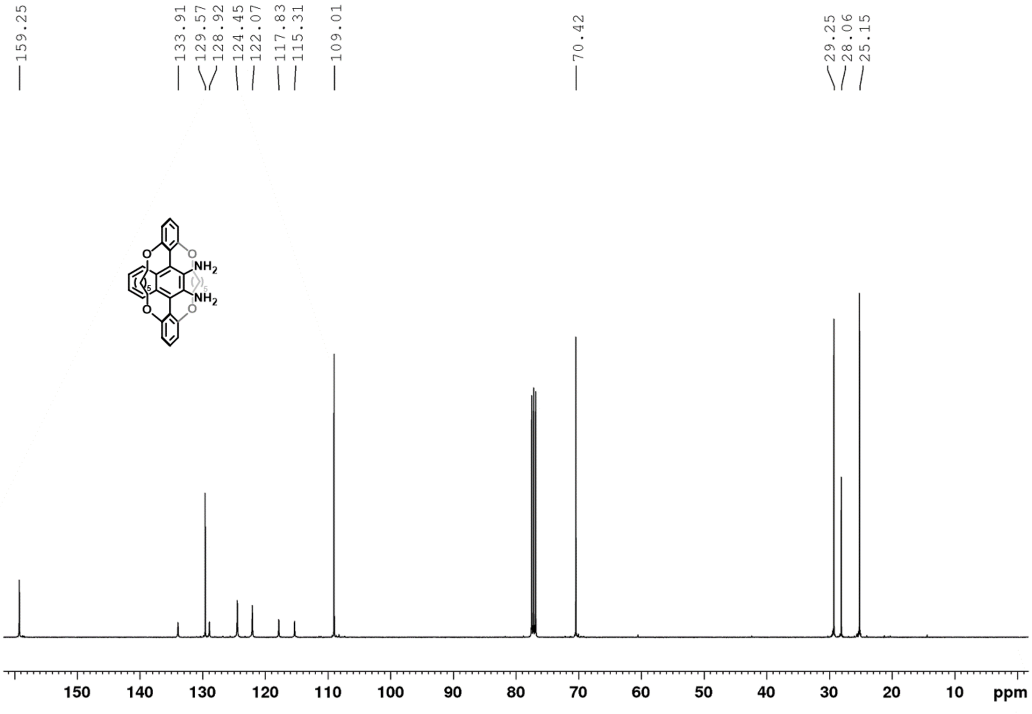

Figure S25: $^{13}C\{^{1}H\}$ NMR spectrum (101 MHz) of **4** in $CDCl_3$.

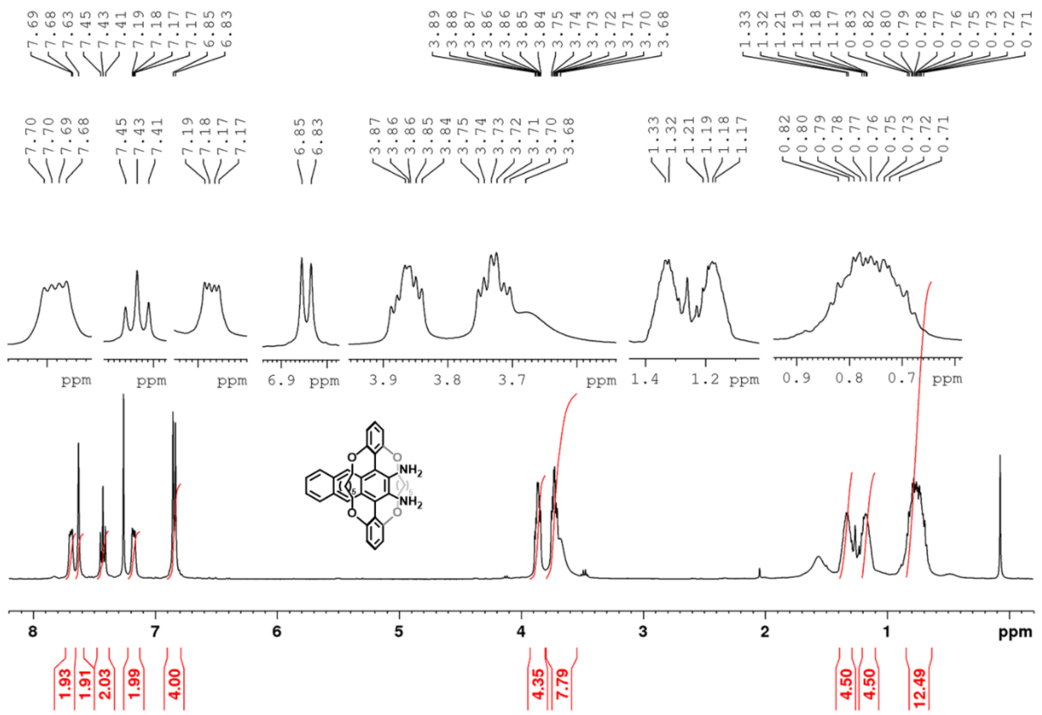

Figure S26: ^{1}H NMR spectrum (400 MHz) of **12** in $CDCl_3$.

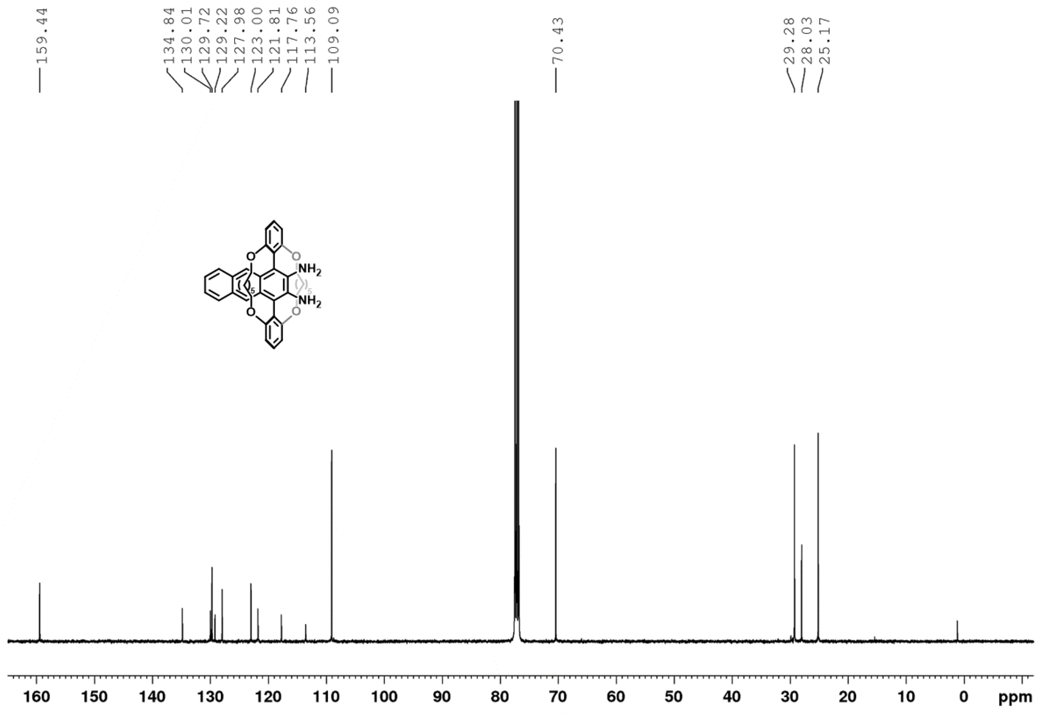

Figure S27: $^{13}C\{^{1}H\}$ NMR spectrum (101 MHz) of **12** in $CDCl_3$.

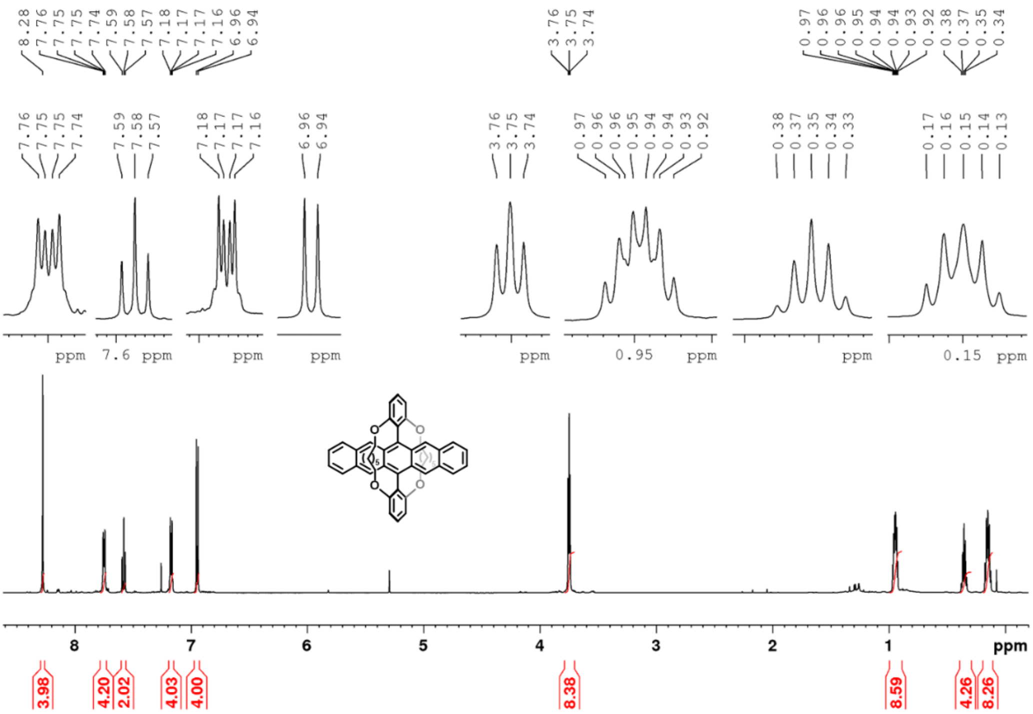

Figure S28: ^{1}H NMR spectrum (400 MHz) of **5** in $CDCl_3$.

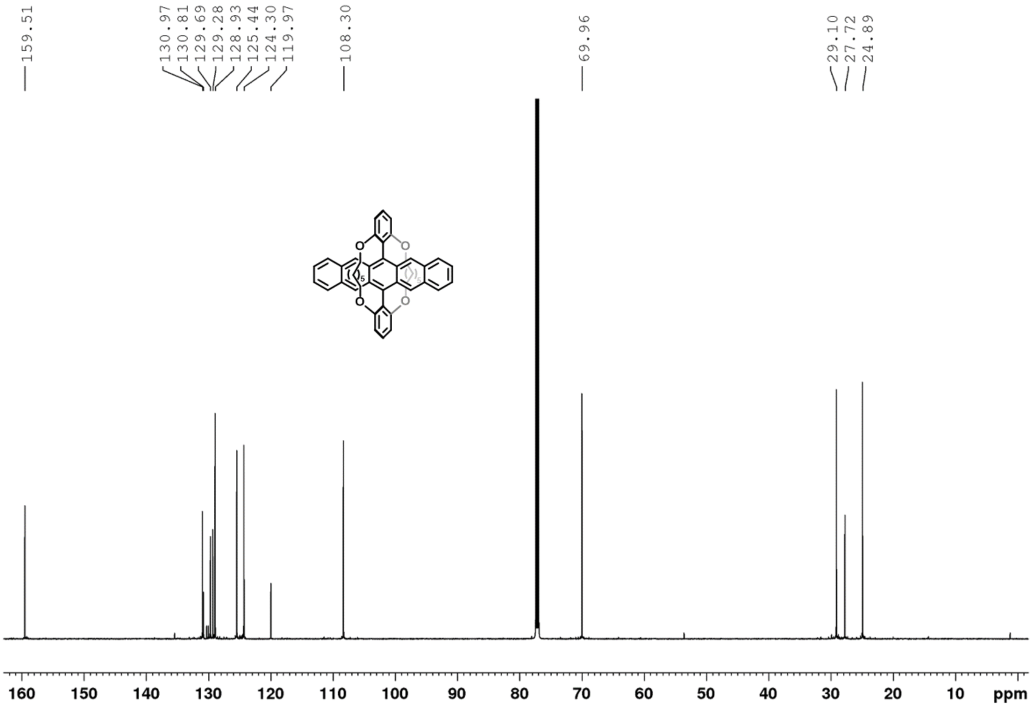

Figure S29: $^{13}C\{^{1}H\}$ NMR spectrum (101 MHz) of **5** in $CDCl_3$.

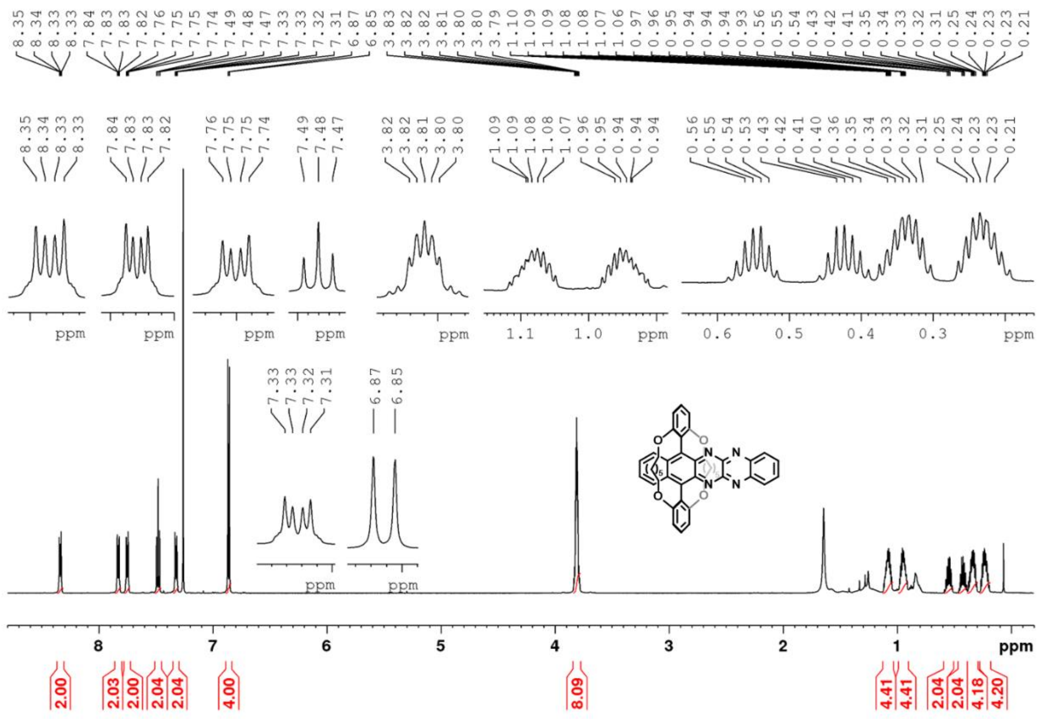

Figure S30: ^{1}H NMR spectrum (600 MHz) of **6** in $CDCl_3$.

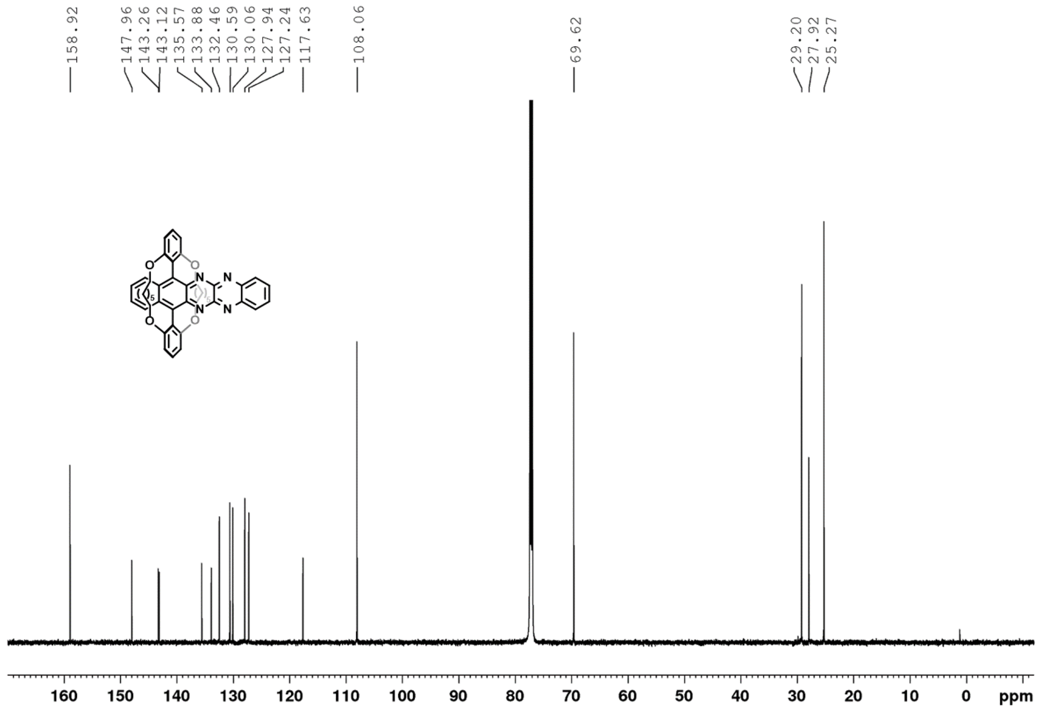

Figure S31: $^{13}C\{^{1}H\}$ NMR spectrum (151 MHz) of **6** in $CDCl_3$.

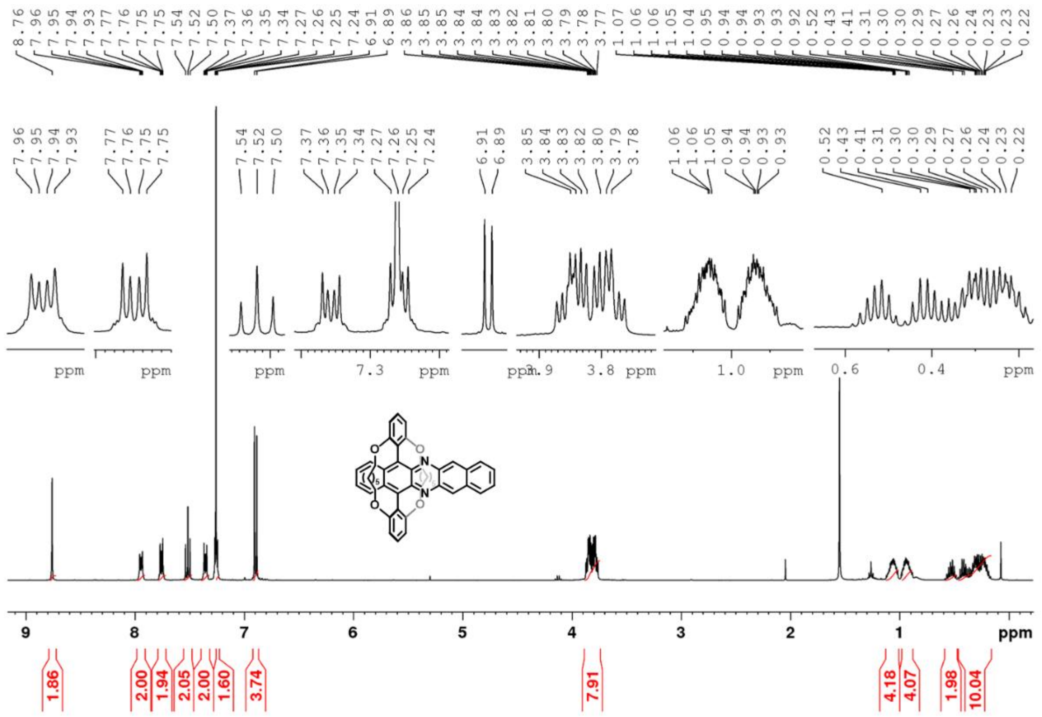

Figure S32: ^{1}H NMR spectrum (400 MHz) of **7** in $CDCl_3$.

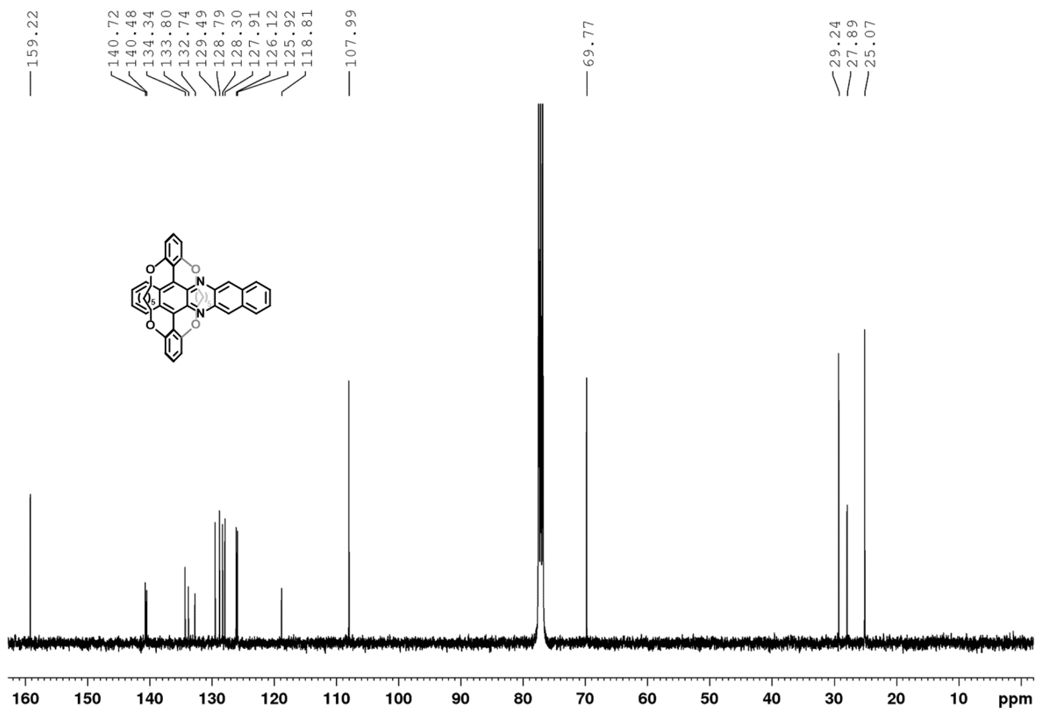

Figure S33: $^{13}C\{^{1}H\}$ NMR spectrum (101 MHz) of **7** in $CDCl_3$.

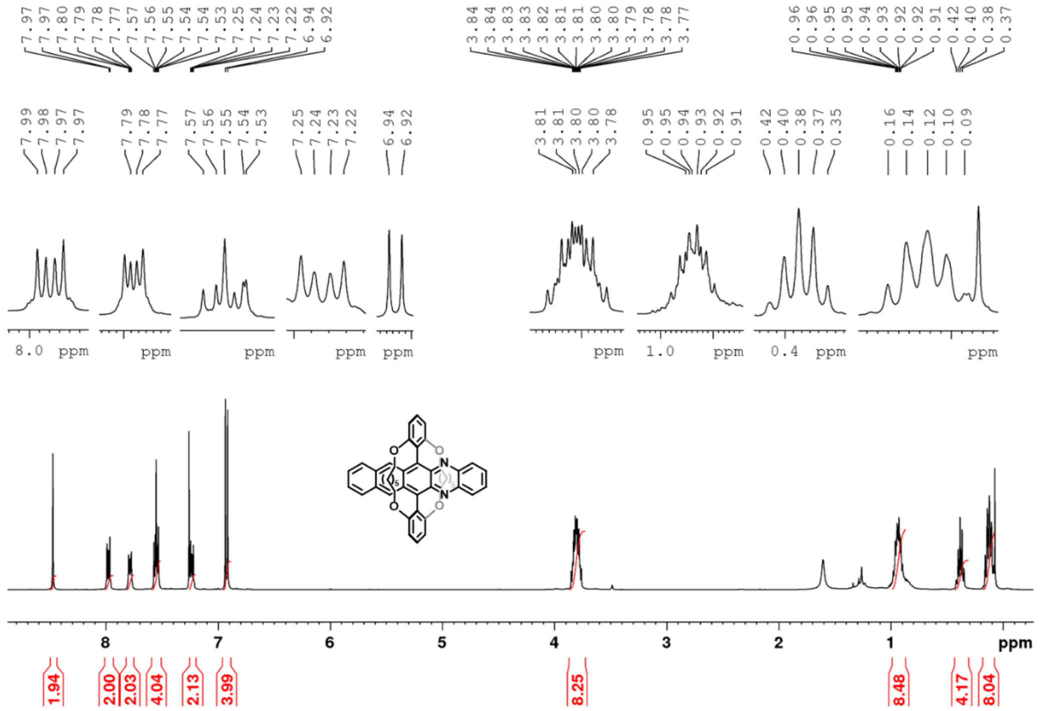

Figure S34: ^{1}H NMR spectrum (400 MHz) of **8** in $CDCl_3$.

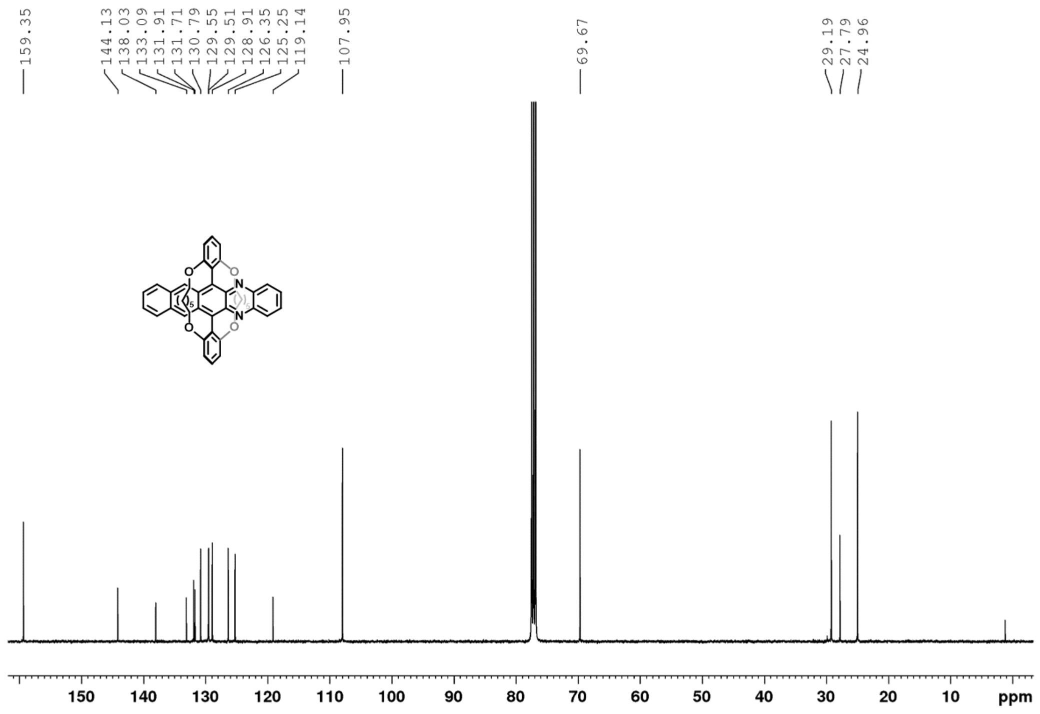

Figure S35: $^{13}C\{^1H\}$ NMR spectrum (101 MHz) of **8** in $CDCl_3$.

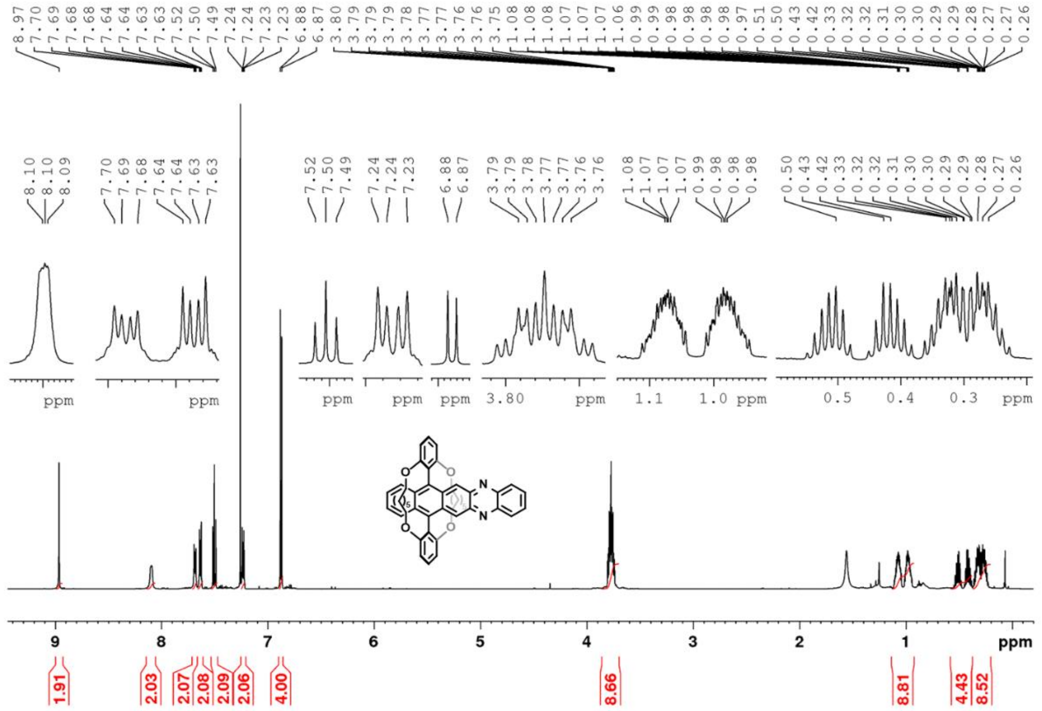

Figure S36: ^{1}H NMR spectrum (600 MHz) of **9** in $CDCl_3$.

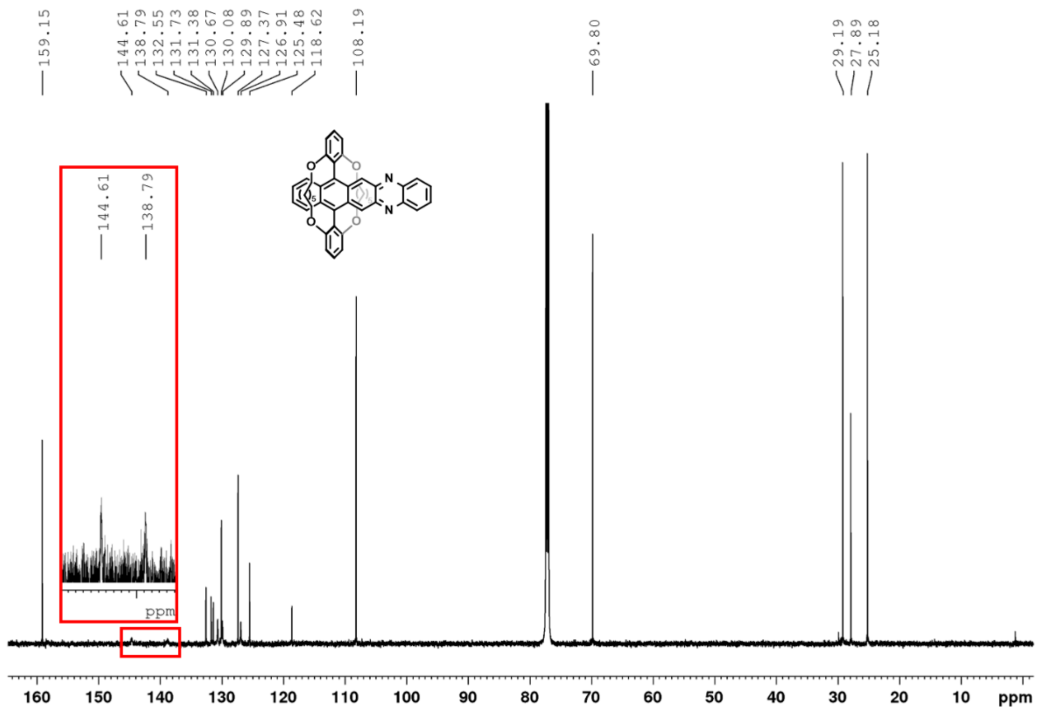

Figure S37: $^{13}C\{^{1}H\}$ NMR spectrum (151 MHz) of **9** in $CDCl_3$.

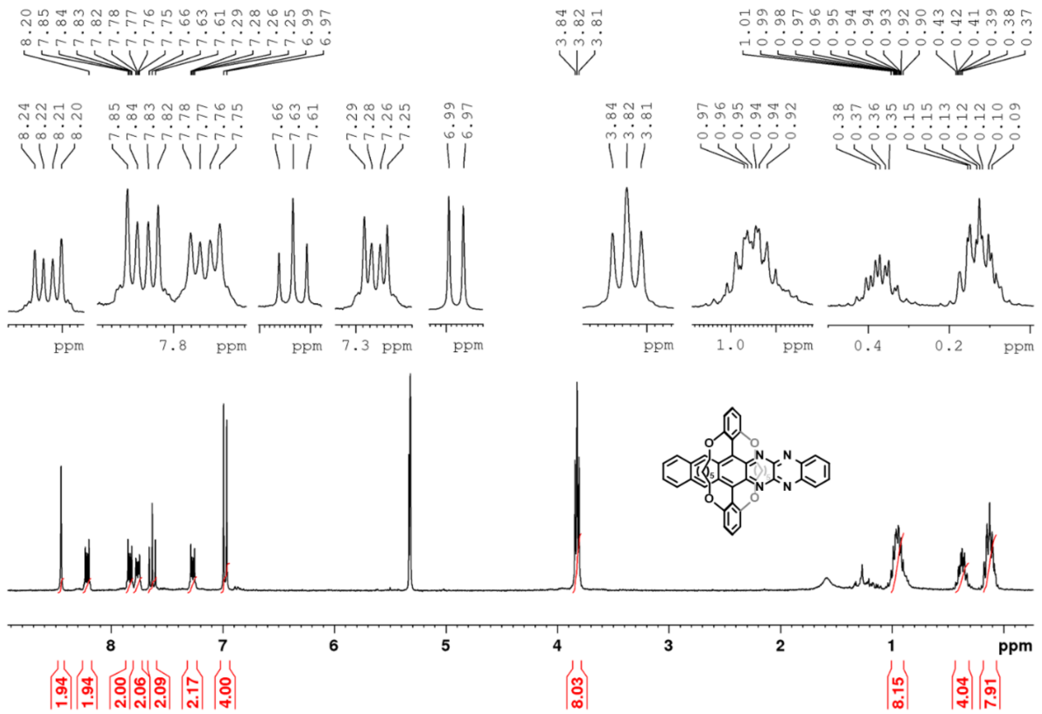

Figure S38: ^{1}H NMR spectrum (300 MHz) of **13** in CD_2Cl_2.

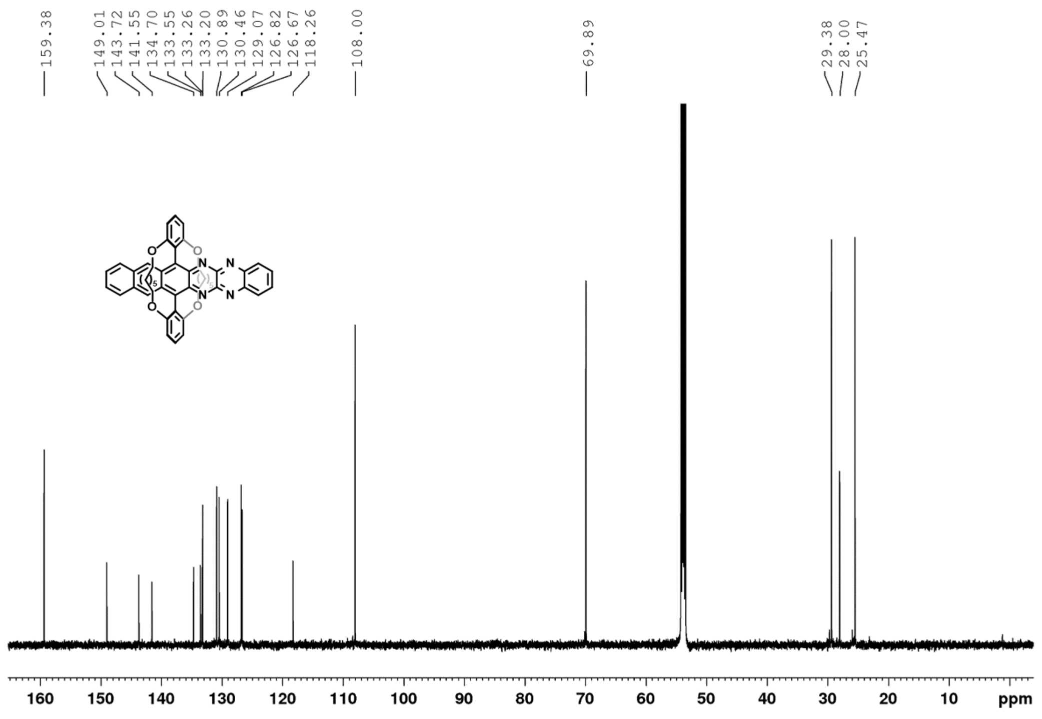

Figure S39: $^{13}C\{^{1}H\}$ NMR spectrum (151 MHz) of **13** in CD_2Cl_2.

2.3 UV/vis Stability Studies

All UV/vis stability studies were performed irradiating dilute solutions (10^{-5} mol L^{-1} in 3.00 mL dichloromethane, 99.8% spectroscopy grade from ACROS ORGANICS, stabilized with amylene, used as delivered) of the respective acene in quartz cuvettes at room temperature with a handheld UV lamp from HEROLAB GMBH LABORGERÄTE, Wiesloch (type: NU-15, λ_1 = 365 nm (15 W) and λ_2 = 254 nm (15 W)). The four-sided cuvette was placed in a distance of 5 cm (for studies performed under argon atmosphere) or 20 cm (for studies performed under ambient conditions) to the lamp, one side parallel to the lamp's front. For studies performed under argon atmosphere, prior to irradiation, argon was bubbled through the solution for at least 1 h.

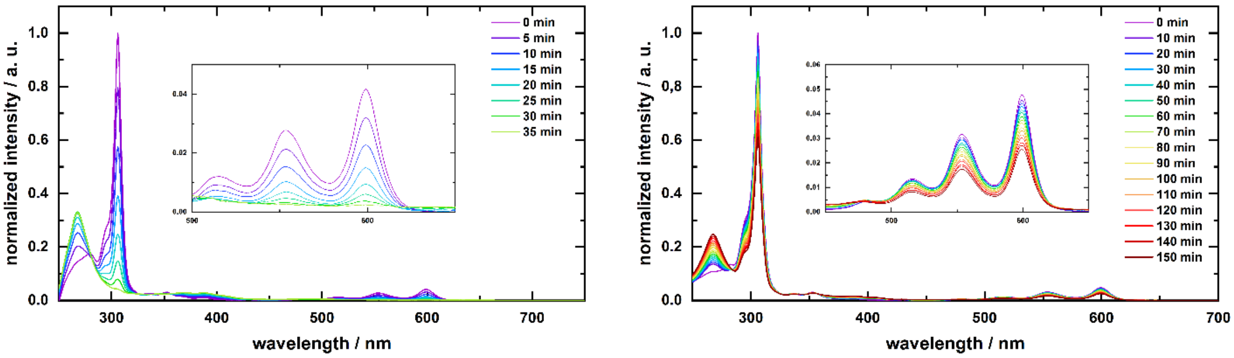

Figure S40. Change in absorption intensity of a solution of **5** (10^{-5} mol L^{-1}, quartz cuvette) while irradiating with a handheld UV lamp (λ_1 = 365 nm and λ_2 = 254 nm) in DCM at room temperature under ambient conditions (left) and under argon atmosphere (right).

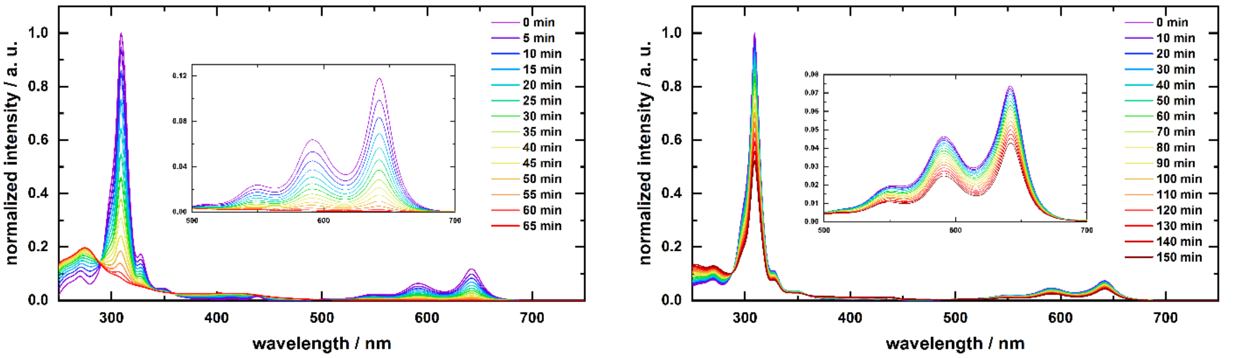

Figure S41. Change in absorption intensity of a solution of $\mathbf{5_{TIPS}}$ (10^{-5} mol L^{-1}, quartz cuvette) while irradiating with a handheld UV lamp (λ_1 = 365 nm and λ_2 = 254 nm) in DCM at room temperature under ambient conditions (left) and under argon atmosphere (right).

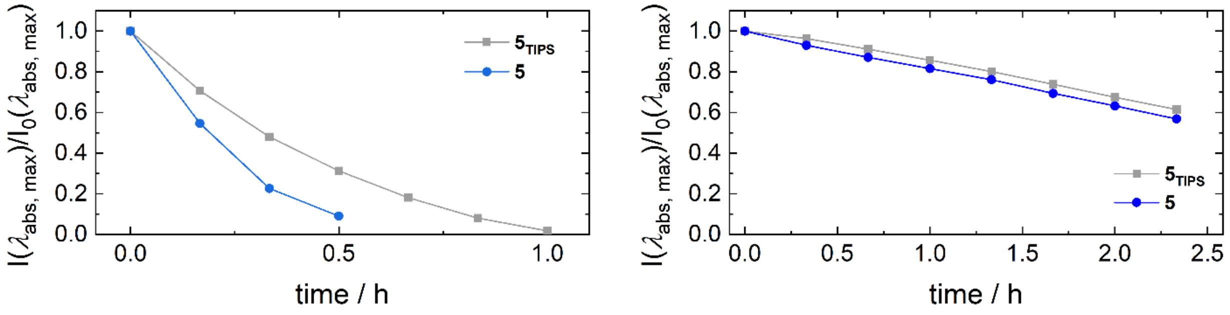

Figure S42. Change in UV/vis absorption intensity at $\lambda_{abs, max}$ for **5** and $\mathbf{5_{TIPS}}$ (10^{-5} mol L^{-1}, quartz cuvette) while irradiating with a handheld UV lamp (λ_1 = 365 nm and λ_2 = 254 nm) in DCM at room temperature under argon atmosphere (left) and under ambient conditions (right).

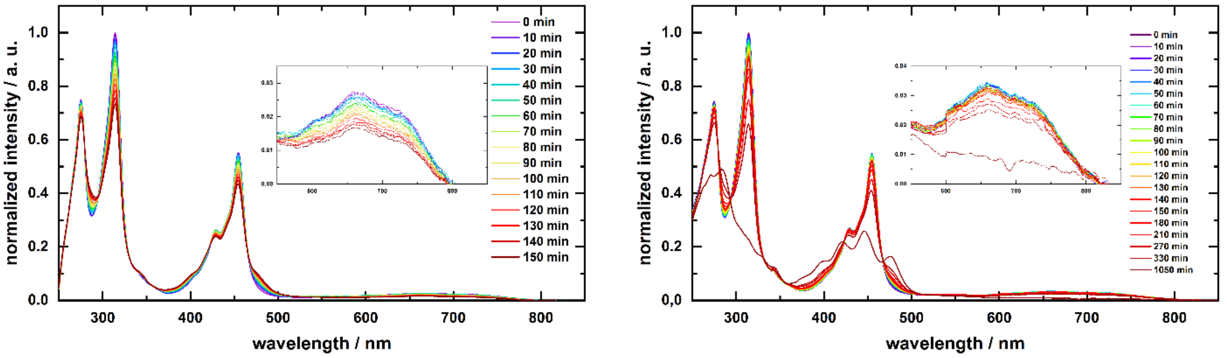

Figure S43. Change in absorption intensity of a solution of **6** (10^{-5} mol L^{-1}, quartz cuvette) after irradiation with a handheld UV lamp (λ_1 = 365 nm and λ_2 = 254 nm) in DCM at room temperature under ambient conditions (left) and under argon atmosphere (right).

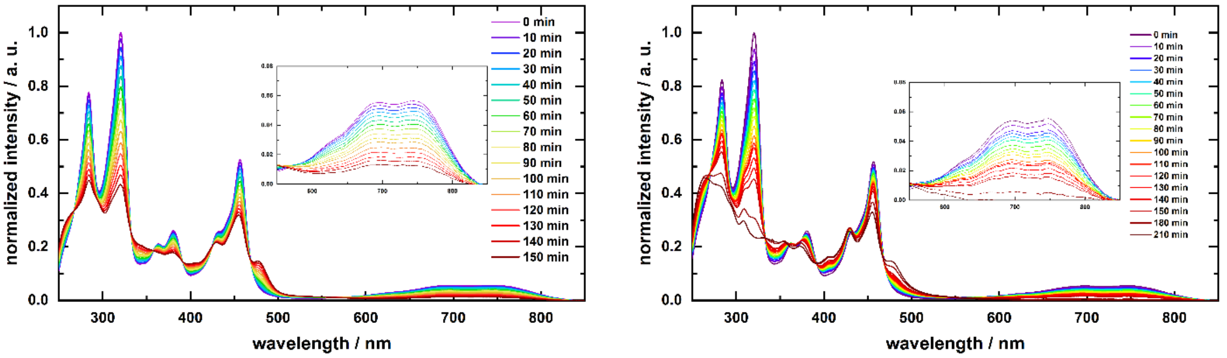

Figure S44. Change in absorption intensity of a solution of **6**$_{\mathbf{TIPS}}$ (10^{-5} mol L^{-1}, quartz cuvette) while irradiating with a handheld UV lamp (λ_1 = 365 nm and λ_2 = 254 nm) in DCM at room temperature under ambient conditions (left) and under argon atmosphere (right).

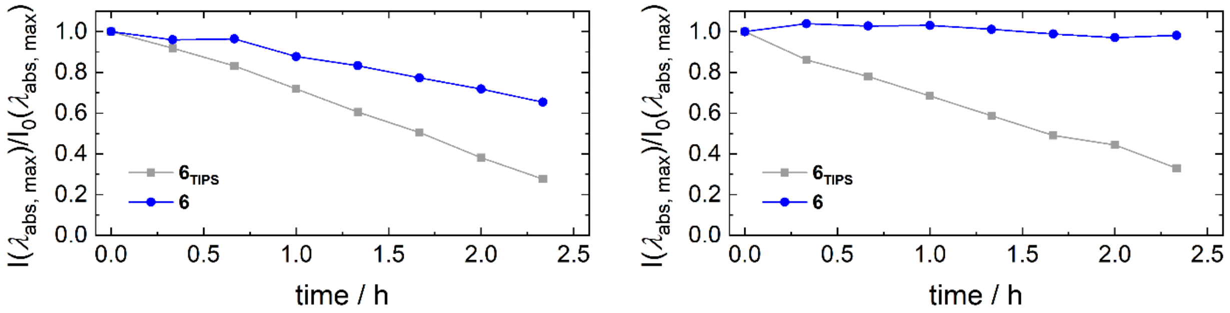

Figure S45. Change in UV/vis absorption intensity at $\lambda_{abs,\,max}$ for **6** and **6**$_{\mathbf{TIPS}}$ (10^{-5} mol L^{-1}, quartz cuvette) while irradiating with a handheld UV lamp (λ_1 = 365 nm and λ_2 = 254 nm) in DCM at room temperature under argon atmosphere (left) and under ambient conditions (right).

.

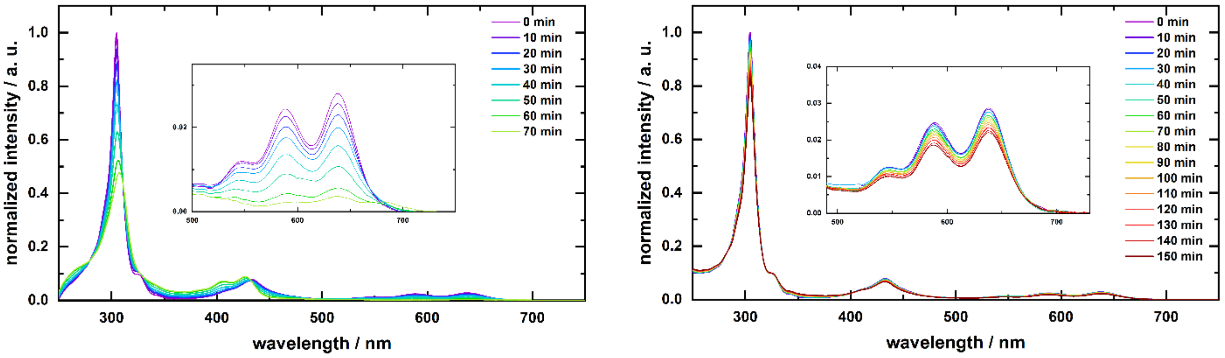

Figure S46. Change in absorption intensity of a solution of **7** (10^{-5} mol L^{-1}) while irradiating with a handheld UV lamp (λ_1 = 365 nm and λ_2 = 254 nm) in DCM at room temperature under ambient conditions (left) and under argon atmosphere (right).

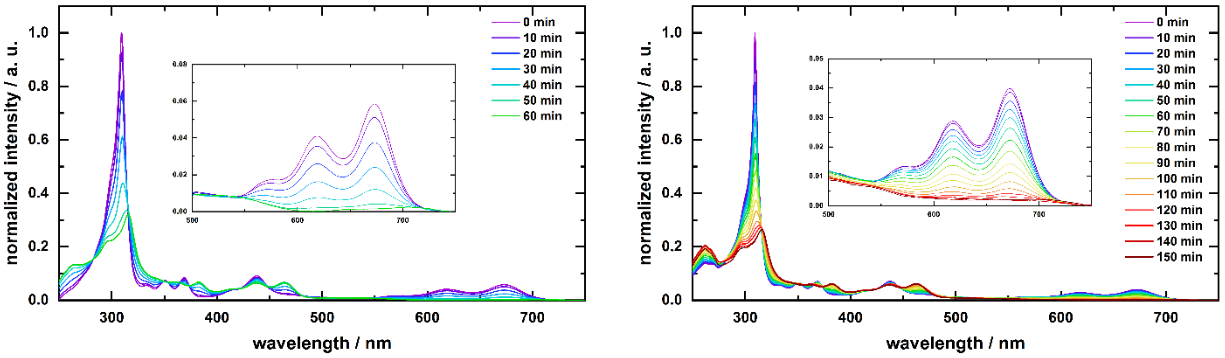

Figure S47. Change in absorption intensity of a solution of $\mathbf{7}_{\mathbf{TIPS}}$ (10^{-5} mol L^{-1}, quartz cuvette) while irradiating with a handheld UV lamp (λ_1 = 365 nm and λ_2 = 254 nm) in DCM at room temperature under ambient conditions (left) and under argon atmosphere (right).

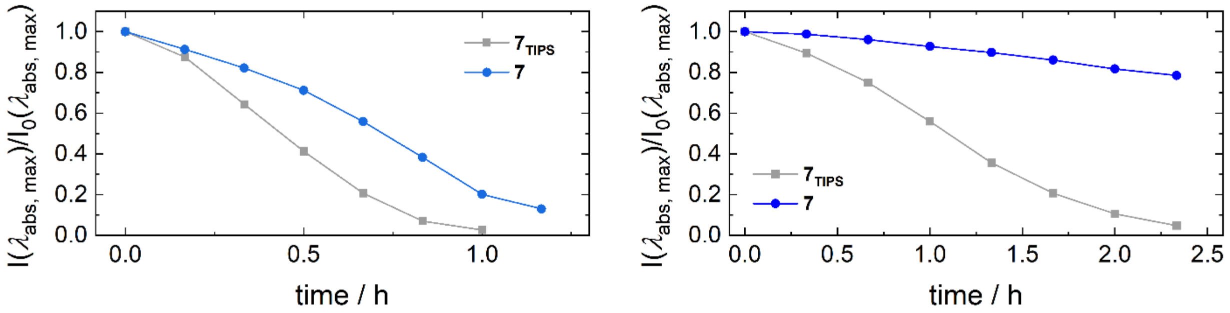

Figure S48. Change in UV/vis absorption intensity at $\lambda_{abs,\,max}$ for **7** and $\mathbf{7}_{\mathbf{TIPS}}$ (10^{-5} mol L^{-1}, quartz cuvette) while irradiating with a handheld UV lamp (λ_1 = 365 nm and λ_2 = 254 nm) in DCM at room temperature under argon atmosphere (left) and under ambient conditions (right).

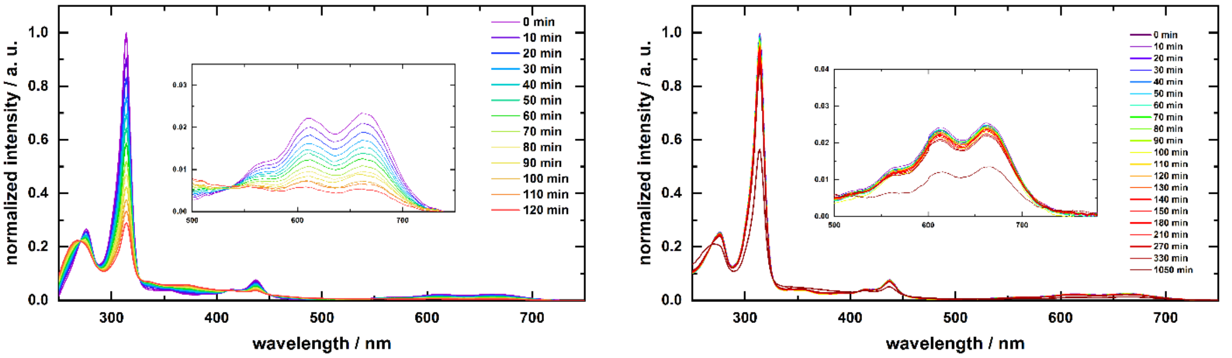

Figure S49. Change in absorption intensity of a solution of **8** (10^{-5} mol L^{-1}, quartz cuvette) while irradiating with a handheld UV lamp (λ_1 = 365 nm and λ_2 = 254 nm) in DCM at room temperature under ambient conditions (left) and under argon atmosphere (right).

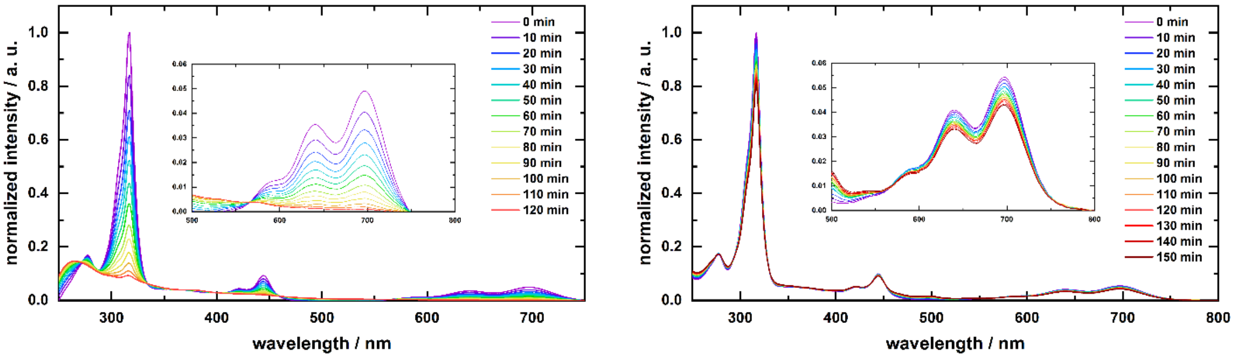

Figure S50. Change in absorption intensity of a solution of **8**$_{\mathbf{TIPS}}$ (10^{-5} mol L^{-1}, quartz cuvette) while irradiating with a handheld UV lamp (λ_1 = 365 nm and λ_2 = 254 nm) in DCM at room temperature under ambient conditions (left) and under argon atmosphere (right).

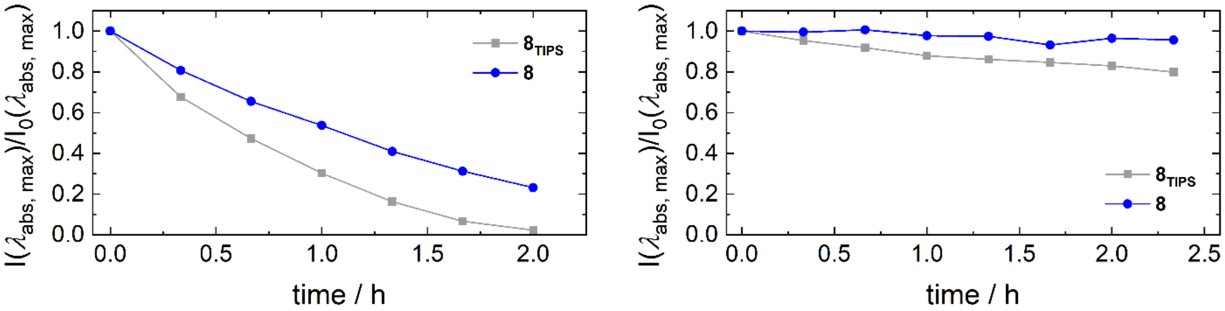

Figure S51. Change in UV/vis absorption intensity at $\lambda_{abs,\ max}$ for **8** and **8**$_{\mathbf{TIPS}}$ (10^{-5} mol L^{-1}, quartz cuvette) while irradiating with a handheld UV lamp (λ_1 = 365 nm and λ_2 = 254 nm) in DCM at room temperature under argon atmosphere (left) and under ambient conditions (right).

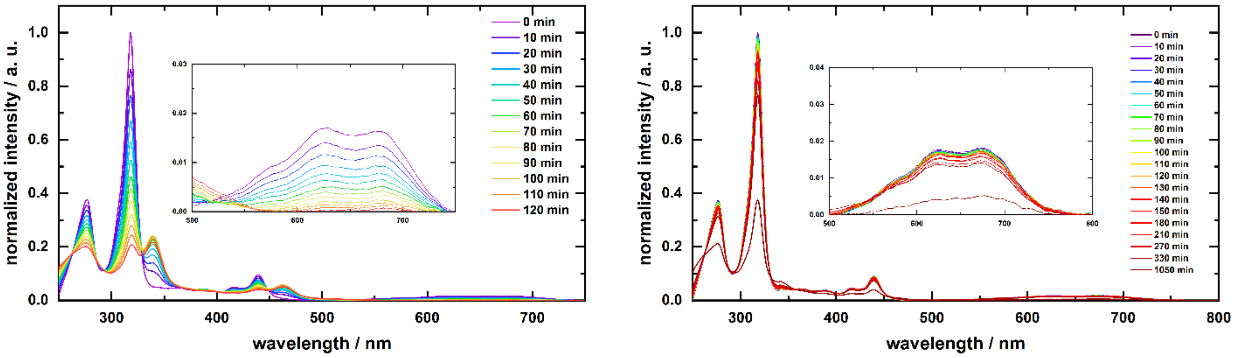

Figure S52. Change in absorption intensity of a solution of **9** (10^{-5} mol L^{-1}, quartz cuvette) while irradiating with a handheld UV lamp (λ_1 = 365 nm and λ_2 = 254 nm) in DCM at room temperature under ambient conditions (left) and under argon atmosphere (right).

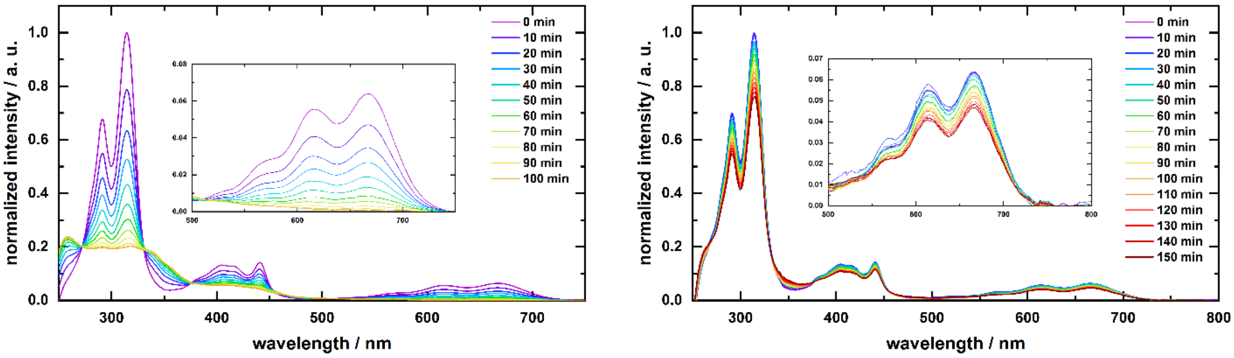

Figure S53. Change in absorption intensity of a solution of $\mathbf{9_{TIPS}}$ (10^{-5} mol L^{-1}, quartz cuvette) while irradiating with a handheld UV lamp (λ_1 = 365 nm and λ_2 = 254 nm) in DCM at room temperature under ambient conditions (left) and under argon atmosphere (right).

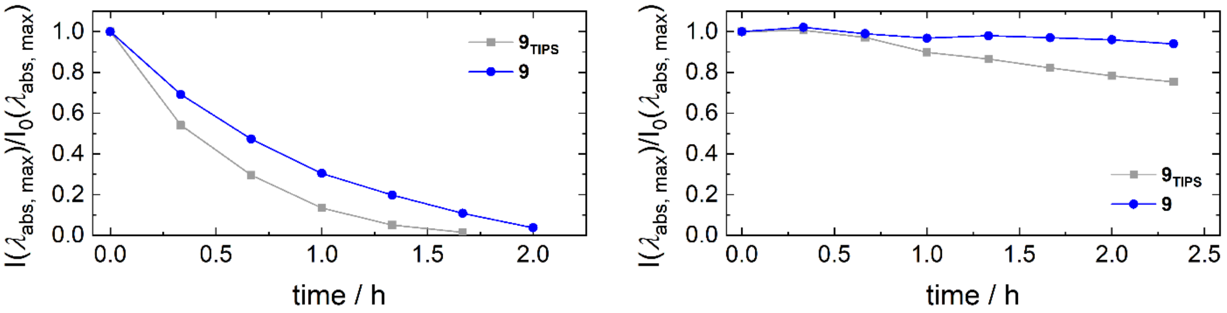

Figure S54. Change in UV/vis absorption intensity at $\lambda_{abs,\,max}$ for **9** and $\mathbf{9_{TIPS}}$ (10^{-5} mol L^{-1}, quartz cuvette) while irradiating with a handheld UV lamp (λ_1 = 365 nm and λ_2 = 254 nm) in DCM at room temperature under argon atmosphere (left) and under ambient conditions (right).

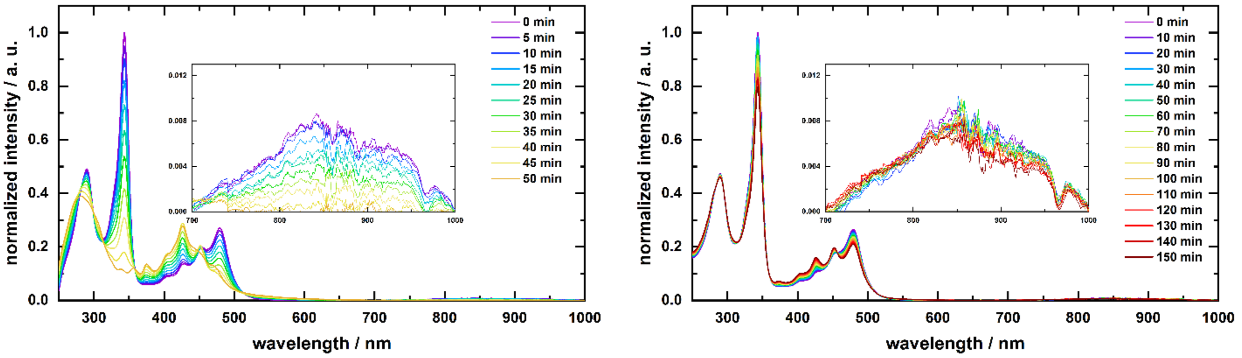

Figure S55. Change in absorption intensity of a solution of **13** (10^{-5} mol L^{-1}, quartz cuvette) while irradiating with a handheld UV lamp (λ_1 = 365 nm and λ_2 = 254 nm) in DCM at room temperature under ambient conditions (left) and under argon atmosphere (right).

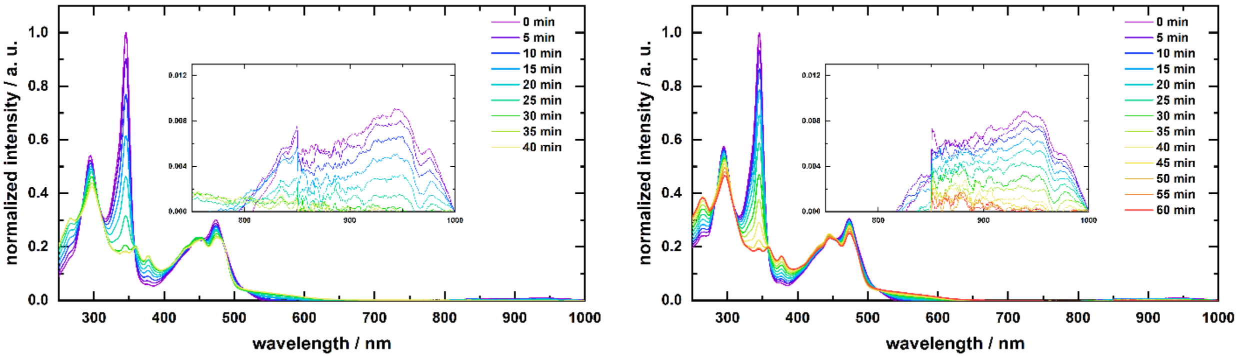

Figure S56. Change in absorption intensity of a solution of **13**$_{\text{TIPS}}$ (10^{-5} mol L^{-1}, quartz cuvette) while irradiating with a handheld UV lamp (λ_1 = 365 nm and λ_2 = 254 nm) in DCM at room temperature under ambient conditions (left) and under argon atmosphere (right).

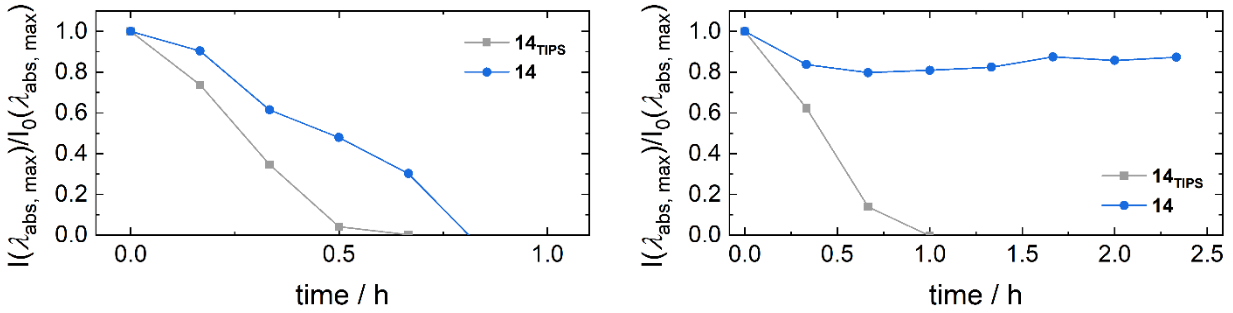

Figure S57. Change in UV/vis absorption intensity at $\lambda_{abs, max}$ for **13** and **13**$_{\text{TIPS}}$ (10^{-5} mol L^{-1}, quartz cuvette) while irradiating with a handheld UV lamp (λ_1 = 365 nm and λ_2 = 254 nm) in DCM at room temperature under argon atmosphere (left) and under ambient conditions (right).

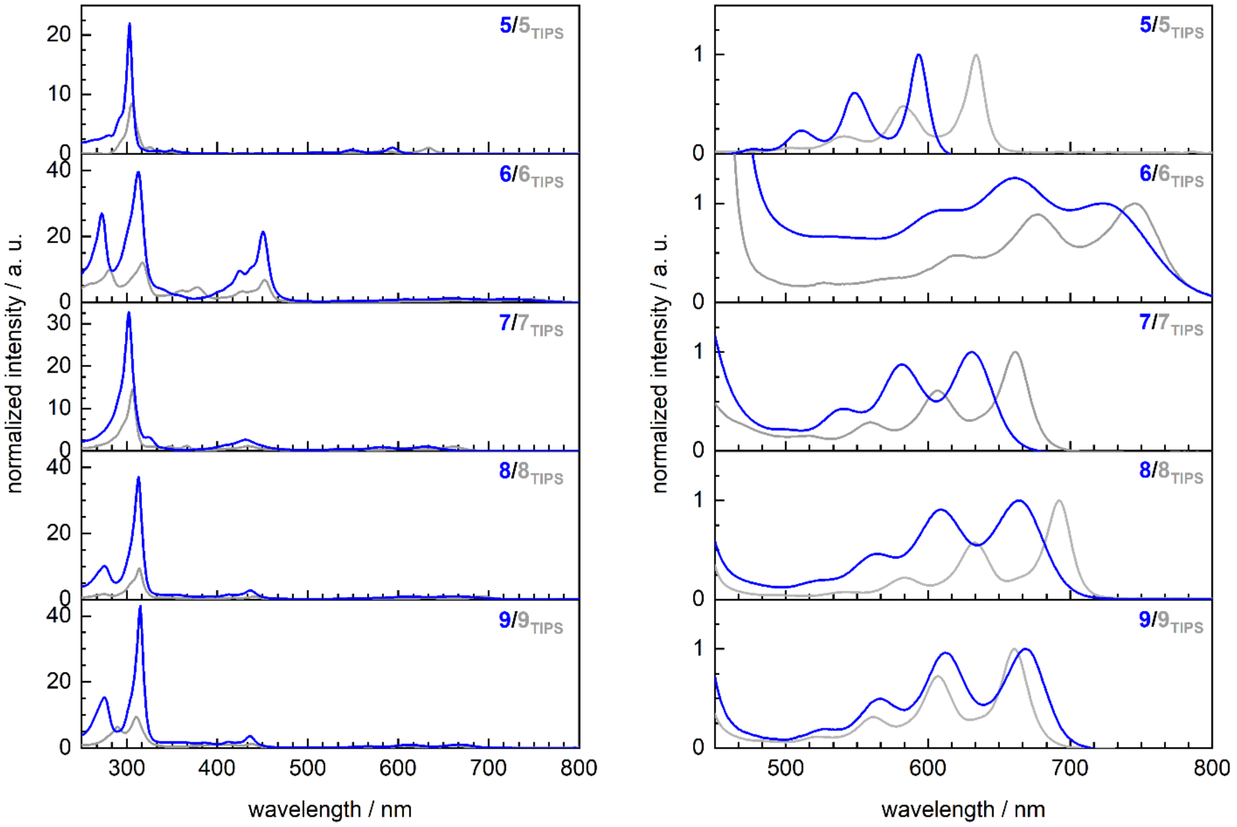

Figure S58. Normalized absorption spectra of double alkylene bridged (aza)pentacenes **5-9** and their consanguine silylated counterparts **5**$_{TIPS}$-**9**$_{TIPS}$ in dilute solution (*n*-hexane).

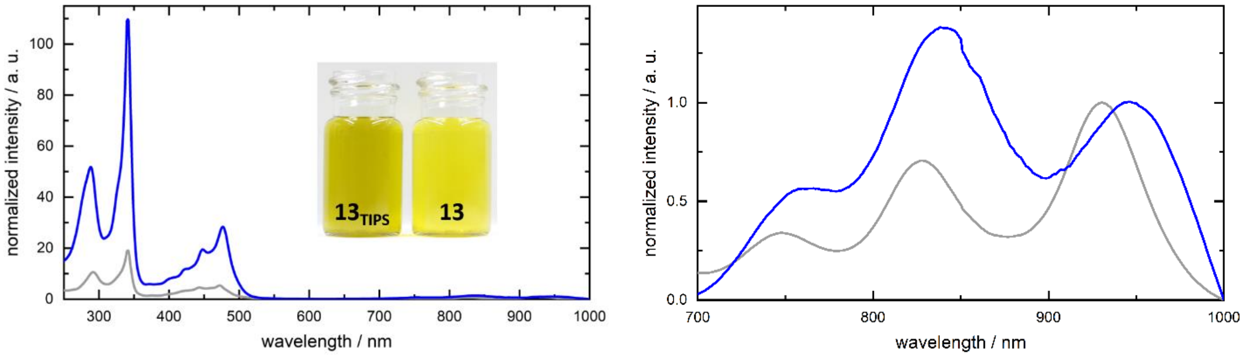

Figure S59. Normalized absorption spectra of double alkylene-strapped hexacene **13** and its consanguine silylated counterpart **13**$_{TIPS}$ in dilute solution (*n*-hexane). Insert: Photographs of double alkylene-strapped azahexacenes **13** and its consanguine silylated counterpart **13**$_{TIPS}$ under daylight in *n*-hexane.

2.4 Cyclic Voltammetry

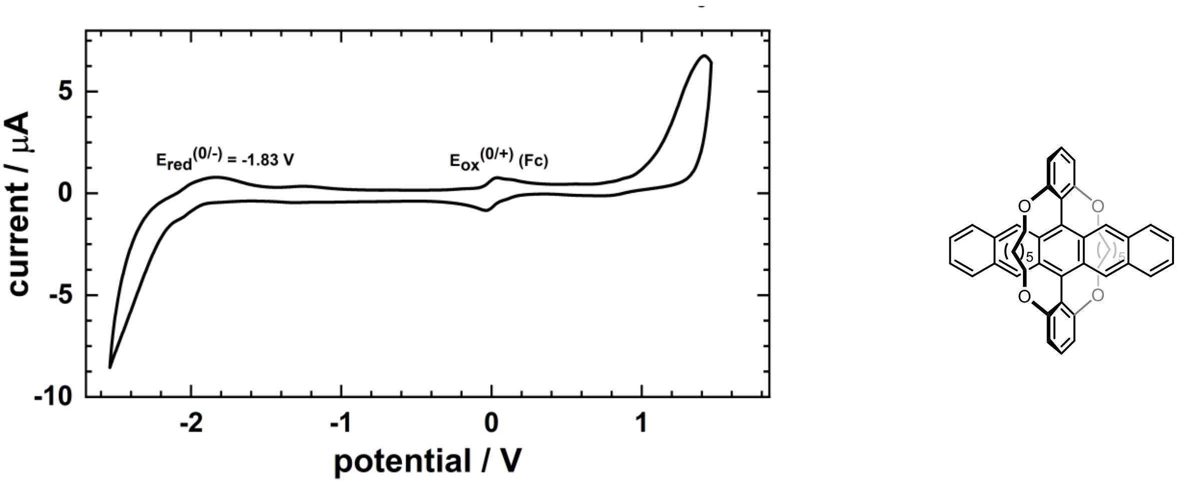

Figure S60. Cyclic voltammogram of **5** in CH_2Cl_2 using Bu_4NPF_6 as electrolyte and Fc/Fc^+ as internal standard with a scan speed of 0.2 Vs^{-1}.

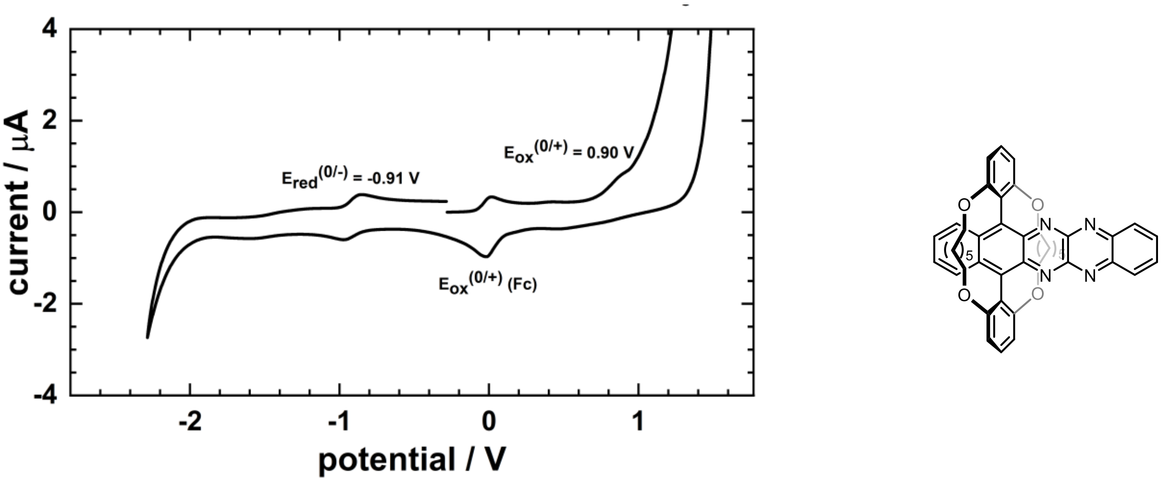

Figure S61. Cyclic voltammogram of **6** in CH_2Cl_2 using Bu_4NPF_6 as electrolyte and Fc/Fc^+ as internal standard with a scan speed of 0.2 Vs^{-1}.

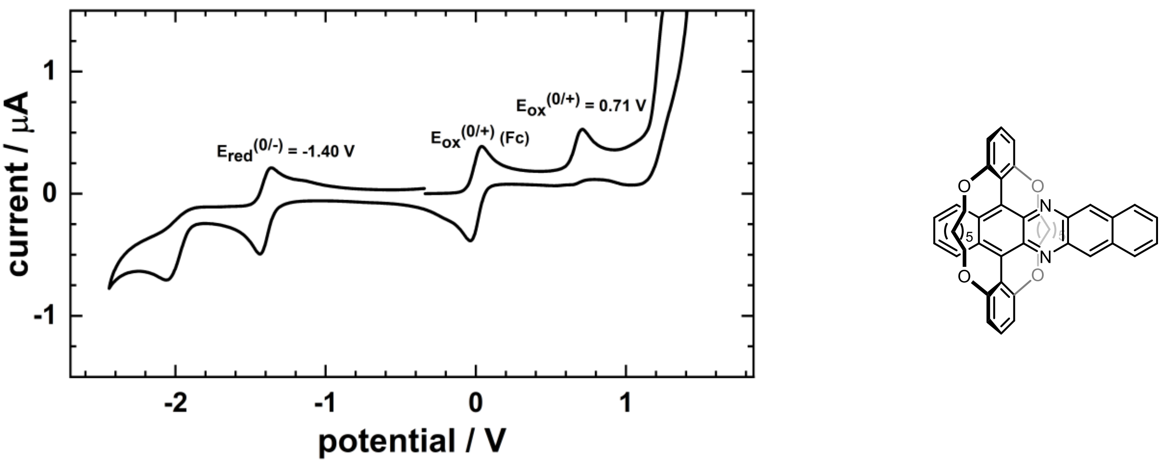

Figure S62. Cyclic voltammogram of **7** in CH_2Cl_2 using Bu_4NPF_6 as electrolyte and Fc/Fc^+ as internal standard with a scan speed of 0.2 Vs^{-1}.

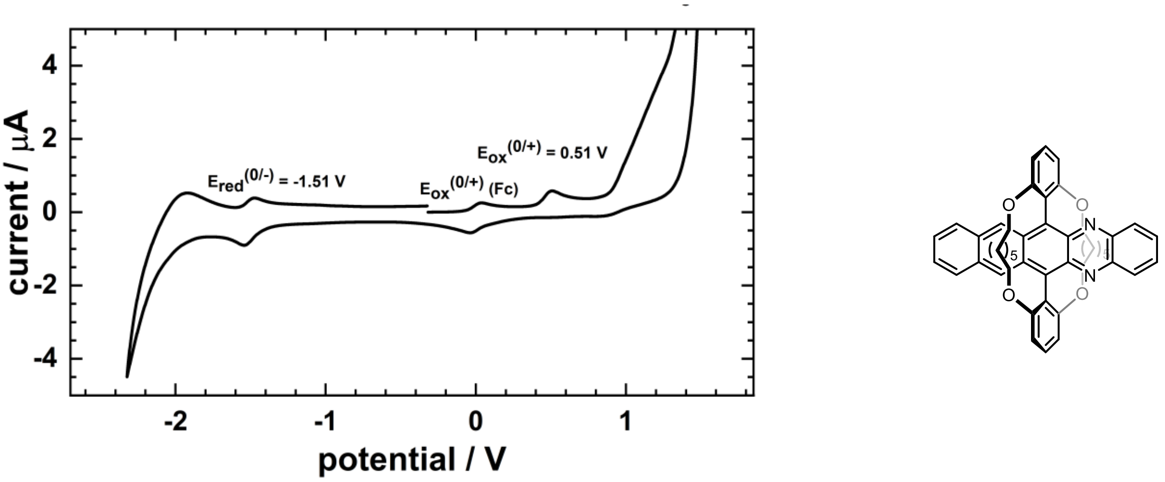

Figure S63. Cyclic voltammogram of **8** in CH_2Cl_2 using Bu_4NPF_6 as electrolyte and Fc/Fc^+ as internal standard with a scan speed of 0.2 Vs^{-1}.

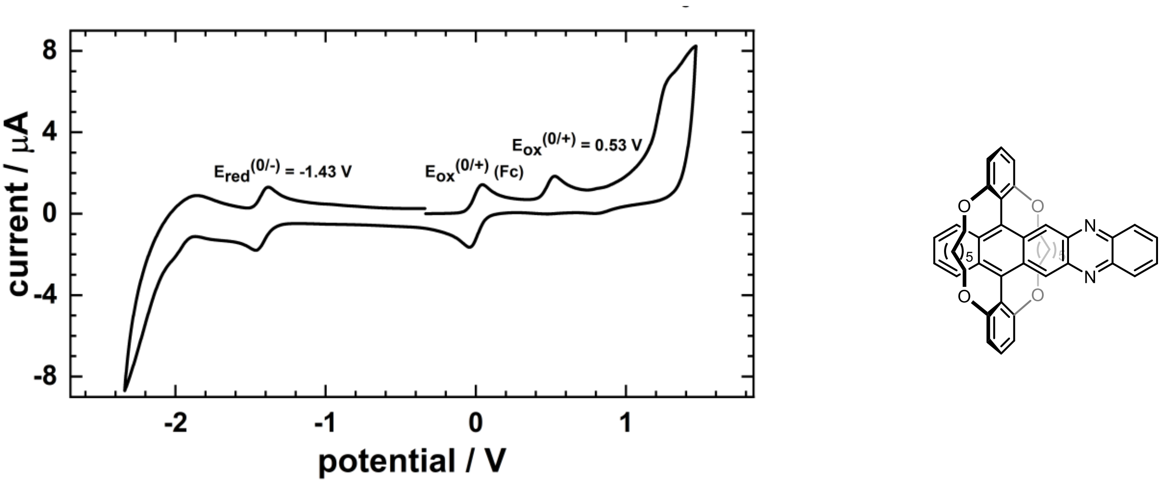

Figure S64. Cyclic voltammogram of **9** in CH_2Cl_2 using Bu_4NPF_6 as electrolyte and Fc/Fc^+ as internal standard with a scan speed of 0.2 Vs^{-1}.

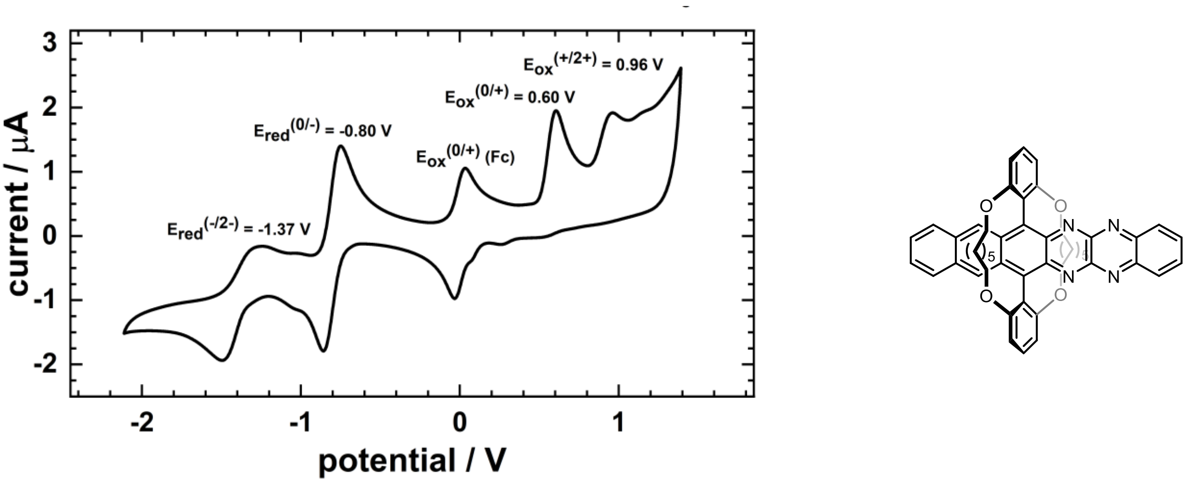

Figure S65. Cyclic voltammogram of **13** in CH_2Cl_2 using Bu_4NPF_6 as electrolyte and Fc/Fc^+ as internal standard at 0.2 Vs^{-1}.

Table S1. Experimental and calculated (gas-phase) properties of double alkylene-strapped pentacenes **5-9** and hexacene **13** and their consanguine TIPS analogs in solution (UV-vis: *n*-hexane; CV: DCM).

Compd	$E^{(0/-)}$ [V][a]	Ionization Potential/ HOMO [eV] [d]meas./[e]calcd	Electron Affinity/ LUMO [eV] [b]meas./[e]calcd	gap [eV] [c]meas./[e]calcd	$\lambda_{max,\ abs}$ [nm]	$\lambda_{onset,\ abs}$ [nm]	$\lambda_{max,\ em}$ [nm]	Stokes shift [cm^{-1}]
**5**	−1.83	−5.30/−4.74	−3.27/−2.63	2.03/2.10	593	610	599	169
**5$_{TIPS}$**[f]	-[g]	-5.40/−4.92	-3.53/−3.02	1.92/1.90	634	646	637	74
**6**	−0.91	−5.79/−5.53	−4.19/−3.48	1.60/2.01	723	776	-	-
**6$_{TIPS}$**[f]	−0.52	−6.15/−5.64	−4.58/−3.86	1.57/1.77	745	789	750	90
**7**	−1.40	−5.57/−5.28	−3.70/−3.17	1.87/2.11	631	663	652	510
**7$_{TIPS}$**[f]	−0.97	−5.94/−5.37	−4.13/−3.45	1.81/1.92	661	685	670	203
**8**	−1.51	−5.33/−4.94	−3.59/−2.89	1.74/2.05	664	711	687	527
**8$_{TIPS}$**[f]	−1.08	−5.76/−5.24	−4.02/−3.42	1.74/1.82	692	714	698	124
**9**	−1.43	−5.44/−5.02	−3.67 /−2.97	1.77/2.05	668	701	685	372
**9$_{TIPS}$**	−1.16	−5.76/−5.27	−3.94/−3.37	1.82/1.91	661	683	671	226
**13**	-0.80	-5.54/-5.16	-4.30/-3.70	1.24/1.47	946	997	-[h]	-
**13$_{TIPS}$**[f]	-0.58	-5.79/-5.31	-4.52/-3.96	1.27/1.36	930	974	-[h]	-

[a] First reduction potentials from cyclic voltammetry (CV) in DCM at room temperature with Bu_4NPF_6 as the electrolyte against Fc/Fc^+ as an internal standard (−5.10 eV) at 0.2 V/s;[S9] [b] electron affinity$_{meas.}$= -e×(5.1 V+$E^{(0/-)}$); [c] gap$_{meas.}$ calculated from λ_{onset} in *n*-hexane; [d] ionization potential$_{meas.}$= electron affinity$_{meas}$ - gap$_{meas}$; [e] obtained from DFT calculations (Gaussian16 B3LYP/ def2-SVP// Gaussian16 B3LYP/ def2-TZVP; TMS groups were used instead of TIPS); [f] data for **5$_{TIPS}$** was taken from [S10], for **6$_{TIPS}$** (ACN) from [S7b], for **7$_{TIPS}$** from [S7c], for **8$_{TIPS}$** (THF) from [S7d], for **9$_{TIPS}$** from [S7e] and for **13$_{TIPS}$** from [S8]; [g] EA and IP derived from $E^{(0/+)}$; [h] non-emissive.

2.5 Crystallographic Data

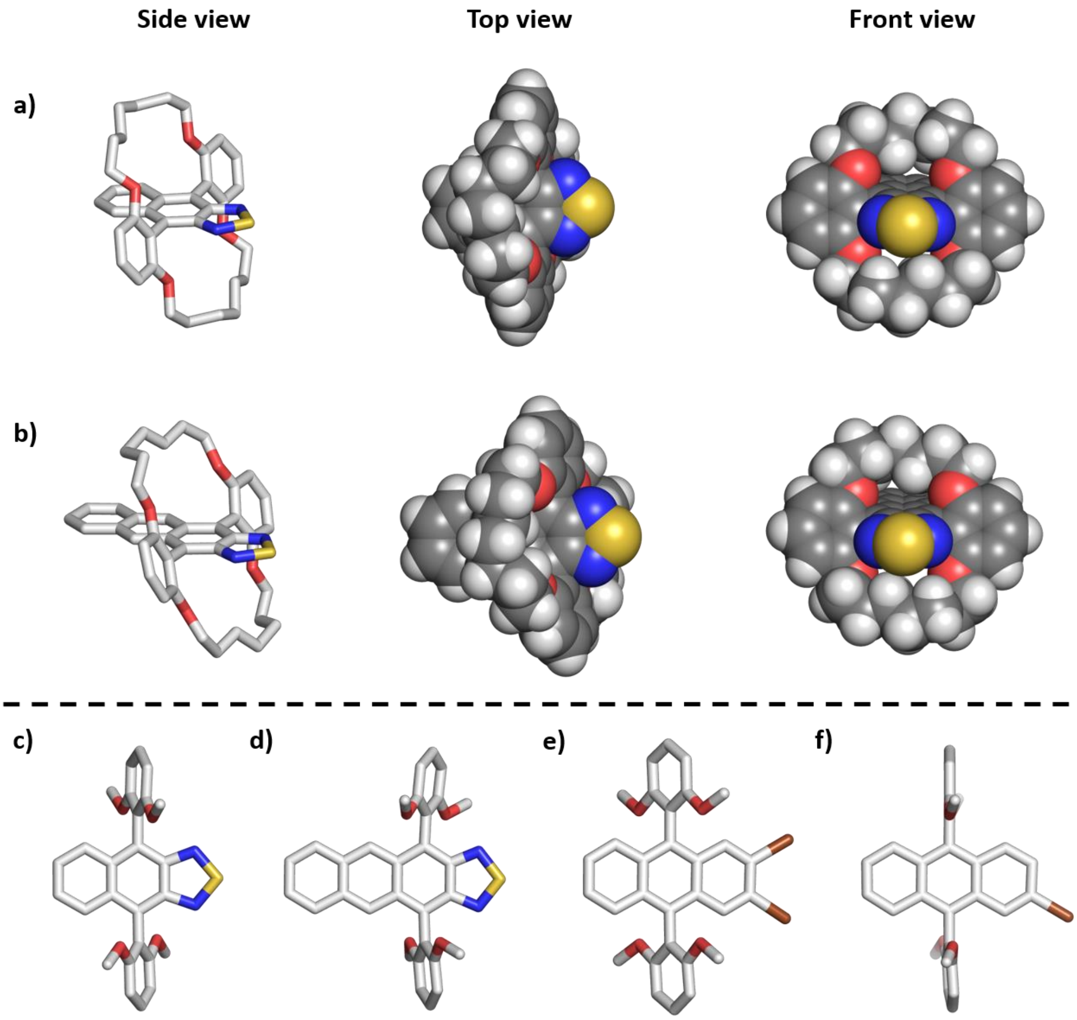

Figure S66: Solid state structures of **S7** (a), **S8** (b), their respective precursors **S3** (c), **S4** (d) and **S10** (e). In an attempt to grow crystals of **S10**, a subhalogenated side-product 2-bromo-9,10-bis(2,6-dimethoxyphenyl)anthracene (f) was also analyzed.

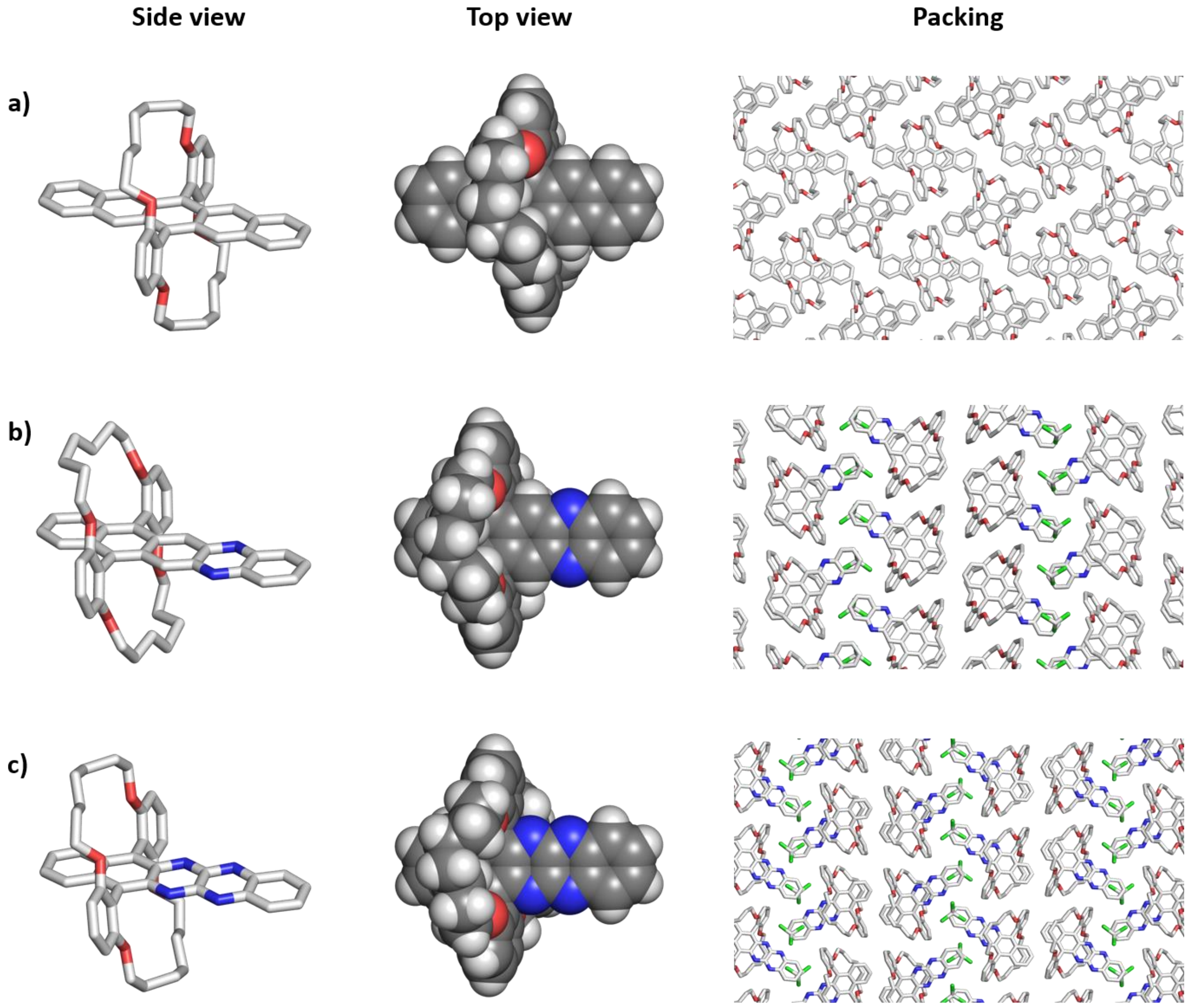

Figure S67: Solid state structures and crystal packing of azapentacenes **5** (a), **9** (b) and **6** (c).

.

Table S2. Crystal structure, crystal data and structure refinement of **5** (CCDC 2044179).

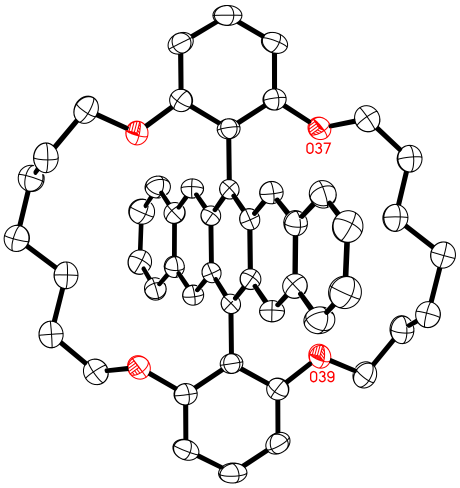

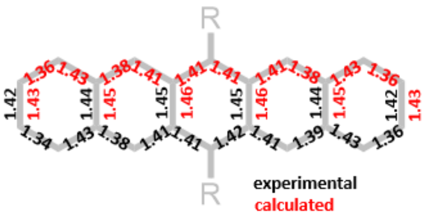

Identification code	CCDC 2044179	
Empirical formula	$C_{48}H_{46}O_4$	
Formula weight	686.85	
Temperature	200(2) K	
Wavelength	1.54178 Å	
Crystal system	triclinic	
Space group	P $\bar{1}$	
Z	2	
Unit cell dimensions	a = 8.9960(4) Å	α = 92.178(3) deg.
	b = 13.3013(5) Å	β = 91.838(3) deg.
	c = 15.4926(6) Å	γ = 94.562(3) deg.
Volume	1845.47(13) Å^3	
Density (calculated)	1.24 g/cm^3	
Absorption coefficient	0.60 mm^{-1}	
Crystal shape	plate	
Crystal size	0.140 x 0.105 x 0.025 mm^3	
Crystal colour	purple	
Theta range for data collection	2.9 to 72.1 deg.	
Index ranges	$-10 \le h \le 9$, $-16 \le k \le 10$, $-17 \le l \le 19$	
Reflections collected	19660	
Independent reflections	6907 (R(int) = 0.0459)	
Observed reflections	3971 ($I > 2\sigma(I)$)	
Absorption correction	Semi-empirical from equivalents	
Max. and min. transmission	1.59 and 0.54	
Refinement method	Full-matrix least-squares on F^2	
Data/restraints/parameters	6907 / 235 / 524	
Goodness-of-fit on F^2	0.98	
Final R indices (I>2sigma(I))	R1 = 0.051, wR2 = 0.124	
Largest diff. peak and hole	0.34 and -0.23 eÅ^{-3}	

Table S3. Crystal structure, crystal data and structure refinement of **9** (CCDC 2044181).

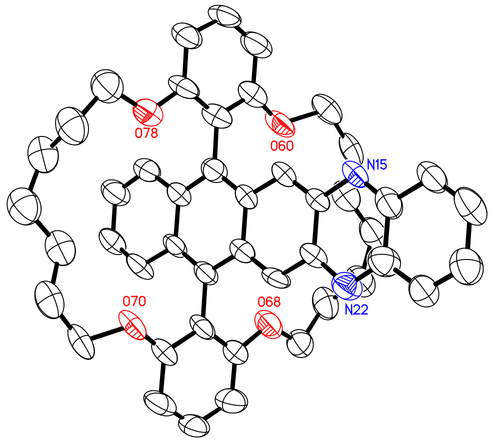

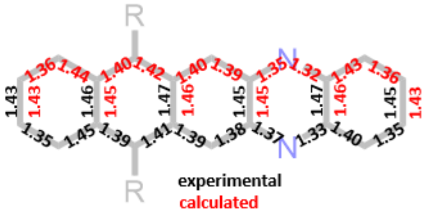

Identification code	CCDC 2044181	
Empirical formula	$C_{47}H_{45}Cl_3N_2O_4$	
Formula weight	808.20	
Temperature	200(2) K	
Wavelength	1.54178 Å	
Crystal system	monoclinic	
Space group	$P2_1/n$	
Z	4	
Unit cell dimensions	a = 8.3936(8) Å	α = 90 deg.
	b = 44.727(3) Å	β = 102.143(6) deg.
	c = 10.8552(8) Å	γ = 90 deg.
Volume	3984.0(6) $Å^3$	
Density (calculated)	1.35 g/cm^3	
Absorption coefficient	2.46 mm^{-1}	
Crystal shape	plate	
Crystal size	0.200 x 0.050 x 0.015 mm^3	
Crystal colour	green	
Theta range for data collection	4.0 to 46.1 deg.	
Index ranges	$-6 \le h \le 7$, $-41 \le k \le 41$, $-9 \le l \le 10$	
Reflections collected	11792	
Independent reflections	3320 (R(int) = 0.1092)	
Observed reflections	1571 ($I > 2\sigma(I)$)	
Absorption correction	Semi-empirical from equivalents	
Max. and min. transmission	2.92 and 0.41	
Refinement method	Full-matrix least-squares on F^2	
Data/restraints/parameters	3320 / 586 / 505	
Goodness-of-fit on F^2	0.95	
Final R indices (I>2sigma(I))	R1 = 0.091, wR2 = 0.215	
Largest diff. peak and hole	0.63 and -0.33 $eÅ^{-3}$	

Table S4. Crystal structure, crystal data and structure refinement of **6** (CCDC 2044180).

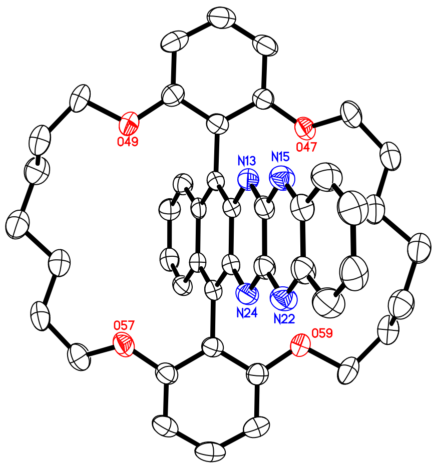

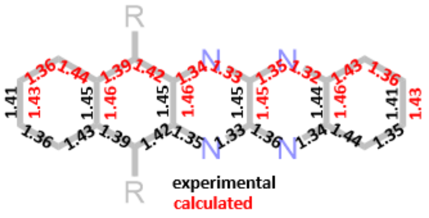

Identification code	CCDC 2044180	
Empirical formula	$C_{45}H_{43}Cl_3N_4O_4$	
Formula weight	810.18	
Temperature	200(2) K	
Wavelength	1.54178 Å	
Crystal system	monoclinic	
Space group	$P2_1/c$	
Z	4	
Unit cell dimensions	a = 8.6079(4) Å	α = 90 deg.
	b = 10.8329(5) Å	β = 92.120(4) deg.
	c = 42.478(2) Å	γ = 90 deg.
Volume	3958.3(3) $Å^3$	
Density (calculated)	1.36 g/cm^3	
Absorption coefficient	2.50 mm^{-1}	
Crystal shape	plank	
Crystal size	0.147 x 0.035 x 0.015 mm^3	
Crystal colour	green	
Theta range for data collection	5.1 to 67.1 deg.	
Index ranges	$-4 \le h \le 10$, $-12 \le k \le 11$, $-49 \le l \le 50$	
Reflections collected	16810	
Independent reflections	6374 (R(int) = 0.0351)	
Observed reflections	3862 ($I > 2\sigma(I)$)	
Absorption correction	Semi-empirical from equivalents	
Max. and min. transmission	1.28 and 0.72	
Refinement method	Full-matrix least-squares on F^2	
Data/restraints/parameters	6374 / 0 / 505	
Goodness-of-fit on F^2	1.02	
Final R indices (I>2sigma(I))	R1 = 0.057, wR2 = 0.125	
Largest diff. peak and hole	0.37 and -0.51 $eÅ^{-3}$	

Table S5. Crystal structure, crystal data and structure refinement of **13** (CCDC 2044184).

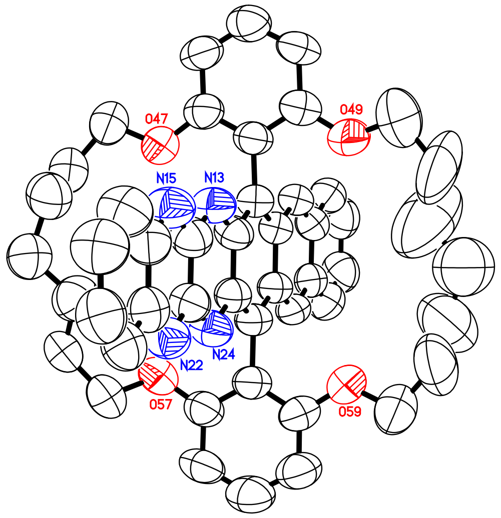

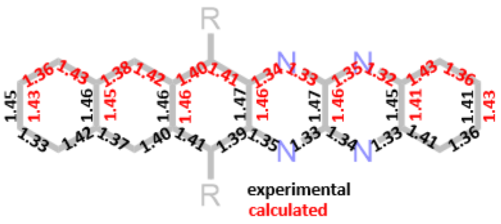

Identification code	CCDC 2044184	
Empirical formula	$C_{49}H_{45}Cl_3N_4O_4$	
Formula weight	860.24	
Temperature	200(2) K	
Wavelength	1.54178 Å	
Crystal system	monoclinic	
Space group	$P2_1/n$	
Z	4	
Unit cell dimensions	a = 11.0098(7) Å	α = 90 deg.
	b = 21.2505(11) Å	β =98.296(6) deg.
	c = 19.8994(14) Å	γ = 90 deg.
Volume	4607.0(5) $Å^3$	
Density (calculated)	1.24 g/cm^3	
Absorption coefficient	2.18 mm^{-1}	
Crystal shape	plate	
Crystal size	0.200 x 0.062 x 0.033 mm^3	
Crystal colour	green/brown	
Theta range for data collection	3.1 to 45.5 deg.	
Index ranges	$-8 \le h \le 10$, $-19 \le k \le 17$, $-18 \le l \le 18$	
Reflections collected	16226	
Independent reflections	3808 (R(int) = 0.0854)	
Observed reflections	2017 ($I > 2\sigma(I)$)	
Absorption correction	Semi-empirical from equivalents	
Max. and min. transmission	1.84 and 0.46	
Refinement method	Full-matrix least-squares on F^2	
Data/restraints/parameters	3808 / 631 / 541	
Goodness-of-fit on F^2	0.99	
Final R indices (I>2sigma(I))	R1 = 0.093, wR2 = 0.245	
Largest diff. peak and hole	0.43 and -0.23 $eÅ^{-3}$	

Table S6. Crystal structure, crystal data and structure refinement of **10** (CCDC 2044182).

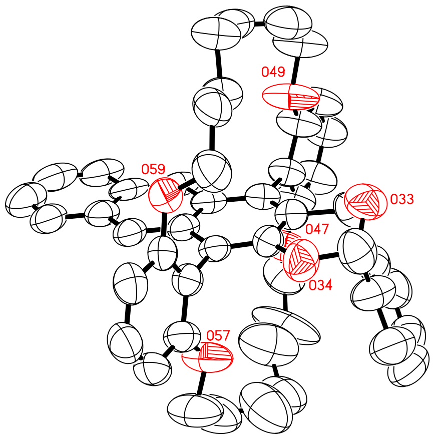

Identification code	CCDC 2044182		
Empirical formula	$C_{48}H_{46}O_6$		
Formula weight	718.85		
Temperature	200(2) K		
Wavelength	1.54178 Å		
Crystal system	orthorhombic		
Space group	Pbca		
Z	8		
Unit cell dimensions	a = 13.1467(7) Å	α =	90 deg.
	b = 15.9964(7) Å	β =	90 deg.
	c = 36.0630(13) Å	γ =	90 deg.
Volume	7584.0(6) $Å^3$		
Density (calculated)	1.26 g/cm^3		
Absorption coefficient	0.65 mm^{-1}		
Crystal shape	brick		
Crystal size	0.145 x 0.105 x 0.075 mm^3		
Crystal colour	violet		
Theta range for data collection	4.5 to 52.6 deg.		
Index ranges	$-13 \le h \le 13$, $-16 \le k \le 11$, $-32 \le l \le 37$		
Reflections collected	20516		
Independent reflections	4335 (R(int) = 0.0418)		
Observed reflections	2684 ($I > 2\sigma(I)$)		
Absorption correction	Semi-empirical from equivalents		
Max. and min. transmission	1.40 and 0.53		
Refinement method	Full-matrix least-squares on F^2		
Data/restraints/parameters	4335 / 558 / 487		
Goodness-of-fit on F^2	1.05		
Final R indices (I>2sigma(I))	R1 = 0.102, wR2 = 0.295		
Largest diff. peak and hole	0.61 and -0.33 $eÅ^{-3}$		

Table S7. Crystal structure, crystal data and structure refinement of **11** (CCDC 2044183).

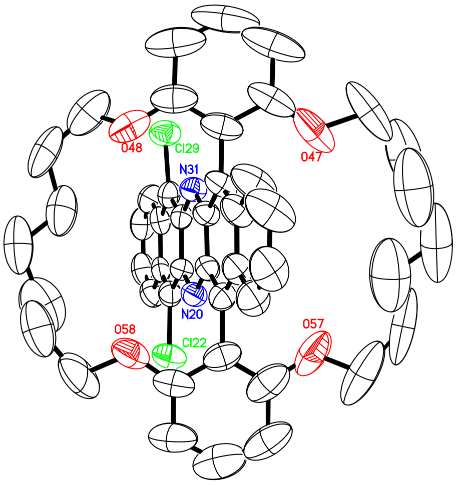

Identification code	CCDC 2044183	
Empirical formula	$C_{47}H_{43}Cl_5N_2O_4$	
Formula weight	877.08	
Temperature	200(2) K	
Wavelength	1.54178 Å	
Crystal system	triclinic	
Space group	$P\bar{1}$	
Z	2	
Unit cell dimensions	a = 10.5083(5) Å	α = 78.795(4) deg.
	b = 11.8968(6) Å	β = 76.419(4) deg.
	c = 18.9390(10) Å	γ = 64.321(4) deg.
Volume	2062.5(2) $Å^3$	
Density (calculated)	1.41 g/cm^3	
Absorption coefficient	3.59 mm^{-1}	
Crystal shape	plate	
Crystal size	0.110 x 0.080 x 0.018 mm^3	
Crystal colour	brown	
Theta range for data collection	4.1 to 52.6 deg.	
Index ranges	$-10 \le h \le 10$, $-12 \le k \le 12$, $-19 \le l \le 18$	
Reflections collected	14869	
Independent reflections	4702 (R(int) = 0.0466)	
Observed reflections	2523 ($I > 2\sigma(I)$)	
Absorption correction	Semi-empirical from equivalents	
Max. and min. transmission	1.48 and 0.70	
Refinement method	Full-matrix least-squares on F^2	
Data/restraints/parameters	4702 / 459 / 523	
Goodness-of-fit on F^2	1.14	
Final R indices (I>2sigma(I))	R1 = 0.102, wR2 = 0.288	
Largest diff. peak and hole	0.47 and -0.68 $eÅ^{-3}$	

Table S8. Crystal structure, crystal data and structure refinement of **S7** (CCDC 2044187).

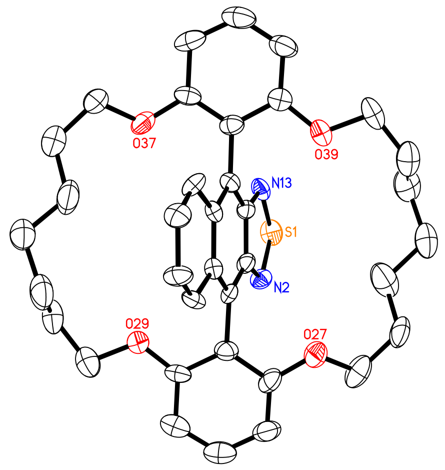

Identification code	CCDC 2044187	
Empirical formula	$C_{36}H_{38}N_2O_4S$	
Formula weight	594.74	
Temperature	200(2) K	
Wavelength	1.54178 Å	
Crystal system	monoclinic	
Space group	$P2_1$	
Z	2	
Unit cell dimensions	a = 8.3208(3) Å	α = 90 deg.
	b = 18.4339(10) Å	β = 99.993(3) deg.
	c = 9.9957(4) Å	γ = 90 deg.
Volume	1509.93(12) $Å^3$	
Density (calculated)	1.31 g/cm^3	
Absorption coefficient	1.30 mm^{-1}	
Crystal shape	plank	
Crystal size	0.095 x 0.076 x 0.022 mm^3	
Crystal colour	orange	
Theta range for data collection	4.5 to 67.2 deg.	
Index ranges	$-4 \leq h \leq 9$, $-22 \leq k \leq 20$, $-11 \leq l \leq 11$	
Reflections collected	9474	
Independent reflections	4356 (R(int) = 0.0471)	
Observed reflections	2827 ($I > 2\sigma(I)$)	
Absorption correction	Semi-empirical from equivalents	
Max. and min. transmission	1.49 and 0.69	
Refinement method	Full-matrix least-squares on F^2	
Data/restraints/parameters	4356 / 495 / 453	
Goodness-of-fit on F^2	0.99	
Final R indices (I>2sigma(I))	R1 = 0.049, wR2 = 0.094	
Absolute structure parameter	0.41(5)	
Largest diff. peak and hole	0.31 and -0.19 $eÅ^{-3}$	

Table S9. Crystal structure, crystal data and structure refinement of **S8** (CCDC 2044188).

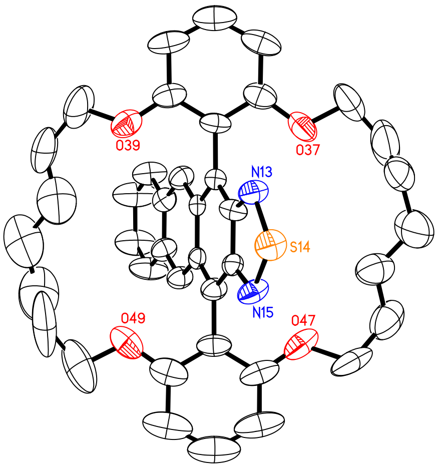

Identification code	CCDC 2044188		
Empirical formula	$C_{40}H_{40}N_2O_4S$		
Formula weight	644.80		
Temperature	200(2) K		
Wavelength	1.54178 Å		
Crystal system	tetragonal		
Space group	$I\bar{4}$		
Z	8		
Unit cell dimensions	a = 28.5703(19) Å	α =	90 deg.
	b = 28.5703(19) Å	β =	90 deg.
	c = 8.0905(10) Å	γ =	90 deg.
Volume	6604.0(12) $Å^3$		
Density (calculated)	1.30 g/cm^3		
Absorption coefficient	1.23 mm^{-1}		
Crystal shape	needle		
Crystal size	0.120 x 0.018 x 0.015 mm^3		
Crystal colour	blue		
Theta range for data collection	4.9 to 51.9 deg.		
Index ranges	$-28 \leq h \leq 29$, $-20 \leq k \leq 29$, $-7 \leq l \leq 8$		
Reflections collected	14708		
Independent reflections	3584 (R(int) = 0.1777)		
Observed reflections	1845 ($I > 2\sigma(I)$)		
Absorption correction	Semi-empirical from equivalents		
Max. and min. transmission	1.40 and 0.75		
Refinement method	Full-matrix least-squares on F^2		
Data/restraints/parameters	3584 / 575 / 425		
Goodness-of-fit on F^2	1.01		
Final R indices (I>2sigma(I))	R1 = 0.077, wR2 = 0.150		
Absolute structure parameter	0.15(9)		
Largest diff. peak and hole	0.34 and -0.23 $eÅ^{-3}$		

SUPPORTING INFORMATION

Table S10. Crystal structure, crystal data and structure refinement of **S3** (CCDC 2044185).

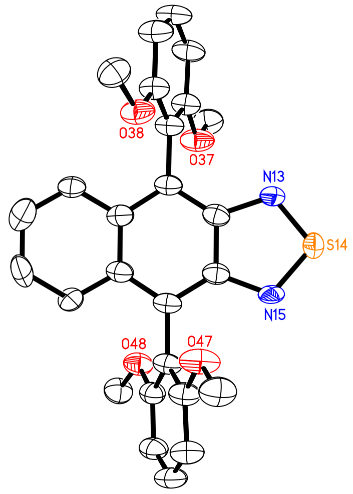

Identification code	CCDC 2044185	
Empirical formula	$C_{26}H_{22}N_2O_4S$	
Formula weight	458.51	
Temperature	200(2) K	
Wavelength	1.54178 Å	
Crystal system	monoclinic	
Space group	$P2_1/c$	
Z	6	
Unit cell dimensions	a = 20.5671(9) Å	α = 90 deg.
	b = 7.5774(2) Å	β = 92.846(4) deg.
	c = 20.8300(9) Å	γ = 90 deg.
Volume	3242.2(2) $Å^3$	
Density (calculated)	1.41 g/cm^3	
Absorption coefficient	1.64 mm^{-1}	
Crystal shape	plank	
Crystal size	0.090 x 0.040 x 0.028 mm^3	
Crystal colour	orange	
Theta range for data collection	4.2 to 72.0 deg.	
Index ranges	$-25 \le h \le 24$, $-4 \le k \le 9$, $-24 \le l \le 25$	
Reflections collected	21067	
Independent reflections	6130 (R(int) = 0.0647)	
Observed reflections	3433 ($I > 2\sigma(I)$)	
Absorption correction	Semi-empirical from equivalents	
Max. and min. transmission	1.48 and 0.69	
Refinement method	Full-matrix least-squares on F^2	
Data/restraints/parameters	6130 / 1052 / 551	
Goodness-of-fit on F^2	1.04	
Final R indices (I>2sigma(I))	R1 = 0.060, wR2 = 0.103	
Largest diff. peak and hole	0.29 and -0.33 $eÅ^{-3}$	

Table S11. Crystal structure, crystal data and structure refinement of **S4** (CCDC 2044186).

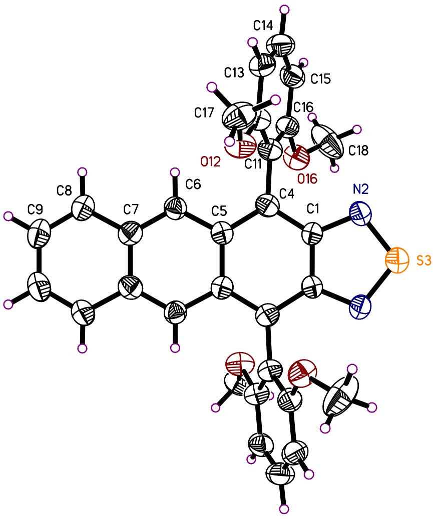

Identification code	CCDC 2044186		
Empirical formula	$C_{30}H_{24}N_2O_4S$		
Formula weight	508.57		
Temperature	200(2) K		
Wavelength	1.54178 Å		
Crystal system	Orthorhombic		
Space group	Pbcn		
Z	4		
Unit cell dimensions	a = 19.055(2) Å	α =	90 deg.
	b = 16.1660(12) Å	β =	90 deg.
	c = 7.7352(6) Å	γ =	90 deg.
Volume	2382.8(4) $Å^3$		
Density (calculated)	1.42 g/cm^3		
Absorption coefficient	1.55 mm^{-1}		
Crystal shape	needle		
Crystal size	0.178 x 0.015 x 0.014 mm^3		
Crystal colour	violet		
Theta range for data collection	4.6 to 69.2 deg.		
Index ranges	$-21\le h\le 22, -19\le k\le 16, -9\le l\le 4$		
Reflections collected	8919		
Independent reflections	2191 (R(int) = 0.1461)		
Observed reflections	984 (I > 2\s(I))		
Absorption correction	Semi-empirical from equivalents		
Max. and min. transmission	0.98 and 0.39		
Refinement method	Full-matrix least-squares on F^2		
Data/restraints/parameters	2191 / 0 / 170		
Goodness-of-fit on F^2	0.90		
Final R indices ($I>2\sigma(I)$)	R1 = 0.048, wR2 = 0.073		
Largest diff. peak and hole	0.27 and -0.28 $eÅ^{-3}$		

Table S12. Crystal structure, crystal data and structure refinement of **S10** (CCDC 2044189).

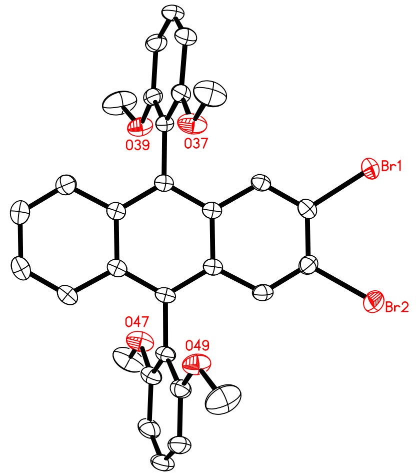

Identification code	CCDC 2044189	
Empirical formula	$C_{30}H_{24}Br_2O_4$	
Formula weight	608.31	
Temperature	200(2) K	
Wavelength	0.71073 Å	
Crystal system	monoclinic	
Space group	$P2_1/c$	
Z	4	
Unit cell dimensions	a = 16.3942(10) Å	α = 90 deg.
	b = 7.8249(5) Å	β = 92.6999(10) deg.
	c = 20.2080(12) Å	γ = 90 deg.
Volume	2589.5(3) $Å^3$	
Density (calculated)	1.56 g/cm^3	
Absorption coefficient	3.16 mm^{-1}	
Crystal shape	plank	
Crystal size	0.290 x 0.067 x 0.029 mm^3	
Crystal colour	colourless	
Theta range for data collection	2.0 to 30.1 deg.	
Index ranges	$-23\leq h\leq 23$, $-11\leq k\leq 11$, $-27\leq l\leq 28$	
Reflections collected	56361	
Independent reflections	7600 (R(int) = 0.0473)	
Observed reflections	5937 ($I > 2\sigma(I)$)	
Absorption correction	Semi-empirical from equivalents	
Max. and min. transmission	0.93 and 0.82	
Refinement method	Full-matrix least-squares on F^2	
Data/restraints/parameters	7600 / 0 / 338	
Goodness-of-fit on F^2	1.05	
Final R indices (I>2sigma(I))	R1 = 0.037, wR2 = 0.080	
Largest diff. peak and hole	0.74 and -0.76 $eÅ^{-3}$	

Table S13. Crystal structure, crystal data and structure refinement of 2-bromo-9,10-bis(2,6-dimethoxyphenyl)anthracene (CCDC 2044190).

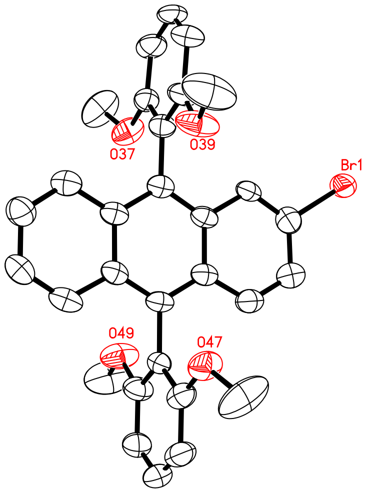

Identification code	CCDC 2044190	
Empirical formula	$C_{30}H_{25}BrO_4$	
Formula weight	529.41	
Temperature	200(2) K	
Wavelength	0.71073 Å	
Crystal system	monoclinic	
Space group	Pc	
Z	2	
Unit cell dimensions	a = 10.9267(3) Å	α = 90 deg.
	b = 14.3715(4) Å	β = 93.6066(14) deg.
	c = 7.9524(2) Å	γ = 90 deg.
Volume	1246.32(6) $Å^3$	
Density (calculated)	1.41 g/cm^3	
Absorption coefficient	1.68 mm^{-1}	
Crystal shape	plank	
Crystal size	0.200 x 0.098 x 0.054 mm^3	
Crystal colour	orange	
Theta range for data collection	1.4 to 25.1 deg.	
Index ranges	$-13 \le h \le 12$, $-17 \le k \le 17$, $-9 \le l \le 9$	
Reflections collected	11461	
Independent reflections	4408 (R(int) = 0.0349)	
Observed reflections	3763 ($I > 2\sigma(I)$)	
Absorption correction	Semi-empirical from equivalents	
Max. and min. transmission	0.86 and 0.78	
Refinement method	Full-matrix least-squares on F^2	
Data/restraints/parameters	4408 / 279 / 341	
Goodness-of-fit on F^2	1.07	
Final R indices (I>2sigma(I))	R1 = 0.058, wR2 = 0.136	
Absolute structure parameter	0.448(5)	
Largest diff. peak and hole	0.25 and -0.46 $eÅ^{-3}$	

References

[S1] G. R. Fulmer, A. J. M. Miller, N. H. Sherden, H. E. Gottlieb, A. Nudelman, B. M. Stoltz, J. E. Bercaw, K. I. Goldberg, *Organometallics* **2010**, *29*, 2176-2179.

[S2] *Gaussian 16, Revision C.01,* M. J. Frisch, G. W. Trucks, H. B. Schlegel, G. E. Scuseria, M. A. Robb, J. R. Cheeseman, G. Scalmani, V. Barone, G. A. Petersson, H. Nakatsuji, X. Li, M. Caricato, A. V. Marenich, J. Bloino, B. G. Janesko, R. Gomperts, B. Mennucci, H. P. Hratchian, J. V. Ortiz, A. F. Izmaylov, J. L. Sonnenberg, D. Williams-Young, F. Ding, F. Lipparini, F. Egidi, J. Goings, B. Peng, A. Petrone, T. Henderson, D. Ranasinghe, V. G. Zakrzewski, J. Gao, N. Rega, G. Zheng, W. Liang, M. Hada, M. Ehara, K. Toyota, R. Fukuda, J. Hasegawa, M. Ishida, T. Nakajima, Y. Honda, O. Kitao, H. Nakai, T. Vreven, K. Throssell, J. A. Montgomery, Jr., J. E. Peralta, F. Ogliaro, M. J. Bearpark, J. J. Heyd, E. N. Brothers, K. N. Kudin, V. N. Staroverov, T. A. Keith, R. Kobayashi, J. Normand, K. Raghavachari, A. P. Rendell, J. C. Burant, S. S. Iyengar, J. Tomasi, M. Cossi, J. M. Millam, M. Klene, C. Adamo, R. Cammi, J. W. Ochterski, R. L. Martin, K. Morokuma, O. Farkas, J. B. Foresman, and D. J. Fox, Gaussian, Inc., Wallingford CT, **2016**.

[S3] P. V. Hatcher, J. H. Reibenspies, R. C. Haddon, D. Li, N. Lopez, X. Chi, *CrystEngComm* **2015**, *17*, 4172-4178.

[S4] S. Miao, S. M. Brombosz, P. v. R. Schleyer, J. I. Wu, S. Barlow, S. R. Marder, K. I. Hardcastle, U. H. F. Bunz, *J. Am. Chem. Soc.* **2008**, *130*, 7339.

[S5] A. L. Appleton, S. Miao, S. M. Brombosz, N. J. Berger, S. Barlow, S. R. Marder, B. M. Lawrence, K. I. Hardcastle, Uwe H. F. Bunz, *Org. Lett.* **2009**, *11*, 5222–5225.

[S6] D. Bailey, V. E. Williams, *Tetrahedron Lett*. **2004**, *45*, 2511-2513.

[S7] a) J. E. Anthony, J. S. Brooks, D. L. Eaton, S. R. Parkin, *J. Am. Chem. Soc*. **2001**, *123*, 9482-9483; b) O. Tverskoy, F. Rominger, A. Peters, H.-J. Himmel, U. H. F. Bunz, *Angew. Chem., Int. Ed*. **2011**, *50*, 3557-3560; c) J. U. Engelhart, B. D. Lindner, O. Tverskoy, F. Rominger, U. H. F. Bunz, *Chem. Eur. J.* **2013**, *19*, 15089-15092; d) A. L. Appleton, S. M. Brombosz, S. Barlow, J. S. Sears, J.-L. Bredas, S. R. Marder, U. H. F. Bunz, *Nat. Commun*. **2010**, *1*, 1-6; e) T. Wiesner, L. Ahrens, F. Rominger, J. Freudenberg, U. H. F. Bunz, manuscript submitted to *Chem. Eur. J.*

[S8] B. D. Lindner, J. U. Engelhart, O. Tverskoy, A. L. Appleton, F. Rominger, A. Peters, H.-J. Himmel, U. H. F. Bunz, *Angew. Chem. Int. Ed.* **2011**, *50*, 8588-8591.

[S9] C. M. Cardona, W. Li, A. E. Kaifer, D. Stockdale, G. C. Bazan, *Adv. Mater.* **2011**, *23*, 2367-2371.

[S10] S. Kazim, F. J. Ramos, P. Gao, M. K. Nazeeruddin, M. Grätzel S. Ahmad*, Energy Environ. Sci.* **2015**, *8*, 1816-1823.

Author Contributions

Lukas Ahrens: Synthesis, analysis, stability studies, writing of original draft (lead)
Olena Tverskoy: Synthesis of precursors (supporting)
Svenja Weigold: Decomposition experiments (supporting)
Michael Ganschow: Synthesis of precursors (supporting)
Frank Rominger: Crystal structure elucidation (supporting)
Jan Freudenberg: Project administration, editorial work (supporting)
Uwe H. F. Bunz: Writing of original draft, project administration, funding acquisition (lead)
